# Iron-Catalyzed Sulfonylmethylation of Imidazo[1,2-α]pyridines with *N*,*N*-Dimethylacetamide and Sodium Sulfinates

**DOI:** 10.3390/molecules29133196

**Published:** 2024-07-05

**Authors:** Shengnan Sun, Hexia Ye, Haibo Liu, Junchen Li, Xiaojing Bi

**Affiliations:** State Key Laboratory of NBC Protection for Civilian, Beijing 102205, China; shengnansunmail@yeah.net (S.S.); yehexia6688@yeah.net (H.Y.)

**Keywords:** sulfonylmethylation, imidazo[1,2-α]pyridine, DMA, FeCl_3_-catalyzed

## Abstract

Functionalized imidazo[1,2-α]pyridines are important scaffolds in pharmaceuticals. Herein, we present an efficient 3-sulfonylmethylation protocol for imidazo[1,2-α]pyridines by sodium sulfinates in DMA and H_2_O (2:1) via an FeCl_3_-catalyzed three-component coupling reaction. Various sulfonylmethyl imidazo[1,2-α]pyridines were thus afforded in high yields with excellent functional group tolerance. A plausible oxidation-addition mechanism was proposed.

## 1. Introduction

As a kind of privileged scaffold, imidazo[1,2-α]pyridines are often used as the pharmacodynamic backbones of versatile drugs, such as anti-ulcer [1], anti-inflammatory [2], anti-viral [3,4], anti-bacterial [5], anti-cancer [6], anti-HIV [7] and anti-tuberculosis pharmaceutical candidates [8]. Owing to the electron-rich nature of imidazo[1,2-α]pyridine, the functionalization of imidazo[1,2-α]pyridine, especially C3-positioned C-C and C-X (O/N/S) bond construction, has attracted widespread attention [9,10,11,12,13,14,15,16,17,18,19,20,21,22,23,24,25,26]. Nevertheless, imidazo[1,2-α]pyridines containing sulfur functionality, such as sulfonylmethylated imidazo[1,2-α]pyridines, have been rarely studied before, whilst the sulfones group exists widely in drug molecules. The first typical example reported by Song employed the iron-involved tosylmethylation of imidazo[1,2-α]pyridines with *p*-toluenesulfonyl methyl isocyanide in H_2_O and PEG_400_ [27]. Then, Rode described a selectfluor-mediated approach regarding the methylene-tethered arylsulfonation and benzotriazolation of imidazopyridines using DMSO as the carbon insertion synthon [28]. A very recent study reported by Tang demonstrated another efficient protocol for the direct C-3 sulfonylmethylation of imidazo[1,2-α]pyridines with glyoxylic acid and sodium sulfinates in water [29]. Some studies that have used DMA as a carbon source have been reported for the coupling of C-C bonds [30,31,32]. However, a new methodology enabling the C-H sulfonylmethylation of imidazo[1,2-α]pyridines is still highly desirable and valuable.

**Figure 1:**
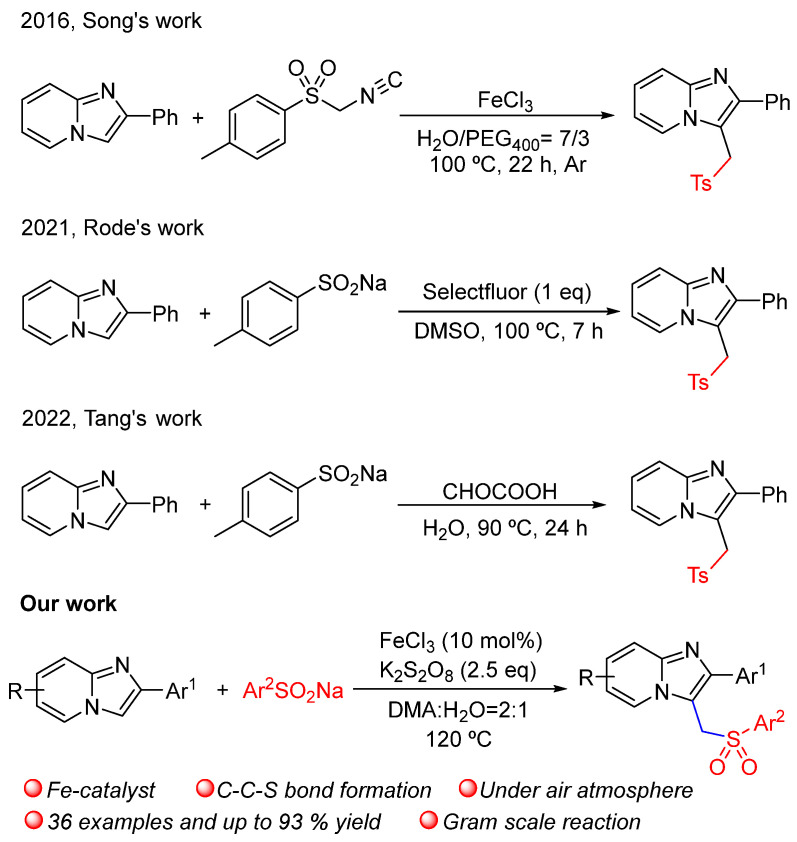
Comparison of recently reported sulfonylmethylation of imidazo[1,2-α]pyridines, refs. [27,28,29].

In our previous work, sodium sulfinates were found to be an effective and universal sulfur source in eco-friendly S-S and S-C bond construction [33]. In our following explorations, the unexpected sulfonylmethylation of imidazo[1,2-α]pyridines was observed when researchers tried to synthesize sulfones in DMA. Herein, we wish to present a new method for the iron-catalyzed sulfonylmethylation of imidazo[1,2-α]pyridines with DMA and sodium sulfinates to synthesize 3-sulfonylmethyl imidazo[1,2-α]pyridines (Figure 1).

## 2. Results and Discussion

The optimization of reaction conditions was carried out by using 2-phenylimidazo[1,2-α]pyridine (**1a**) as the model substrate, sodium *p*-toluenesulfinate (**2a**) as the sulfonylation reagent and DMA as the carbon synthon. During the early stages of this work, we mainly focused on different metal catalysts using K_2_S_2_O_8_ as an oxidant. The results showed that both K_2_S_2_O_8_ and the catalyst were essential for the transformation (Table 1, entries 1–3). Among the metal catalysts screened, including FeCl_3_, FeCl_2_, Fe_3_O_4_, CuI, Cu(acac)_2_, V(acac)_2_ and AgNO_3_, the best conversion with a 73% yield was obtained by FeCl_3_ in the DMA and H_2_O (2:1) hybrid solvent (Table 1, entries 4–10). As for the FeCl_3_ catalyst, 10 mol% loading was effective enough to give the highest productivity of 73% product. Reducing to 5 mol% FeCl_3_ gave comparable yields (Table 1, entries 6, 11–13). No products were obtained when the K_2_S_2_O_8_ oxidant was changed to H_2_O_2_, TBHP, O_2_ or I_2_O_5_ (Table 1, entries 14–17). The amount of K_2_S_2_O_8_ added was also shown to have some effects on the yields, and 2.5 eq. was optimal (Table 1, entries 6, 18–20).

Further screening demonstrated that H_2_O is vitally important, probably due to the fact that the low solubility of sodium sulfinate in DMA would lead to insufficient sulfur–reactant participation in a reaction without water (Table 1, entries 21–25). The best ratio of DMA and H_2_O is 2:1. To our delight, when increasing the loading of **2a**, up to 90% yield was achieved (Table 1, entries 26–28). Furthermore, the reaction gave better results at higher temperatures such as 120 °C (Table 1, entries 29–32). Screening of other typical carbon source reagents, including DMF, DMSO and TMEDA, showed that DMA was the best (Table 1, entries 33–35).

With the optimum condition in hand (Table 1, entry 31), we then evaluated the scope of structurally different sodium sulfinates with 2-phenylimidazo[1,2-α]pyridin (Table 2). As shown in Table 2, sodium sulfinates bearing electron-donating (4-Me, 3-Me, 4-iPr, 4-OMe) or electron-withdrawing (4-CF_3_, 4-F, 4-Cl, 2-Cl, 4-Br) groups at the ortho, meta, or para positions were all compatible, affording good to excellent yields (Table 2, **3a**–**3j**). Moreover, sodium aromatic heterocyclic sulfinate also tolerated well (Table 2, **3k**–**3l**). To our surprise, this protocol was also applicable to sodium alkyl sulfinate, giving the target compounds in good yields (Table 2, **3m**–**3n**). Regrettably, no product was generated when sodium triflate and sodium difluoromethanesulfinate were used (Table 2, **3o**–**3p**).

We next turned our attention to imidazo[1,2-α]pyridines substrates bearing different substituents (Table 3). To our delight, the reaction of sulfonylmethylation could be smoothly accomplished with 2-(4-bromophenyl)imidazo[1,2-α]pyridine under the standard conditions, offering compound **3qa**–**3qf** in good yields. This protocol can be effectively applied to 2-phenylimidazo-[1,2-α]pyridine with electron-donating groups (2-Me, 3-Me, 4-OCH_3_) and electron-withdrawing groups (4-NO_2_, 4-F, 4-Cl) (Table 3, **3ra**–**3rf**). Imidazo[1,2-α]pyridine substituted in the 6, 7, and 8 positions was also well tolerated (Table 3, **3sa**–**3se**). It is worth mentioning that heterocyclic substrates were amenable to this protocol, affording the desired product with good yields (Table 3, **3ta**–**3ud**). However, some substrates are not suitable for this protocol (Table 3, **3v**–**3y**).

**Figure 2:**
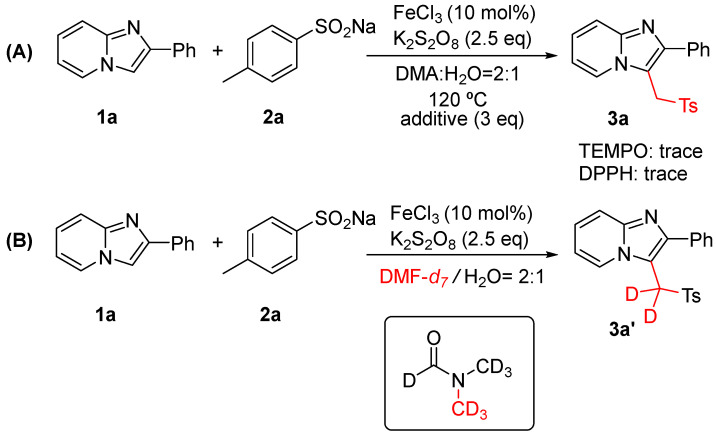
Control experiments. (**A**) Free radical trapping experiment; (**B**) Isotope labeling experiment.

In order to investigate the mechanism of the reaction, control experiments were explored, and the results are illustrated in Figure 2. Nearly no product was detected when 3 eq. radical scavenger (2,2,6,6-tetramethylpiperidin-1-oxyl (TEMPO) or 2,2-diphenyl-1-picrylhydrazyl (DPPH)) was added to the reaction system (Figure 2A), suggesting that a radical pathway might be involved in this protocol. Isotopic labeling experiments clearly showed that DMF provided the carbon attached to the imidazo heterocyclic backbone (Figure 2B).

**Figure 3:**
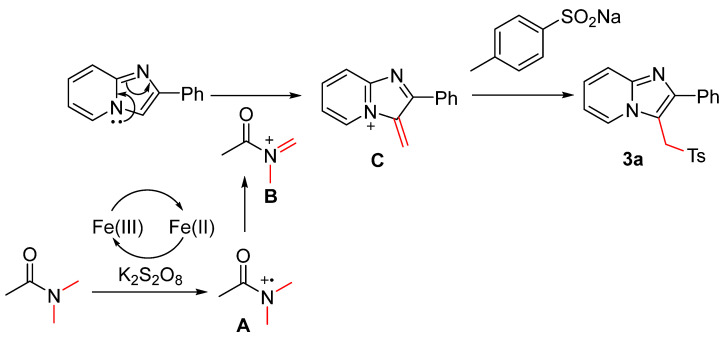
Proposed mechanism.

According to our control experiments and the previous literature [11], a plausible mechanism of the sulfonylmethylation of imidazo[1,2-α]pyridines with DMA and sodium sulfinates is illustrated (Figure 3). DMA was firstly oxidized to iminium **B** by FeCl_3_/K_2_S_2_O_8_, which then reacted with electron-rich imidazopyridine to form intermediate **C** by C-C coupling. The sulfonylation of **C** by sodium *p*-toluenesulfinate generated 2-phenyl-3-(tosylmethyl)imidazo[1,2-α]pyridine (**3a**).

## 3. Experimental Section

General Information. All reactions were performed in a 10 mL tube with magnetic stirring. The imidazo[1,2-α] pyridines and sodium sulfinates are commercially available. Unless otherwise stated, all commercially available reagents were used without further purification. High-resolution mass spectra (ESI) were obtained with the Waters Xevo G2-XS QTOF. ^1^H NMR, ^13^C NMR and ^19^F NMR spectra were recorded at ambient temperature on Bruker 300 M instruments. All spectra were referenced to CDCl_3_ (^1^H NMR δ 7.26 ppm and ^13^C NMR δ 77.00 ppm). Data were reported as follows: chemical shift, multiplicity (s = singlet, d = doublet, t = triplet, q = quartet, dd = doublet of doublets, td = triplet of doublets, qd = quartet of doublets, m = multiplet), coupling constants (Hz) and integration.

Experimental Procedure. In a typical experiment, a 15 mL sealed tube was charged with imidazo[1,2-α]pyridine, (0.6 mmol), substituted sodium sulfinate (1.2 mmol), DMA (4 mL), H_2_O (2 mL), K_2_S_2_O_8_ (2.5 eq) and FeCl_3_ (10 mol%). The mixture was allowed to stir at 120 °C and was monitored by TLC until the reaction was complete. Saturated aqueous NaCl solution was added to the reaction mixture and the aqueous phase was further extracted with ethyl acetate (3 × 10 mL). The combined organic layers were dried over anhydrous Na_2_SO_4_ and concentrated under a vacuum to give the crude product. The residue was purified by column chromatography on silica gel using ethyl acetate/*n*-hexane (1:1) as the eluent to provide the desired product.

2-Phenyl-3-(tosylmethyl)imidazo[1,2-α] pyridine (**3a**) [29]. White solid, 195 mg, 90% yield, ethyl acetate/*n*-hexane (1:1). ^1^H NMR (300 MHz, CDCl_3_) δ 8.45 (d, *J* = 6.9 Hz, 1H), 7.67 (d, *J* = 9.1 Hz, 1H), 7.38 (d, *J* = 8.3 Hz, 2H), 7.35–7.27 (m, 6H), 7.09 (d, *J* = 8.0 Hz, 2H), 6.94 (s, 1H), 4.88 (s, 2H), 2.36 (s, 3H). ^13^C{1H} NMR (75 MHz, CDCl_3_) δ 147.42, 146.00, 145.33, 134.22, 133.03, 129.84, 128.34, 128.23, 128.16, 128.14, 126.03, 124.98, 117.50, 112.92, 108.29, 52.69, 21.67.

2-Phenyl-3-((*m*-tolylsulfonyl)methyl)imidazo[1,2-α]pyridine (**3b**) [29]. White solid, 185 mg, 85% yield, ethyl acetate/*n*-hexane (1:1). ^1^H NMR (300 MHz, CDCl_3_) δ 8.45 (d, *J* = 7.0 Hz, 1H), 7.71 (d, *J* = 9.0 Hz, 1H), 7.31 (td, *J* = 7.4, 6.8, 3.9 Hz, 9H), 7.23 (d, *J* = 7.6 Hz, 1H), 6.96 (td, *J* = 6.9, 1.2 Hz, 1H), 4.88 (s, 2H), 2.26 (s, 3H). ^13^C{1H} NMR (75 MHz, CDCl_3_) δ 147.60, 146.02, 139.79, 137.35, 135.10, 132.97, 129.14, 128.53, 128.50, 128.34, 128.28, 126.14, 125.42, 124.99, 117.58, 113.03, 108.20, 52.76, 21.30.

2-Phenyl-3-((phenylsulfonyl)methyl)imidazo[1,2-α]pyridine (**3c**) [29]. White solid, 184 mg, 88% yield. ethyl acetate/*n*-hexane (1:1). ^1^H NMR (300 MHz, CDCl_3_) δ 8.47 (d, *J* = 7.0 Hz, 1H), 7.71 (d, *J* = 9.0 Hz, 1H), 7.59–7.52 (m, 3H), 7.39–7.29 (m, 8H), 6.96 (td, *J* = 6.9, 1.2 Hz, 1H), 4.91 (s, 2H). ^13^C{1H} NMR (75 MHz, CDCl_3_) δ 147.67, 146.04, 137.51, 134.26, 132.99, 129.28, 128.55, 128.28, 128.25, 128.19, 126.08, 124.96, 117.57, 112.97, 108.03, 52.74.

3-(((4-Isopropylphenyl)sulfonyl)methyl)-2-phenylimidazo [1,2-α]pyridine (**3d**) [29]. White solid, 215 mg, 92% yield, ethyl acetate/*n*-hexane (1:1), ^1^H NMR (300 MHz, CDCl_3_) δ 8.44 (d, *J* = 7.0 Hz, 1H), 7.68 (d, *J* = 9.1 Hz, 1H), 7.44 (d, *J* = 8.4 Hz, 2H), 7.35–7.26 (m, 6H), 7.18 (d, *J* = 8.3 Hz, 2H), 6.97–6.89 (m, 1H), 4.87 (s, 2H), 2.97–2.83 (p, *J* = 6.9 Hz, 1H), 1.22 (d, *J* = 6.9 Hz, 6H). ^13^C{1H} NMR (75 MHz, CDCl_3_) δ 155.95, 147.53, 145.99, 134.74, 133.05, 128.49, 128.39, 128.23, 128.20, 127.42, 126.01, 125.01, 117.50, 112.89, 108.31, 52.80, 34.26, 23.61.

3-(((4-Methoxyphenyl)sulfonyl)methyl)-2-phenylimidazo [1,2-α]pyridine (**3e**) [29]. White solid, 205 mg, 90% yield. ethyl acetate/*n*-hexane (1:1). ^1^H NMR (300 MHz, CDCl_3_) δ 8.36 (d, *J* = 7.0 Hz, 1H), 7.59 (d, *J* = 9.1 Hz, 1H), 7.34–7.28 (m, 2H), 7.26–7.18 (m, 6H), 6.85 (td, *J* = 6.8, 1.2 Hz, 1H), 6.69–6.58 (m, 2H), 4.79 (s, 2H), 3.72 (s, 3H). ^13^C{1H} NMR (75 MHz, CDCl_3_) δ 164.10, 147.20, 145.90, 132.99, 130.30, 128.37, 128.15, 128.10, 125.99, 124.92, 117.47, 114.34, 112.90, 108.47, 55.63, 52.68.

3-(((4-Fluorophenyl)sulfonyl)methyl)-2-phenylimidazo[1,2-α]pyridine (**3f**) [29]. White solid, 171 mg, 78% yield, ethyl acetate/*n*-hexane (1:1). ^1^H NMR (300 MHz, CDCl_3_) δ 8.36 (d, *J* = 6.9 Hz, 1H), 7.64 (d, *J* = 9.0 Hz, 1H), 7.29–7.23 (m, 5H), 7.22–7.10 (m, 5H), 6.92 (td, *J* = 6.9, 1.2 Hz, 1H), 4.87 (s, 2H). ^13^C{1H} NMR (75 MHz, CDCl_3_) δ 167.79, 164.38, 147.38, 146.08, 132.95, 131.07, 130.93, 128.52, 128.26, 128.06, 126.11, 124.84, 117.60, 116.57, 116.27, 112.99, 107.91, 52.35. ^19^F NMR (282 MHz, CDCl_3_) δ-102.63.

3-(((4-Chlorophenyl)sulfonyl)methyl)-2-phenylimidazo[1,2-α]pyridine (**3g**) [29]. White solid, 183 mg, 80% yield, ethyl acetate/*n*-hexane (1:1). ^1^H NMR (300 MHz, CDCl_3_) δ 8.43 (d, *J* = 6.8 Hz, 1H), 7.77–7.67 (m, 1H), 7.40–7.28 (m, 6H), 7.25 (s, 2H), 7.21–7.14 (m, 2H), 6.97 (d, *J* = 6.8 Hz, 1H), 4.93 (s, 2H). ^13^C{1H} NMR (75 MHz, CDCl_3_) δ 147.06, 145.93, 141.27, 135.26, 132.50, 129.60, 129.48, 128.66, 128.51, 128.14, 126.61, 124.95, 117.60, 113.40, 107.98, 52.27.

3-(((2-Chlorophenyl)sulfonyl)methyl)-2-phenylimidazo[1,2-α]pyridine (**3h**) [29]. White solid, 170 mg, 74% yield. ethyl acetate/*n*-hexane (1:1). ^1^H NMR (300 MHz, CDCl_3_) δ 8.48 (d, *J* = 7.0 Hz, 1H), 7.73 (dd, *J* = 7.9, 1.7 Hz, 1H), 7.66 (d, *J* = 9.1 Hz, 1H), 7.44 (d, *J* = 3.8 Hz, 3H), 7.35–7.27 (m, 5H), 7.24 (dd, *J* = 7.9, 1.2 Hz, 1H), 6.95 (td, *J* = 6.9, 1.2 Hz, 1H), 5.14 (s, 2H). ^13^C{1H} NMR (75 MHz, CDCl_3_) δ 148.07, 146.22, 135.41, 135.27, 133.08, 132.91, 132.04, 132.01, 128.67, 128.52, 128.41, 127.37, 126.24, 125.10, 117.59, 113.07, 107.48, 50.37.

3-(((4-Bromophenyl)sulfonyl)methyl)-2-phenylimidazo[1,2-α]pyridine (**3i**) [29]. White solid, 210 mg, 82% yield, ethyl acetate/*n*-hexane (1:1). ^1^H NMR (300 MHz, CDCl_3_) δ 8.39 (d, *J* = 6.9 Hz, 1H), 7.65 (d, *J* = 9.1 Hz, 1H), 7.31 (ddd, *J* = 7.8, 4.0, 2.3 Hz, 6H), 7.26–7.15 (m, 4H), 6.93 (td, *J* = 6.8, 1.2 Hz, 1H). ^13^C{1H} NMR (75 MHz, CDCl_3_) δ 147.37, 146.11, 135.75, 132.77, 132.36, 129.91, 129.56, 128.57, 128.32, 128.06, 126.26, 124.84, 117.63, 113.13, 107.82, 52.17.

2-Phenyl-3-(((4-(trifluoromethyl)phenyl)sulfonyl)methyl) imidazo[1,2-α]pyridine (**3j**). White solid, 188 mg, 75% yield. ethyl acetate/*n*-hexane (1:1), mp. 120–121 °C. ^1^H NMR (300 MHz, CDCl_3_) δ 8.37 (d, *J* = 6.9 Hz, 1H), 7.66–7.60 (m, 1H), 7.40 (d, *J* = 1.0 Hz, 4H), 7.33–7.26 (m, 1H), 7.21–7.12 (m, 5H), 6.92 (d, *J* = 6.1 Hz, 1H), 4.93 (s, 2H). ^13^C{1H} NMR (75 MHz, CDCl_3_) δ 147.45, 146.20, 140.44, 132.74, 128.76, 128.68, 128.32, 127.92, 126.30, 126.11, 126.06, 124.82, 121.18, 117.70, 113.15, 107.42, 51.93. HRMS (ESI) m/z: [M + H]^+^ Calcd for C_21_H_16_F_3_N_2_O_2_S^+^ 417.0880, Found 417.0881.

2-Phenyl-3-((thiophen-2-ylsulfonyl)methyl)imidazo [1,2-α] pyridine (**3k**) [29]. White solid 166 mg, 78% yield, ethyl acetate/*n*-hexane (1:1). ^1^H NMR (300 MHz, CDCl_3_) δ 8.41 (d, *J* = 6.9 Hz, 1H), 7.69 (d, *J* = 9.1 Hz, 1H), 7.57 (dd, *J* = 5.0, 1.3 Hz, 1H), 7.45–7.37 (m, 2H), 7.37–7.27 (m, 5H), 6.98–6.90 (m, 2H), 4.99 (s, 2H). ^13^C{1H} NMR (75 MHz, CDCl_3_) δ 147.91, 146.12, 138.00, 135.41, 135.07, 132.98, 128.65, 128.41, 128.35, 128.21, 126.24, 124.95, 117.63, 113.12, 108.11, 54.07.

2-Phenyl-3-((pyridin-2-ylsulfonyl)methyl)imidazo[1,2-α] pyridine (**3l**) [29]. White solid, 146 mg, 70% yield, ethyl acetate/*n*-hexane (1:1), mp. 108–109 °C. ^1^H NMR (300 MHz, CDCl_3_) δ 8.60–8.54 (m, 1H), 8.45 (dt, *J* = 4.6, 1.4 Hz, 1H), 7.90–7.78 (m, 3H), 7.67–7.60 (m, 2H), 7.48–7.35 (m, 5H), 7.05 (t, *J* = 6.9 Hz, 1H), 5.23 (s, 2H). ^13^C{1H} NMR (75 MHz, CDCl_3_) δ 156.10, 150.38, 147.67, 146.20, 137.96, 133.05, 128.72, 128.59, 128.37, 127.81, 126.25, 125.00, 122.90, 117.57, 113.07, 107.15, 48.81.

3-((Cyclopropylsulfonyl)methyl)-2-phenylimidazo[1,2-α] pyridine (**3m**) [29]. White solid, 149 mg, 80% yield, ethyl acetate/*n*-hexane (1:1). ^1^H NMR (300 MHz, CDCl_3_) δ 8.40 (d, *J* = 7.0 Hz, 1H), 7.79 (dd, *J* = 8.2, 1.4 Hz, 2H), 7.71 (d, *J* = 9.1 Hz, 1H), 7.52–7.38 (m, 3H), 7.36–7.28 (m, 1H), 6.93 (dd, *J* = 6.9, 1.1 Hz, 1H), 4.86 (s, 2H), 2.12 (ddd, *J* = 12.8, 8.0, 4.8 Hz, 1H), 1.00 (dd, *J* = 4.7, 2.1 Hz, 2H), 0.68 (dd, *J* = 7.9, 2.2 Hz, 2H). ^13^C{1H} NMR (75 MHz, CDCl_3_) δ 147.08, 145.99, 133.51, 129.00, 128.64, 128.53, 126.21, 124.91, 117.52, 113.08, 108.12, 49.91, 28.80, 4.98.

3-((Methylsulfonyl)methyl)-2-phenylimidazo[1,2-α]pyridine (**3n**) [29]. White solid, 141 mg, 82% yield. ethyl acetate/*n*-hexane (1:1). ^1^H NMR (300 MHz, CDCl_3_) δ 8.39 (d, *J* = 6.9 Hz, 1H), 7.75 (d, *J* = 6.8 Hz, 2H), 7.70 (d, *J* = 9.1 Hz, 1H), 7.54–7.47 (m, 2H), 7.47–7.41 (m, 1H), 7.36–7.30 (m, 1H), 6.94 (td, *J* = 6.9, 1.2 Hz, 1H), 4.83 (s, 2H), 2.63 (s, 3H). ^13^C{1H} NMR (75 MHz, CDCl_3_) δ 147.44, 146.46, 133.54, 129.29, 128.87, 128.53, 126.30, 124.94, 117.82, 113.19, 108.02, 50.95, 39.61.

2-(4-Bromophenyl)-3-(tosylmethyl)imidazo[1,2-α]pyridine (**3qa**) [29]. White solid, 221 mg, 84% yield, ethyl acetate/*n*-hexane (1:1). ^1^H NMR (300 MHz, CDCl_3_) δ 8.42 (d, *J* = 7.0 Hz, 1H), 7.68 (d, *J* = 9.1 Hz, 1H), 7.45–7.38 (m, 4H), 7.37–7.31 (m, 1H), 7.24 (d, *J* = 8.5 Hz, 2H), 7.12 (d, *J* = 7.7 Hz, 2H), 6.95 (td, *J* = 6.9, 1.2 Hz, 1H), 4.82 (s, 2H), 2.38 (s, 3H). ^13^C{1H} NMR (75 MHz, CDCl_3_) δ 146.09, 146.06, 145.65, 134.35, 132.05, 131.55, 129.95, 129.78, 128.25, 126.39, 124.93, 122.53, 117.58, 113.20, 108.48, 52.64, 21.76.

2-(4-Bromophenyl)-3-(((4-methoxyphenyl)sulfonyl)methyl) imidazo[1,2-α]pyridine (**3qb**). White solid, 243 mg, 89% yield, ethyl acetate/*n*-hexane (1:1), mp. 230–231 °C. ^1^H NMR (300 MHz, CDCl_3_) δ 8.39 (d, *J* = 7.0 Hz, 1H), 7.67–7.61 (m, 1H), 7.39 (dd, *J* = 8.7, 3.7 Hz, 4H), 7.34–7.28 (m, 1H), 7.26–7.17 (m, 2H), 6.92 (td, *J* = 6.8, 1.2 Hz, 1H), 6.73 (d, *J* = 8.9 Hz, 2H), 4.82 (s, 2H), 3.82 (s, 3H). ^13^C{1H} NMR (75 MHz, CDCl_3_) δ 164.33, 146.09, 146.03, 132.19, 131.58, 130.43, 129.76, 128.38, 126.31, 124.93, 122.45, 117.61, 114.44, 113.16, 108.69, 55.82, 52.65. HRMS (ESI) m/z: [M + H]^+^ Calcd for C_21_H_18_BrN_2_O_3_S^+^ 457.0216, Found 457.0217.

2-(4-Bromophenyl)-3-((phenylsulfonyl)methyl) imidazo[1,2-α]pyridine (**3qc**). White solid, 209 mg, 82% yield, ethyl acetate/*n*-hexane (1:1), mp. 213–214 °C. ^1^H NMR (300 MHz, CDCl_3_) δ 8.36 (d, *J* = 7.0 Hz, 1H), 7.63 (d, *J* = 9.0 Hz, 1H), 7.52 (td, *J* = 7.0, 1.4 Hz, 3H), 7.34 (qd, *J* = 7.5, 6.7, 2.0 Hz, 4H), 7.23–7.12 (m, 3H), 6.89 (d, *J* = 6.9 Hz, 1H), 4.77 (s, 2H). ^13^C{1H} NMR (75 MHz, CDCl_3_) δ 146.50, 146.15, 137.58, 134.47, 132.03, 131.76, 129.85, 129.45, 128.28, 126.42, 124.93, 122.70, 117.64, 113.24, 108.17, 52.76. HRMS (ESI) m/z: [M + H]^+^ Calcd for C_20_H_16_BrN_2_O_2_S^+^ 427.0111, Found 427.0111.

2-(4-Bromophenyl)-3-(((2-chlorophenyl)sulfonyl)methyl) imidazo[1,2-α]pyridine (**3qd**). White solid, 193 mg, 70% yield, ethyl acetate/*n*-hexane (1:1), mp. 227–228 °C. ^1^H NMR (300 MHz, CDCl_3_) δ 8.49 (d, *J* = 6.9 Hz, 1H), 7.80 (dd, *J* = 8.1, 1.6 Hz, 1H), 7.72 (d, *J* = 9.0 Hz, 1H), 7.52–7.40 (m, 5H), 7.40–7.31 (m, 3H), 7.01 (td, *J* = 6.9, 1.2 Hz, 1H), 5.11 (s, 2H). ^13^C{1H} NMR (75 MHz, CDCl_3_) δ 146.81, 146.27, 135.59, 135.46, 133.08, 132.15, 132.08, 131.86, 130.11, 127.54, 126.62, 125.10, 122.90, 117.64, 113.38, 107.61, 50.35. HRMS (ESI) m/z: [M–H]^–^ Calcd for C_20_H_13_BrClN_2_O_2_S^−^ 458.9569, Found 458.9570.

2-(4-Bromophenyl)-3-(((4-bromophenyl)sulfonyl)methyl) imidazo[1,2-α]pyridine (**3qe**). White solid, 228 mg, 75% yield, ethyl acetate/*n*-hexane (1:1), mp. 244–245 °C. ^1^H NMR (300 MHz, CDCl_3_) δ 8.39 (d, *J* = 7.0 Hz, 1H), 7.70–7.66 (m, 1H), 7.50–7.44 (m, 2H), 7.44–7.38 (m, 2H), 7.38–7.32 (m, 1H), 7.30–7.26 (m, 2H), 7.20 (d, *J* = 8.5 Hz, 2H), 6.98 (td, *J* = 6.9, 1.2 Hz, 1H), 4.88 (s, 2H). ^13^C{1H} NMR (75 MHz, CDCl_3_) δ 146.18, 135.92, 132.53, 131.81, 130.24, 129.69, 129.65, 126.63, 124.84, 122.81, 117.74, 113.44, 107.98, 52.27. HRMS (ESI) m/z: [M + H]^+^ Calcd for C_20_H_15_Br_2_N_2_O_2_S^+^ 506.9195, Found 506.9196.

2-(4-Bromophenyl)-3-((cyclopropylsulfonyl)methyl) imidazo[1,2-α]pyridine (**3qf**). White solid, 173 mg, 74% yield, ethyl acetate/*n*-hexane (1:1), mp. 204–205 °C. ^1^H NMR (300 MHz, CDCl_3_) δ 8.37 (d, *J* = 7.0 Hz, 1H), 7.74–7.64 (m, 3H), 7.64–7.57 (m, 2H), 7.35–7.28 (m, 1H), 6.93 (td, *J* = 6.9, 1.2 Hz, 1H), 4.80 (s, 2H), 2.24–2.17 (m, 1H), 1.08 (dt, *J* = 6.6, 3.3 Hz, 2H), 0.81 (tt, *J* = 8.0, 3.6 Hz, 2H). ^13^C{1H} NMR (75 MHz, CDCl_3_) δ 146.19, 146.09, 132.56, 132.11, 130.06, 126.32, 124.87, 122.94, 117.58, 113.17, 108.01, 50.00, 29.11, 5.12. HRMS (ESI) m/z: [M + H]^+^ Calcd for C_17_H_16_BrN_2_O_2_S^+^ 391.0110, Found 391.0111.

2-(*o*-Tolyl)-3-(tosylmethyl)imidazo[1,2-α]pyridine (**3ra**) [29]. White solid, 185 mg, 82%, ethyl acetate/*n*-hexane (1:1), mp.133–134 °C. ^1^H NMR (300 MHz, CDCl_3_) δ 8.44 (d, *J* = 6.9 Hz, 1H), 7.56 (d, *J* = 9.1 Hz, 1H), 7.22 (dd, *J* = 9.0, 7.1 Hz, 3H), 7.12 (td, *J* = 7.5, 1.4 Hz, 1H), 7.02 (d, *J* = 7.6 Hz, 3H), 6.94 (t, *J* = 7.3 Hz, 1H), 6.86 (td, *J* = 6.9, 1.2 Hz, 1H), 6.62 (dd, *J* = 7.5, 1.3 Hz, 1H), 4.64 (s, 2H), 2.31 (s, 3H), 1.91 (s, 3H). ^13^C{1H} NMR (75 MHz, CDCl_3_) δ 147.69, 145.66, 145.06, 137.38, 134.49, 131.82, 130.30, 129.87, 128.43, 128.05, 125.82, 125.29, 125.15, 117.48, 112.86, 109.31, 52.29, 21.67, 20.00.

2-(*m*-Tolyl)-3-(tosylmethyl)imidazo[1,2-α]pyridine (**3rb**) [29]. White solid, 199 mg, 88% yield, ethyl acetate/*n*-hexane (1:1). ^1^H NMR (300 MHz, CDCl_3_) δ 8.38 (d, *J* = 6.9 Hz, 1H), 7.59 (dd, *J* = 9.1, 1.2 Hz, 1H), 7.30 (d, *J* = 8.3 Hz, 2H), 7.25–7.20 (m, 1H), 7.09 (d, *J* = 1.3 Hz, 1H), 7.02 (d, *J* = 9.1 Hz, 5H), 6.84 (td, *J* = 6.9, 1.2 Hz, 1H), 4.79 (s, 2H), 2.27 (s, 3H), 2.23 (s, 3H). ^13^C{1H} NMR (75 MHz, CDCl_3_) δ 147.50, 145.92, 145.26, 138.09, 134.39, 132.84, 129.84, 128.95, 128.26, 128.21, 126.06, 125.22, 125.04, 117.46, 112.93, 108.27, 52.81, 21.70, 21.47.

2-(4-Methoxyphenyl)-3-(tosylmethyl)imidazo[1,2-α]pyridine (**3rc**) [29]. White solid, 212 mg, 90% yield, ethyl acetate/*n*-hexane (1:1). ^1^H NMR (300 MHz, CDCl_3_) δ 8.31 (d, *J* = 6.9 Hz, 1H), 7.56 (d, *J* = 9.0 Hz, 1H), 7.34 (d, *J* = 8.3 Hz, 2H), 7.24–7.18 (m, 3H), 7.03 (d, *J* = 7.8 Hz, 2H), 6.81 (td, *J* = 6.9, 1.2 Hz, 1H), 6.76–6.68 (m, 2H), 4.75 (s, 2H), 3.74 (s, 3H), 2.28 (s, 3H). ^13^C{1H} NMR (75 MHz, CDCl_3_) δ 159.64, 147.23, 145.81, 145.27, 134.46, 129.80, 129.44, 128.16, 125.88, 125.46, 124.80, 117.24, 113.75, 112.75, 107.63, 55.31, 52.86, 21.63.

2-(4-Fluorophenyl)-3-(tosylmethyl)imidazo[1,2-α]pyridine (**3rd**) [29]. White solid, 189 mg, 83% yield, ethyl acetate/*n*-hexane (1:1). ^1^H NMR (300 MHz, CDCl_3_) δ 8.32 (d, *J* = 7.0 Hz, 1H), 7.57 (d, *J* = 9.1 Hz, 1H), 7.33 (d, *J* = 8.3 Hz, 2H), 7.29–7.20 (m, 3H), 7.04 (d, *J* = 8.0 Hz, 2H), 6.94–6.80 (m, 3H), 4.74 (s, 2H), 2.33–2.26 (m, 3H). ^13^C{1H} NMR (75 MHz, CDCl_3_) δ 164.54, 161.25, 146.39, 145.94, 145.59, 134.49, 130.18, 130.07, 129.99, 129.21, 129.16, 128.28, 126.40, 125.04, 117.47, 115.59, 115.31, 113.18, 108.29, 52.74, 21.75.

2-(4-Chlorophenyl)-3-(tosylmethyl)imidazo[1,2-α]pyridine (**3re**) [29]. White solid, 202 mg, 85% yield. ethyl acetate/*n*-hexane (1:1). ^1^H NMR (300 MHz, CDCl_3_) δ 8.33 (d, *J* = 7.0 Hz, 1H), 7.58 (d, *J* = 9.1 Hz, 1H), 7.33 (d, *J* = 8.3 Hz, 2H), 7.26–7.16 (m, 5H), 7.04 (d, *J* = 8.0 Hz, 2H), 6.86 (t, *J* = 7.3 Hz, 1H), 4.75 (s, 2H), 2.30 (s, 3H). ^13^C{1H} NMR (75 MHz, CDCl_3_) δ 210.22, 146.05, 146.00, 145.62, 134.36, 134.30, 131.55, 129.94, 129.50, 128.59, 128.24, 126.39, 124.93, 117.53, 113.19, 108.46, 52.64, 21.72.

2-(4-Nitrophenyl)-3-(tosylmethyl)imidazo[1,2-α]pyridine (**3rf**). White solid, 183 mg, 75% yield, ethyl acetate/*n*-hexane (1:1), mp.133–134 °C. ^1^H NMR (300 MHz, CDCl_3_) δ 8.44 (d, *J* = 7.0 Hz, 1H), 8.23–8.11 (m, 2H), 7.74 (d, *J* = 9.1 Hz, 1H), 7.69–7.63 (m, 2H), 7.48 (d, *J* = 8.3 Hz, 2H), 7.43–7.37 (m, 1H), 7.16 (d, *J* = 8.0 Hz, 2H), 7.01 (td, *J* = 6.9, 1.1 Hz, 1H), 4.85 (s, 2H), 2.38 (s, 3H). ^13^C{1H} NMR (75 MHz, CDCl_3_) δ 147.47, 146.35, 145.95, 144.83, 139.70, 134.42, 130.14, 128.98, 128.33, 126.97, 125.02, 123.68, 117.89, 113.69, 109.65, 52.69, 21.74. HRMS (ESI) m/z: [M + H]^+^ Calcd for C_21_H_18_N_3_O_4_S^+^ 408.1013, Found 408.1013.

8-Methyl-2-phenyl-3-(tosylmethyl)imidazo[1,2-α]pyridine (**3sa**) [29]. White solid, 201 mg, 93% yield, ethyl acetate/*n*-hexane (1:1). ^1^H NMR (300 MHz, CDCl_3_) δ 8.21 (d, *J* = 6.8 Hz, 1H), 7.30 (d, *J* = 8.3 Hz, 2H), 7.20 (dt, *J* = 9.6, 2.8 Hz, 5H), 7.00 (d, *J* = 7.9 Hz, 3H), 6.74 (t, *J* = 6.9 Hz, 1H), 4.75 (s, 2H), 2.56 (s, 3H), 2.26 (s, 3H). ^13^C{1H} NMR (75 MHz, CDCl_3_) δ 146.96, 146.32, 145.22, 134.42, 133.25, 129.83, 128.44, 128.31, 128.19, 127.99, 127.44, 124.82, 122.74, 112.91, 108.61, 52.85, 21.68, 17.18.

7-Methyl-2-phenyl-3-(tosylmethyl)imidazo[1,2-α]pyridine (**3sb**) [29]. White solid, 199 mg, 88% yield, ethyl acetate/*n*-hexane (1:1). ^1^H NMR (300 MHz, CDCl_3_) δ 8.23 (d, *J* = 7.1 Hz, 1H), 7.34 (s, 1H), 7.27 (d, *J* = 8.3 Hz, 2H), 7.20–7.15 (m, 5H), 6.98 (d, *J* = 8.0 Hz, 2H), 6.67 (dd, *J* = 7.1, 1.6 Hz, 1H), 4.75 (s, 2H), 2.35 (d, *J* = 1.0 Hz, 3H), 2.26 (s, 3H). ^13^C{1H} NMR (75 MHz, CDCl_3_) δ 147.02, 146.35, 145.25, 137.28, 134.16, 133.04, 129.79, 128.28, 128.15, 128.02, 124.20, 115.84, 115.56, 107.65, 52.69, 21.66, 21.44.

6-Methyl-2-phenyl-3-(tosylmethyl)imidazo[1,2-α]pyridine (**3sc**) [29]. White solid, 203 mg, 90% yield. ethyl acetate/*n*-hexane (1:1). ^1^H NMR (300 MHz, CDCl_3_) δ 8.12 (s, 1H), 7.57 (d, *J* = 9.1 Hz, 1H), 7.38 (d, *J* = 8.3 Hz, 2H), 7.34–7.25 (m, 5H), 7.15 (dd, *J* = 9.2, 1.4 Hz, 1H), 7.07 (d, *J* = 8.1 Hz, 2H), 4.86 (s, 2H), 2.36 (d, *J* = 6.8 Hz, 6H). ^13^C{1H} NMR (75 MHz, CDCl_3_) δ 147.19, 145.32, 145.04, 134.35, 133.19, 129.85, 129.15, 128.33, 128.27, 128.20, 128.04, 122.69, 122.52, 116.86, 108.00, 52.81, 21.70, 18.55.

7-Chloro-2-phenyl-3-(tosylmethyl)imidazo[1,2-α]pyridine (**3sd**) [29]. White solid, 190 mg, 80% yield, ethyl acetate/*n*-hexane (1:1). ^1^H NMR (300 MHz, CDCl_3_) δ 8.22 (d, *J* = 7.3 Hz, 1H), 7.74 (d, *J* = 1.8 Hz, 1H), 7.26 (d, *J* = 8.3 Hz, 2H), 7.18 (s, 5H), 6.98 (d, *J* = 8.0 Hz, 2H), 6.89 (dd, *J* = 7.3, 2.0 Hz, 1H), 4.74 (s, 2H), 2.25 (s, 3H). ^13^C{1H} NMR (75 MHz, CDCl_3_) δ 147.76, 145.92, 145.44, 133.98, 132.33, 129.85, 128.37, 128.13, 128.03, 125.44, 120.06, 119.50, 116.76, 108.83, 52.35, 21.65.

7-Bromo-2-phenyl-3-(tosylmethyl)imidazo[1,2-α]pyridine (**3se**). White solid, 211 mg, 80% yield, ethyl acetate/*n*-hexane (1:1). mp. 231–132 °C. ^1^H NMR (300 MHz, CDCl_3_) δ 8.29 (d, *J* = 7.3 Hz, 1H), 7.56 (d, *J* = 1.6 Hz, 1H), 7.29–7.25 (m, 2H), 7.19 (s, 5H), 6.99 (d, *J* = 8.1 Hz, 2H), 6.79 (dd, *J* = 7.4, 2.1 Hz, 1H), 4.75 (s, 2H), 2.26 (s, 3H). ^13^C{1H} NMR (75 MHz, CDCl_3_) δ 147.98, 145.65, 145.45, 134.01, 132.63, 132.41, 129.86, 128.37, 128.12, 128.05, 125.50, 116.17, 114.46, 108.75, 52.39, 21.64. HRMS (ESI) m/z: [M + H]+ Calcd for C_21_H_18_BrN_2_O_2_S^+^ 441.0267, Found 441.0267.

2-(Furan-2-yl)-3-(tosylmethyl)imidazo[1,2-α]pyridine (**3ta**) [29]. White solid, 173 mg, 82% yield, ethyl acetate/*n*-hexane (1:1). ^1^H NMR (300 MHz, CDCl_3_) δ 8.38 (d, *J* = 7.0 Hz, 1H), 7.60 (d, *J* = 9.2 Hz, 1H), 7.37 (d, *J* = 7.9 Hz, 2H), 7.29 (t, *J* = 8.2 Hz, 1H), 7.12 (s, 1H), 7.05 (d, *J* = 8.0 Hz, 2H), 6.92 (d, *J* = 7.0 Hz, 1H), 6.67 (d, *J* = 3.0 Hz, 1H), 6.35–6.24 (m, 1H), 5.07 (s, 2H), 2.26 (s, 3H). ^13^C{1H} NMR (75 MHz, CDCl_3_) δ 148.92, 146.24, 145.13, 142.10, 137.51, 133.82, 129.22, 128.31, 126.45, 124.67, 117.09, 112.98, 111.08, 108.44, 107.95, 52.55, 21.50.

2-(*t*-Butyl)-3-(tosylmethyl)imidazo[1,2-α]pyridine (**3tb**).^12^ White solid, 176 mg, 86% yield, ethyl acetate/*n*-hexane (1:1). ^1^H NMR (300 MHz, CDCl_3_) δ 8.21 (d, *J* = 6.9 Hz, 1H), 7.60 (d, *J* = 8.2 Hz, 3H), 7.28 (d, *J* = 8.0 Hz, 2H), 7.20 (t, *J* = 8.1 Hz, 1H), 6.76 (t, *J* = 6.9 Hz, 1H), 4.89 (s, 2H), 2.41 (s, 3H), 1.30 (d, *J* = 2.1 Hz, 9H). ^13^C{1H} NMR (75 MHz, CDCl_3_) δ 155.80, 145.49, 144.90, 135.55, 130.04, 128.52, 125.27, 124.20, 116.92, 112.12, 106.12, 53.60, 34.18, 30.84, 21.68.

6-Phenyl-5-tosyl-2,3-dihydroimidazo[2,1-b]thiazole (**3ua**) [29]. White solid, 171 mg, 80% yield, ethyl acetate/*n*-hexane (1:1). ^1^H NMR (300 MHz, CDCl_3_) δ 7.40 (d, *J* = 7.8 Hz, 1H), 7.06 (d, *J* = 8.1 Hz, 1H), 4.45 (s, 1H), 4.29 (t, *J* = 7.4 Hz, 1H), 3.78 (t, *J* = 7.3 Hz, 1H). ^13^C{1H} NMR (75 MHz, CDCl_3_) δ 151.08, 147.55, 145.20, 133.87, 133.20, 129.67, 128.06, 128.03, 127.10, 126.68, 113.96, 53.18, 46.11, 34.77, 21.55.

6-Phenyl-5-tosylimidazo[2,1-b]thiazole (**3ub**) [29]. White solid, 165 mg, 78% yield, ethyl acetate/*n*-hexane (1:1). ^1^H NMR (300 MHz, CDCl_3_) δ 7.67 (d, *J* = 4.5 Hz, 1H), 7.43 (d, *J* = 8.0 Hz, 2H), 7.27–7.15 (m, 5H), 7.12 (d, *J* = 7.9 Hz, 2H), 6.86 (d, *J* = 4.4 Hz, 1H), 4.69 (s, 2H), 2.34 (s, 3H). ^13^C{1H} NMR (75 MHz, CDCl_3_) δ 150.77, 148.42, 145.37, 134.00, 133.01, 129.83, 128.27, 128.16, 127.78, 127.49, 119.07, 112.72, 109.77, 77.58, 77.16, 76.74, 53.45, 21.62.

2-(Thiophen-2-yl)-3-(tosylmethyl)imidazo[1,2-α] pyrimidine (**3uc**) [29]. White solid, 177 mg, 80% yield, ethyl acetate/*n*-hexane (1:1). ^1^H NMR (300 MHz, CDCl_3_) δ 8.72 (d, *J* = 6.7 Hz, 1H), 8.60 (s, 1H), 7.48 (d, *J* = 7.9 Hz, 2H), 7.29 (d, *J* = 5.2 Hz, 1H), 7.15 (d, *J* = 7.9 Hz, 2H), 7.07 (d, *J* = 3.2 Hz, 1H), 6.94 (s, 2H), 4.91 (s, 2H), 2.33 (s, 3H). ^13^C{1H} NMR (75 MHz, CDCl_3_) δ 151.32, 148.80, 145.86, 142.92, 135.04, 134.03, 132.76, 130.04, 128.37, 127.58, 127.18, 126.24, 109.20, 106.21, 52.67, 21.71.

3-Methyl-1-phenyl-4-(tosylmethyl)-1H-pyrazol-5-amine (**3ud**) [34]. Yellow solid, 167 mg, yield 82%, ethyl acetate/*n*-hexane (1:1). ^1^H NMR (300 MHz, CDCl_3_) δ 7.65 (d, *J* = 8.3 Hz, 2H), 7.50–7.42 (m, 4H), 7.37–7.28 (m, 3H), 4.40 (br s, 2H), 4.12 (s, 2H), 2.43 (s, 3H), 1.62 (s, 3H). ^13^C{1H} NMR (75 MHz, CDCl_3_) δ 148.75, 145.56, 144.99, 138.05, 134.66, 129.78, 129.58, 128.54, 127.61, 124.01, 90.17, 53.08, 21.65, 11.24.

## 4. Conclusions

In conclusion, we have developed a new sulfonylmethylation approach for imidazo[1,2-α]pyridines with DMA and sodium sulfinates affording diverse 3-(sulfonylmethyl)imidazo[1,2-α]pyridines in good yields with impressive simplicity. Free radical trapping and isotope labeling experiments showed that an oxidation-addition pathway might be included, and DMA provided carbon in this reaction.

## Figures and Tables

**Table 1:** Optimization of reaction conditions *^a^*.

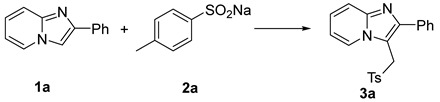				
**Entry**	**Catalyst/mol%**	**Oxidant/Equiv**	**Solvent**	**Yield/% *^b^***
1	-	-	DMA:H_2_O/2:1	0
2	-	K_2_S_2_O_8_/2.5	DMA:H_2_O/2:1	5
3	FeCl_3_/20	-	DMA:H_2_O/2:1	0
4	CuI/20	K_2_S_2_O_8_/2.5	DMA:H_2_O/2:1	18
5	Cu(acac)_2_/20	K_2_S_2_O_8_/2.5	DMA:H_2_O/2:1	18
6	FeCl_3_/20	K_2_S_2_O_8_/2.5	DMA:H_2_O/2:1	73
7	FeCl_2_/20	K_2_S_2_O_8_/2.5	DMA:H_2_O/2:1	67
8	Fe_3_O_4_/20	K_2_S_2_O_8_/2.5	DMA:H_2_O/2:1	50
9	V(acac)_2_/20	K_2_S_2_O_8_/2.5	DMA:H_2_O/2:1	63
10	AgNO_3_/20	K_2_S_2_O_8_/2.5	DMA:H_2_O/2:1	6
11	FeCl_3_/5	K_2_S_2_O_8_/2.5	DMA:H_2_O/2:1	41
12	FeCl_3_/10	K_2_S_2_O_8_/2.5	DMA:H_2_O/2:1	73
13	FeCl_3_/30	K_2_S_2_O_8_/2.5	DMA:H_2_O/2:1	70
14	FeCl_3_/10	H_2_O_2_/2.5	DMA:H_2_O/2:1	0
15	FeCl_3_/10	TBHP/2.5	DMA:H_2_O/2:1	0
16	FeCl_3_/10	O_2_/air	DMA:H_2_O/2:1	0
17	FeCl_3_/10	I_2_O_5_/2.5	DMA:H_2_O/2:1	0
18	FeCl_3_/10	K_2_S_2_O_8_/1.0	DMA:H_2_O/2:1	57
19	FeCl_3_/10	K_2_S_2_O_8_/1.5	DMA:H_2_O/2:1	64
20	FeCl_3_/10	K_2_S_2_O_8_/3.5	DMA:H_2_O/2:1	73
21	FeCl_3_/10	K_2_S_2_O_8_/2.5	DMA/10 eq	0
22	FeCl_3_/10	K_2_S_2_O_8_/2.5	DMA/20 eq	0
23	FeCl_3_/10	K_2_S_2_O_8_/2.5	DMA/50 eq	43
24	FeCl_3_/10	K_2_S_2_O_8_/2.5	DMA:H_2_O/1:1	43
25	FeCl_3_/10	K_2_S_2_O_8_/2.5	DMA:H_2_O/5:1	70
26 *^c^*	FeCl_3_/10	K_2_S_2_O_8_/2.5	DMA:H_2_O/2:1	80
27 *^d^*	FeCl_3_/10	K_2_S_2_O_8_/2.5	DMA:H_2_O/2:1	90
28 *^e^*	FeCl_3_/10	K_2_S_2_O_8_/2.5	DMA:H_2_O/2:1	90
29 *^d^*^,*f*^	FeCl_3_/10	K_2_S_2_O_8_/2.5	DMA:H_2_O/2:1	60
30 *^d^*^,*g*^	FeCl_3_/10	K_2_S_2_O_8_/2.5	DMA:H_2_O/2:1	70
31 *^d^*^,*h*^	FeCl_3_/10	K_2_S_2_O_8_/2.5	DMA:H_2_O/2:1	93
32 *^d^*^,*i*^	FeCl_3_/10	K_2_S_2_O_8_/2.5	DMA:H_2_O/2:1	93
33 *^d^*^,*h*^	FeCl_3_/10	K_2_S_2_O_8_/2.5	DMF:H_2_O/2:1	33
34 *^d^*^,*h*^	FeCl_3_/10	K_2_S_2_O_8_/2.5	DMSO:H_2_O/2:1	0
35 *^d^*^,*h*^	FeCl_3_/10	K_2_S_2_O_8_/2.5	TMEDA:H_2_O/2:1	0

*^a^* Reaction conditions: **1a** (0.2 mmol), **2a** (0.24 mmol), catalyst, oxidant, solvent (2 mL), 110 °C, 4 h; *^b^* LC-MS; ^c^ **2a** (1.5 eq); *^d^* **2a** (2.0 eq); *^e^* **2a** (2.5 eq); *^f^* 90 °C; *^g^* 100 °C; *^h^* 120 °C; *^i^* 130 °C.

**Table 2:** Substrate scope of sodium sulfinates *^a^*^,*b*^.

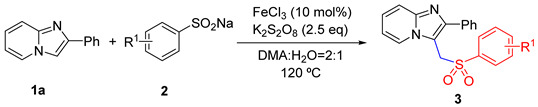
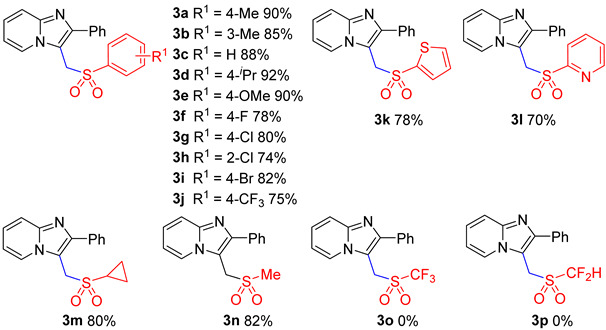

*^a^* Reaction conditions: **1a** (0.6 mmol), **2a** (1.2 mmol), FeCl_3_ (10 mol%), K_2_S_2_O_8_ (2.5 eq), DMA:H_2_O = 2:1, 120 °C, 4 h; *^b^* isolated yields.

**Table 3:** Substrate scope of imidazo[1,2-α]pyridines *^a^*^,*b*^.

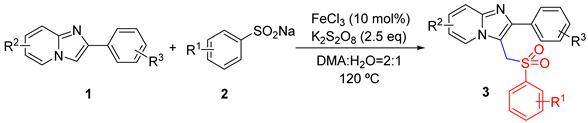
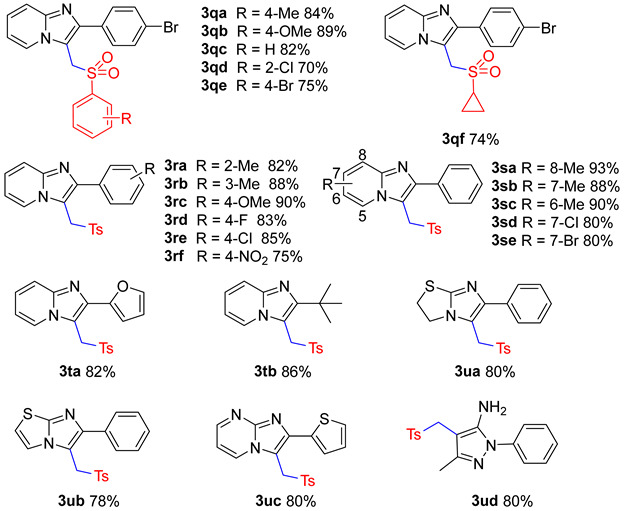

*^a^* Reaction conditions: **1a** (0.6 mmol), **2a** (1.2 mmol), FeCl_3_ (10 mol%), K_2_S_2_O_8_ (2.5 eq), DMA:H_2_O = 2:1, 120 °C, 8 h; *^b^* isolated yields.

## Data Availability

The original contributions presented in the study are included in the article/Supplementary Materials, further inquiries can be directed to the corresponding author/s.

## Supplementary Materials

The following supporting information can be downloaded at: https://www.mdpi.com/article/10.3390/molecules29133196/s1, References [29,34] are cited in the supplementary materials.
