# Supplementary material for: Iron-Catalyzed Sulfonylmethylation of Imidazo[1,2-α]pyridines with N,N-Dimethylacetamide and Sodium Sulfinates
Source: Molecules. 2024 Jul 5;29(13):3196. doi: 10.3390/molecules29133196 (PMC11243443; doi:10.3390/molecules29133196)
Supplement: Supplementary file 1 [file molecules-29-03196-s001.zip › molecules-3035476-supplementary.pdf]

Supporting Information

Iron-Catalyzed Sulfonylmethylation of Imidazo[1,2-α]pyridines with DMA and Sodium Sulfinates

Shengnan Sun, Hexia Ye, Haibo Liu*, Junchen Li*, Xiaojing Bi*

State Key Laboratory of NBC Protection for Civilian, Beijing, 102205, P. R. China

*E-mail: xiaojingbimail@yeah.net

Copies of ^{1}H NMR ^{13}C NMR and ^{19}F NMR spectra

^{1}H NMR (300 MHz, $CDCl_3$) of **3a**

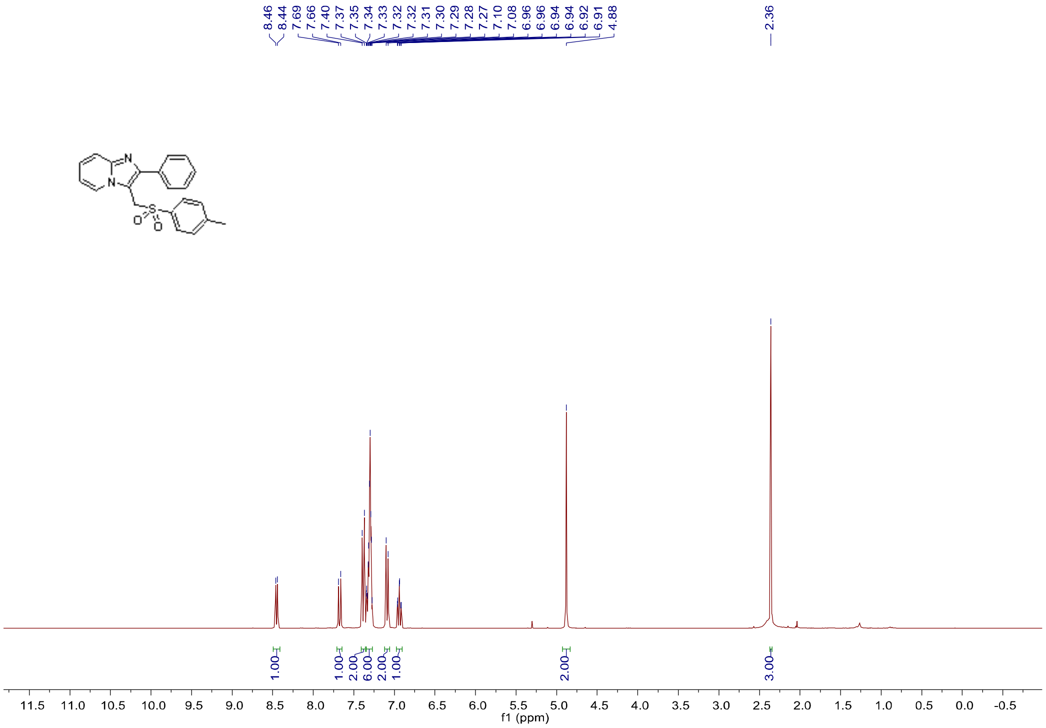

$^{13}C\{^{1}H\}$ (75 MHz, $CDCl_3$) NMR of **3a**

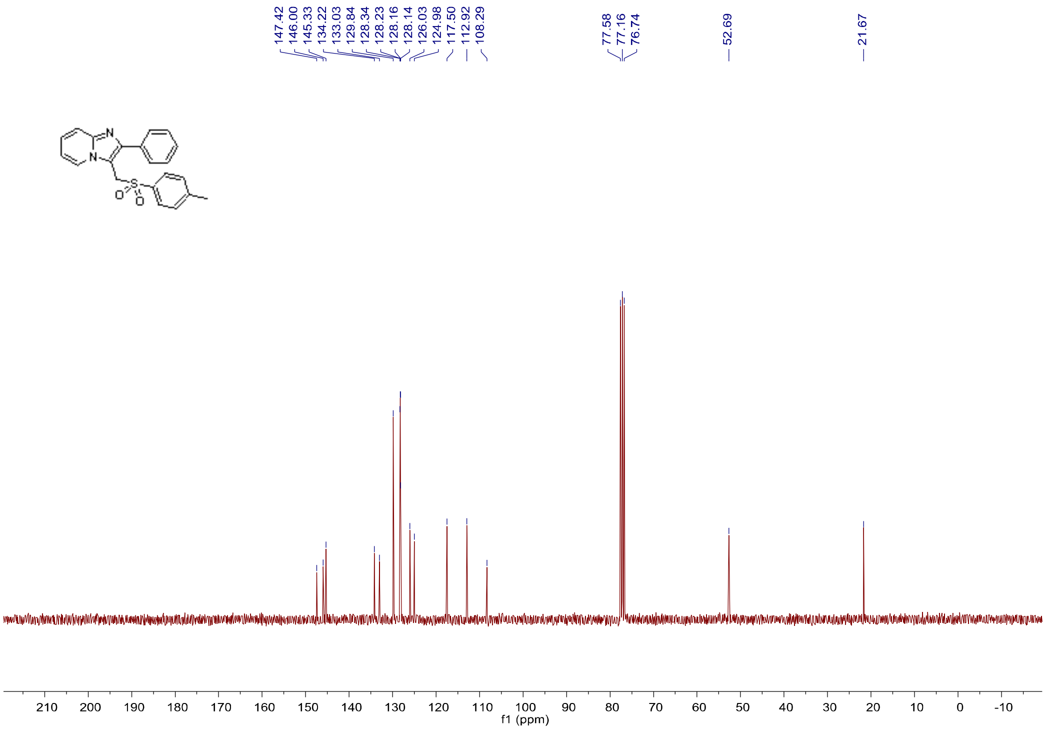

^{1}H NMR (300 MHz, $CDCl_3$) of **3b**

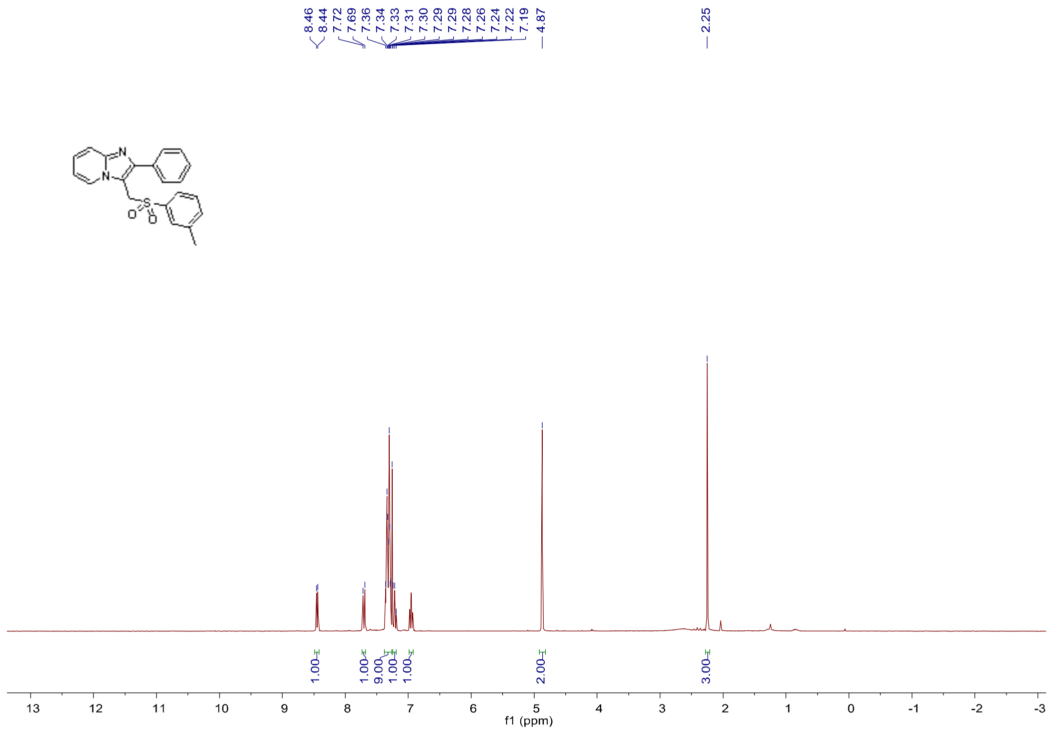

^{13}C{^{1}H} (75 MHz, $CDCl_3$) NMR of **3b**

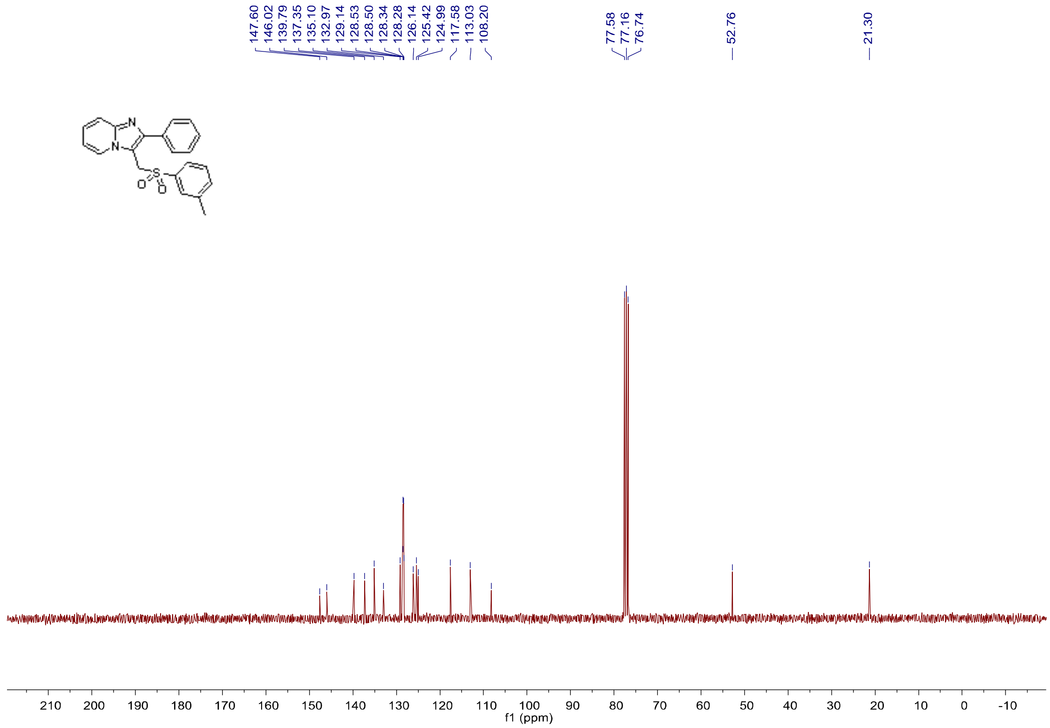

^{1}H NMR (300 MHz, $CDCl_3$) of **3c**

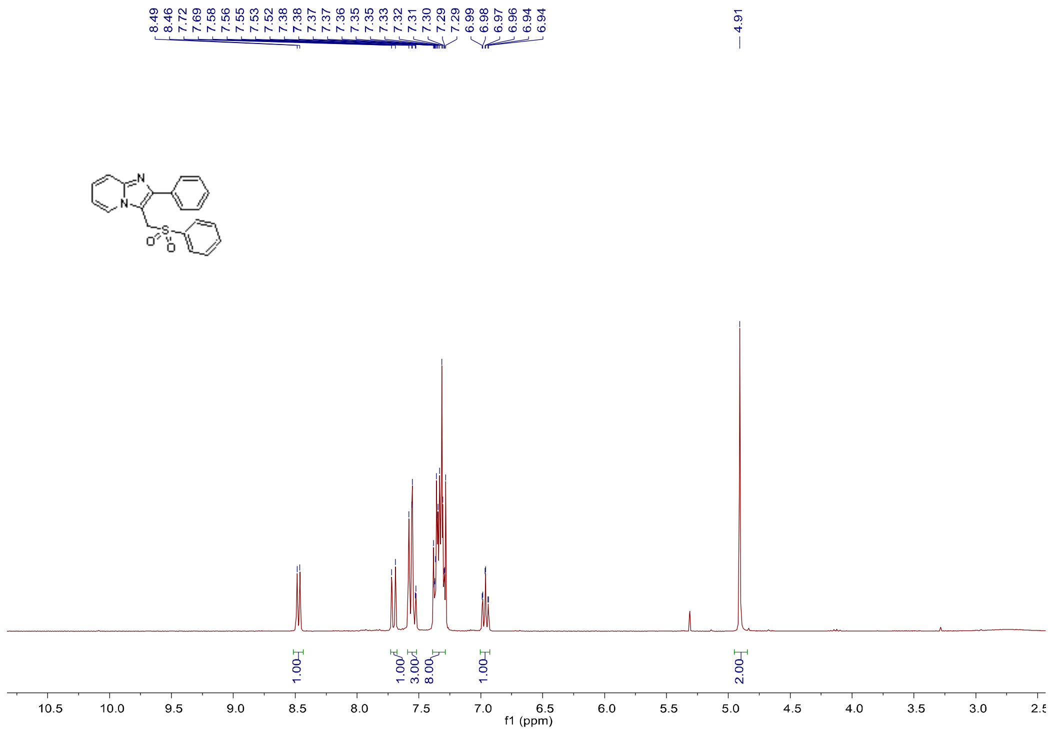

^{13}C{^{1}H} (75 MHz, $CDCl_3$) NMR of **3c**

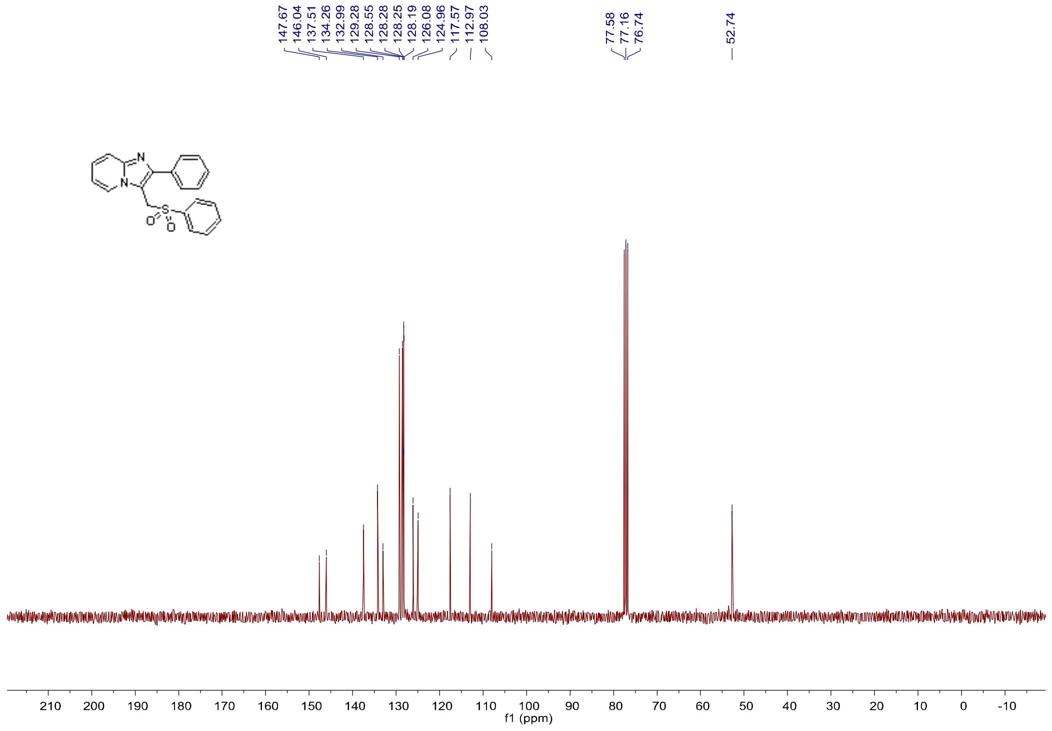

^{1}H NMR (300 MHz, $CDCl_3$) of **3d**

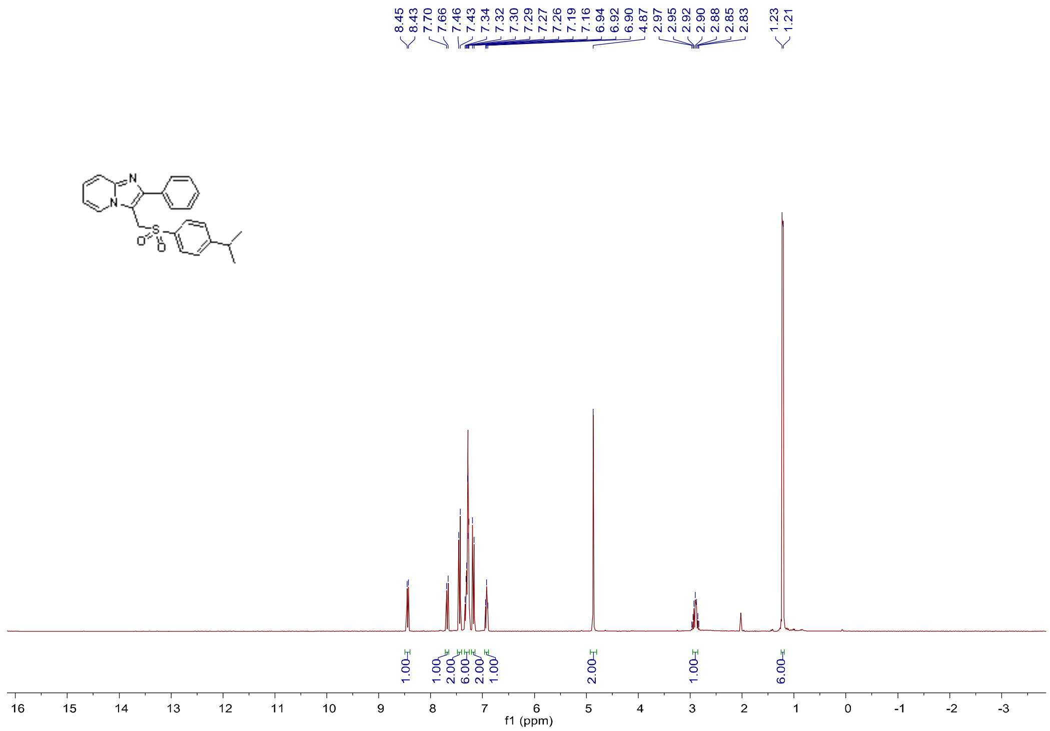

^{13}C{^{1}H} (75 MHz, $CDCl_3$) NMR of **3d**

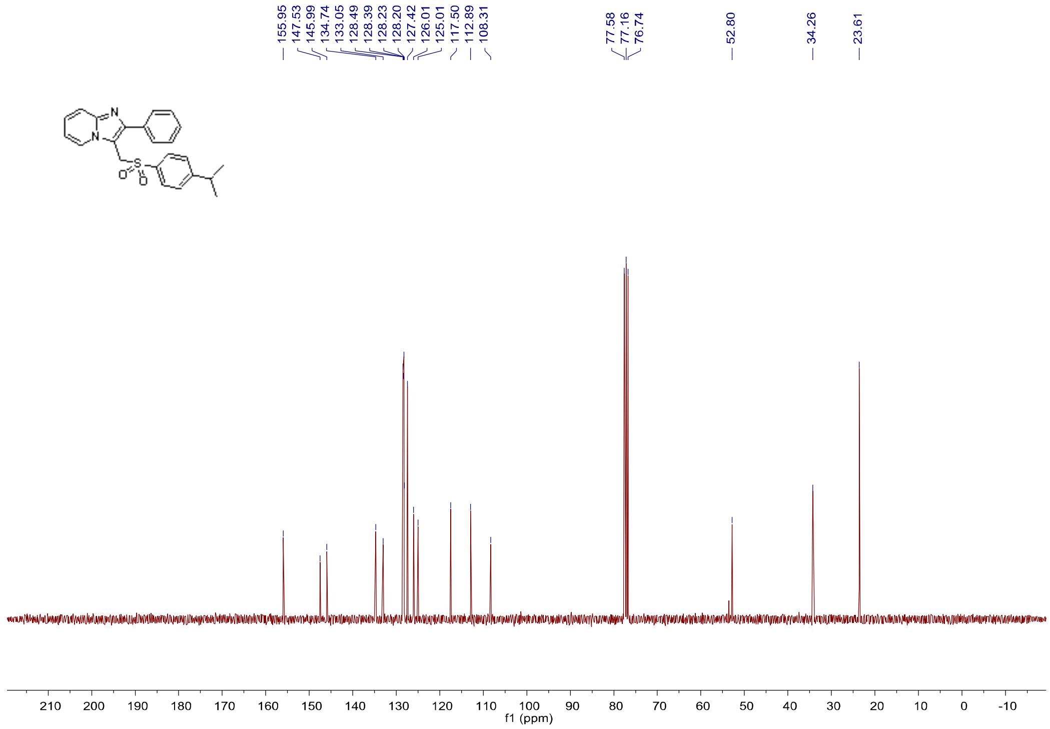

^{1}H NMR (300 MHz, CDCl$_3$) of **3e**

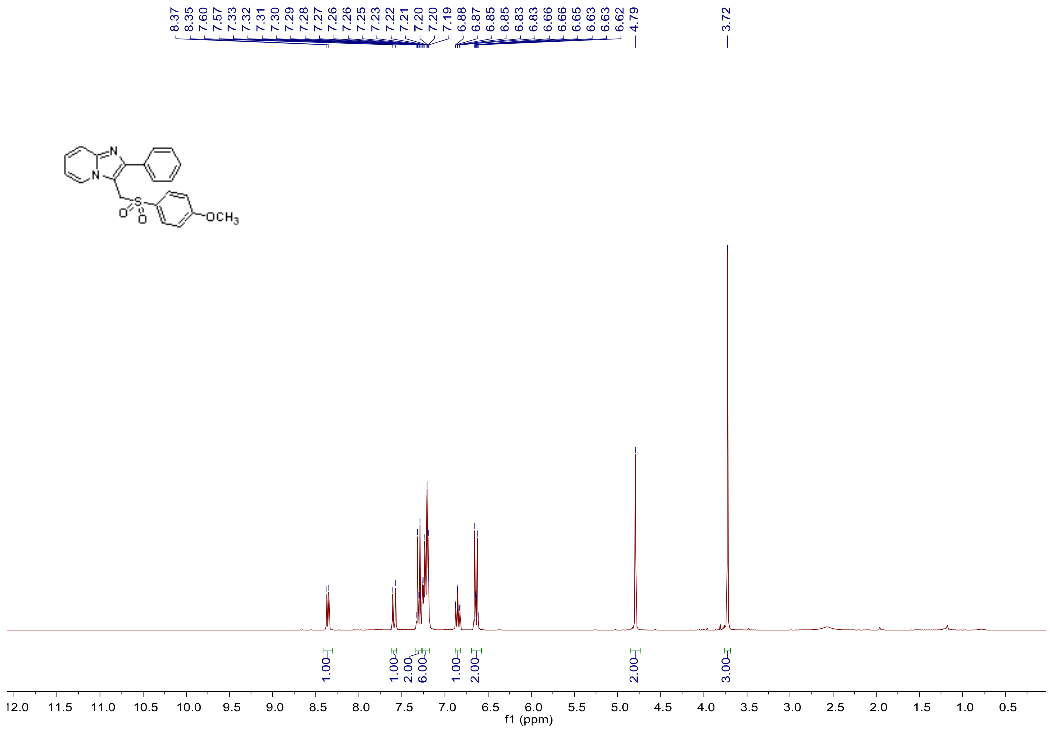

^{13}C{^{1}H} (75 MHz, CDCl$_3$) NMR of **3e**

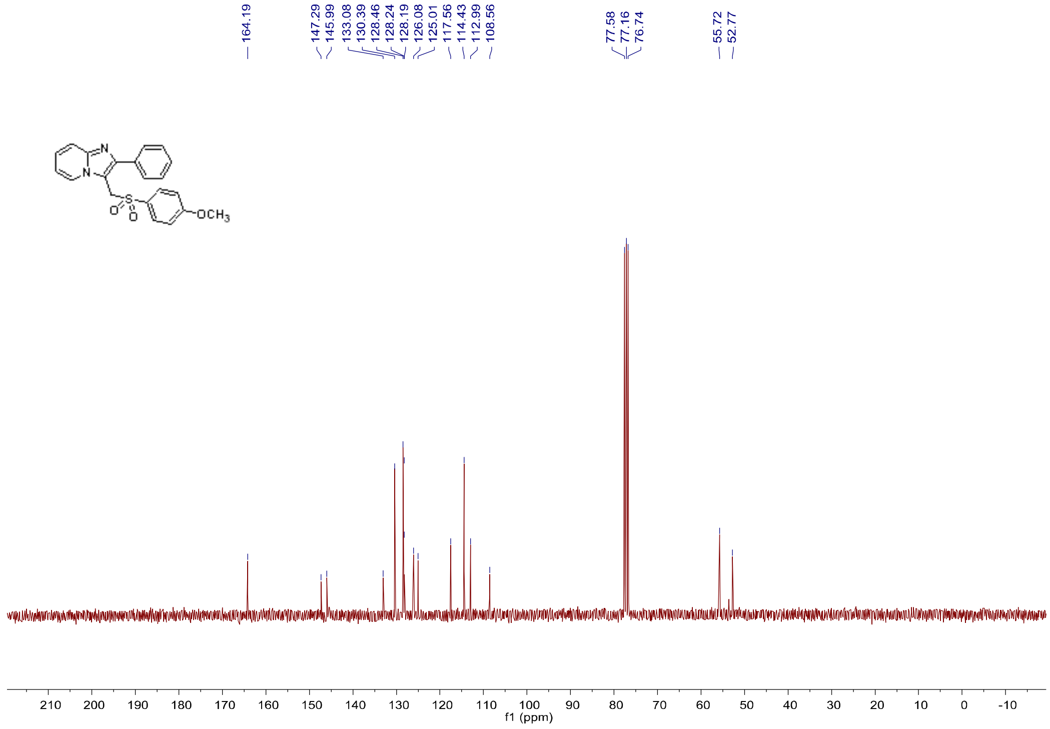

^{1}H NMR (300 MHz, $CDCl_3$) of **3f**

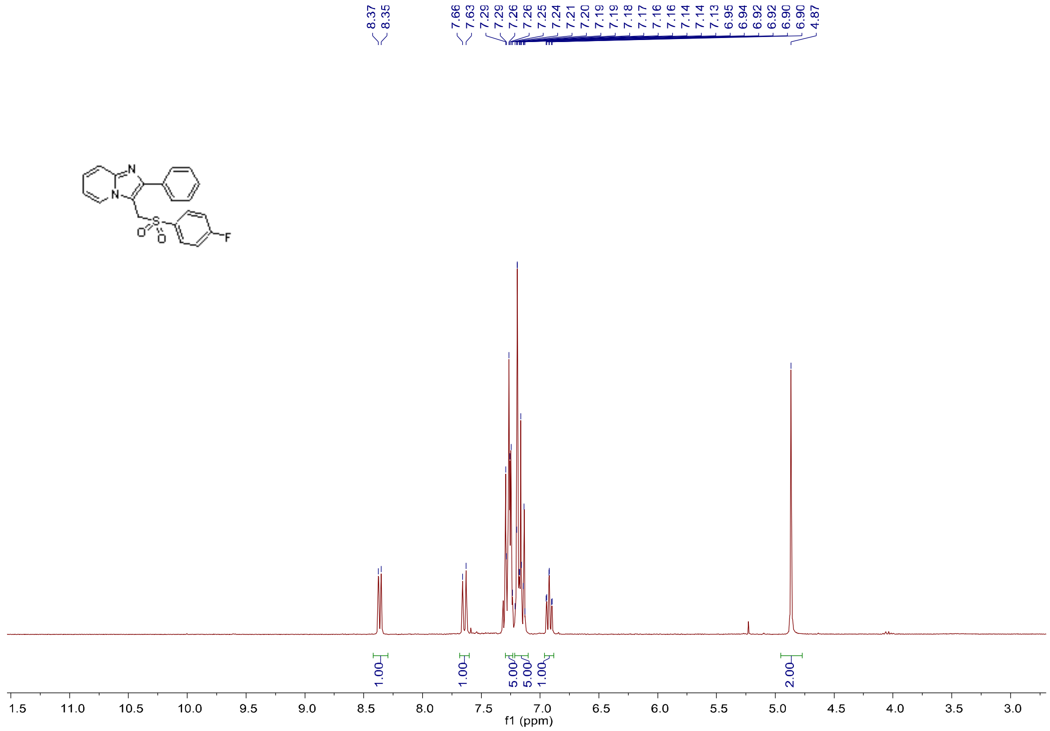

^{13}C{^{1}H} (75 MHz, $CDCl_3$) NMR of **3f**

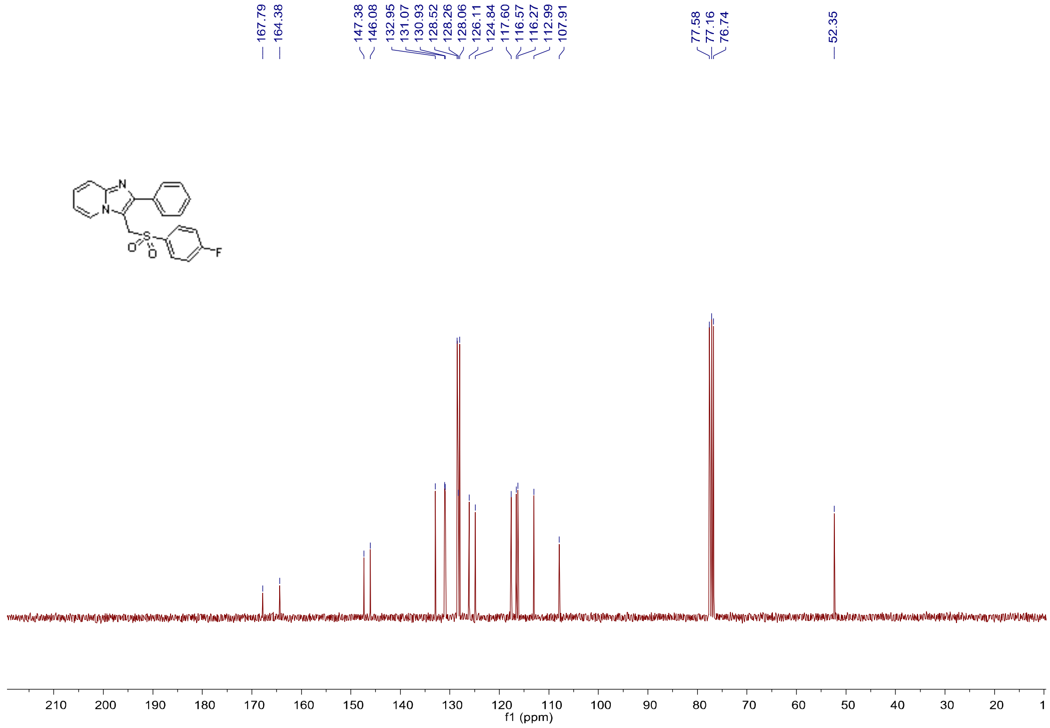

^{19}F NMR (282 MHz, CDCl$_3$) of **3f**

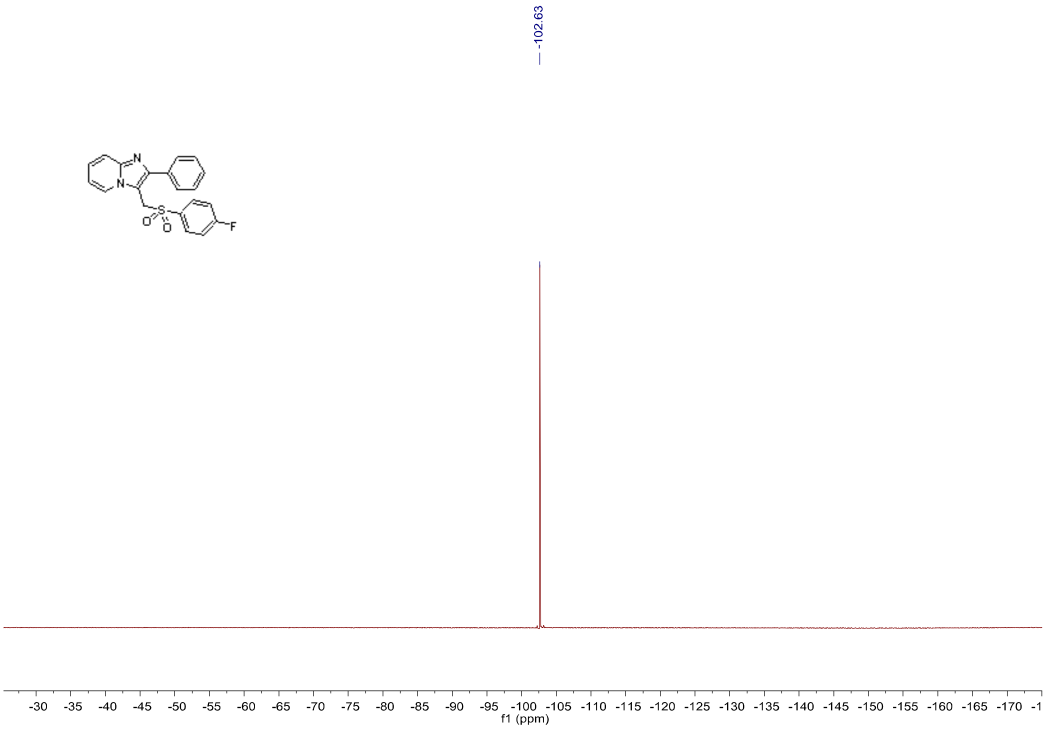

^{1}H NMR (300 MHz, CDCl$_3$) of **3g**

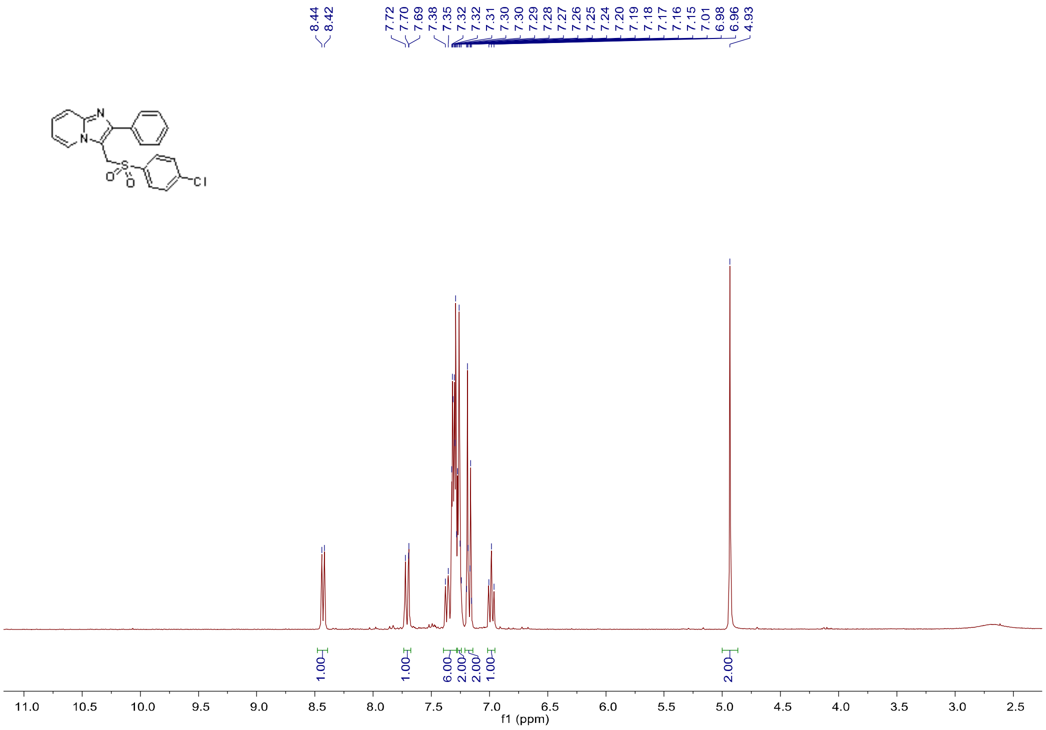

$^{13}C\{^{1}H\}$ (75 MHz, $CDCl_3$) NMR of **3g**

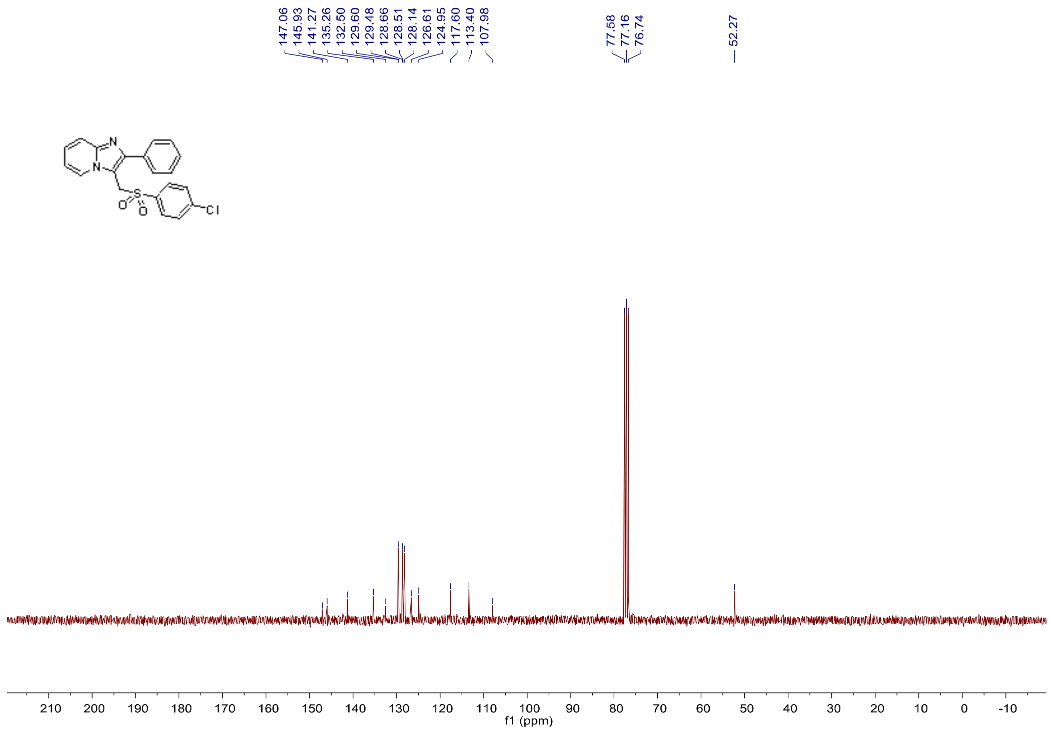

^{1}H NMR (300 MHz, $CDCl_3$) of **3h**

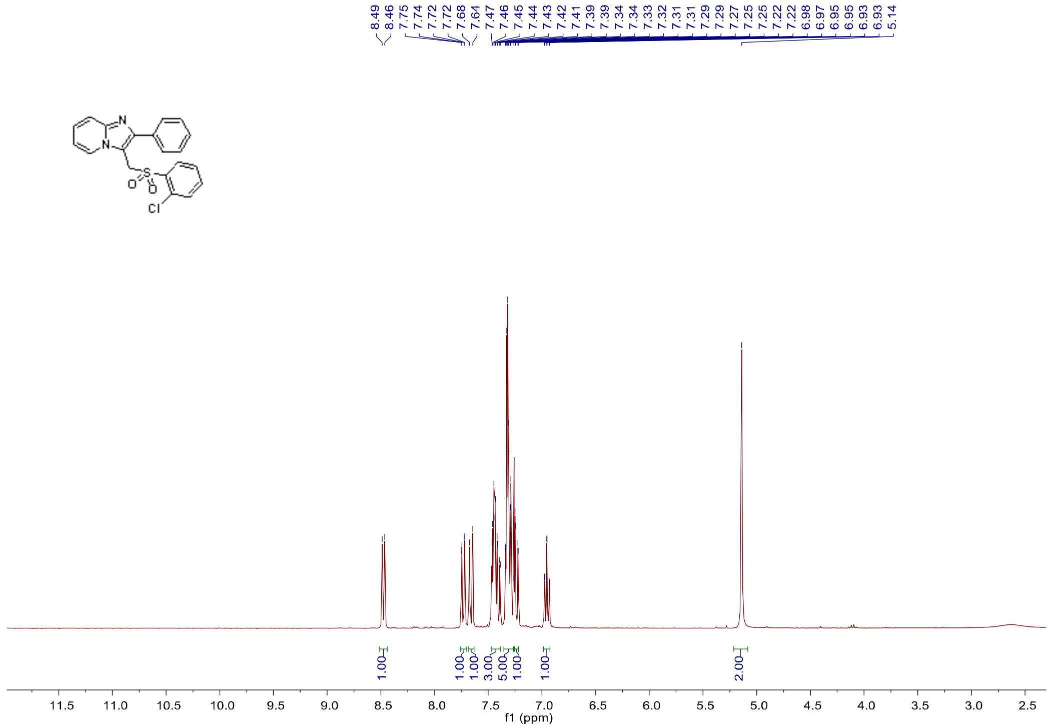

$^{13}C\{^{1}H\}$ (75 MHz, $CDCl_3$) NMR of **3h**

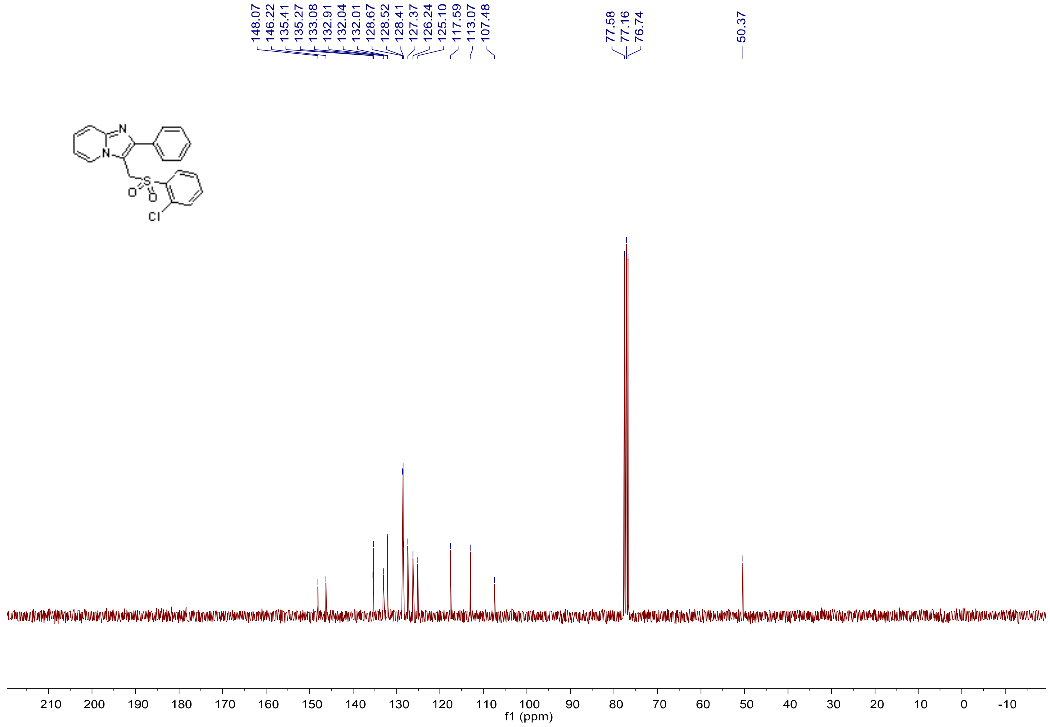

^{1}H NMR (300 MHz, $CDCl_3$) of **3i**

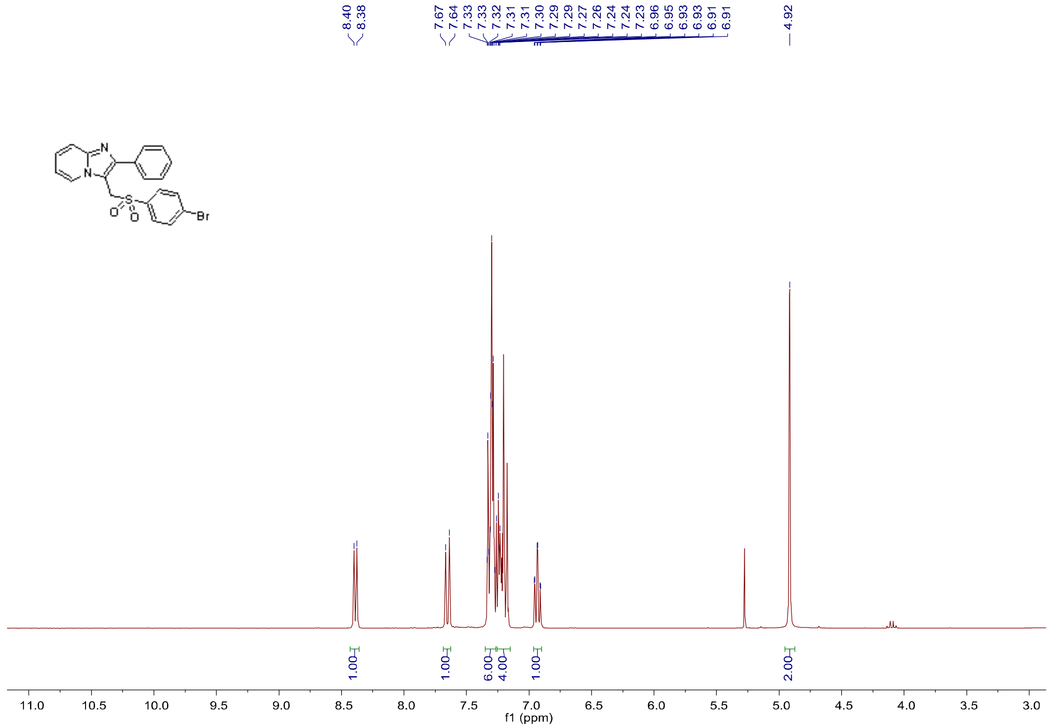

$^{13}C\{^{1}H\}$ (75 MHz, $CDCl_3$) NMR of **3i**

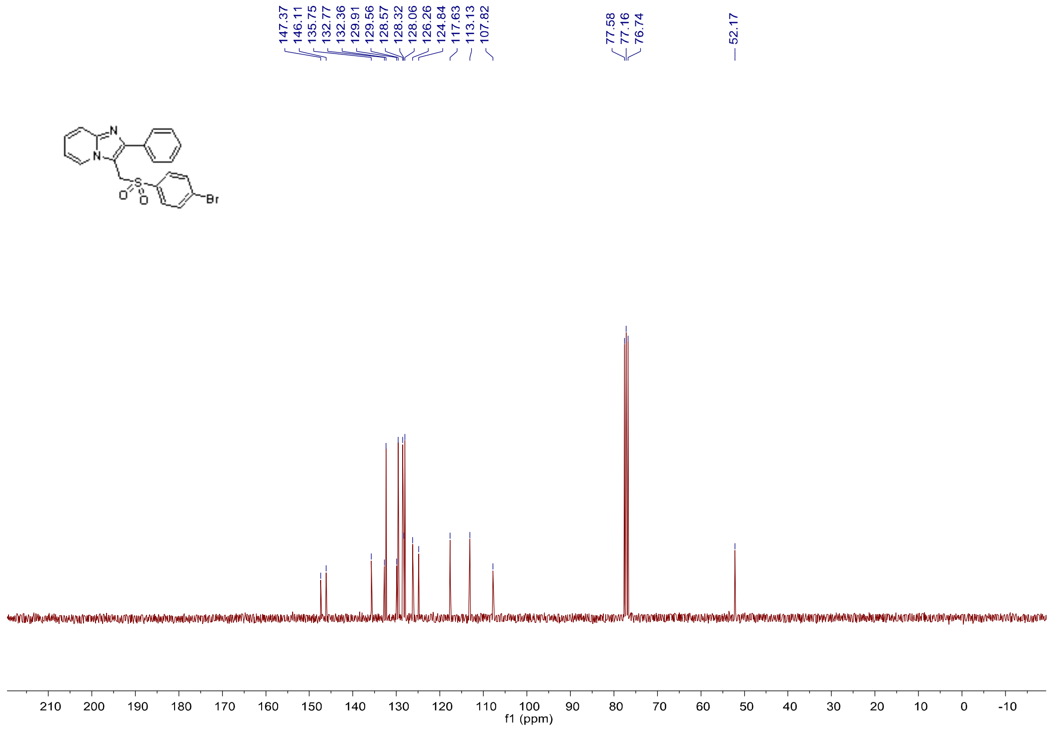

^{1}H NMR (300 MHz, $CDCl_3$) of **3j**

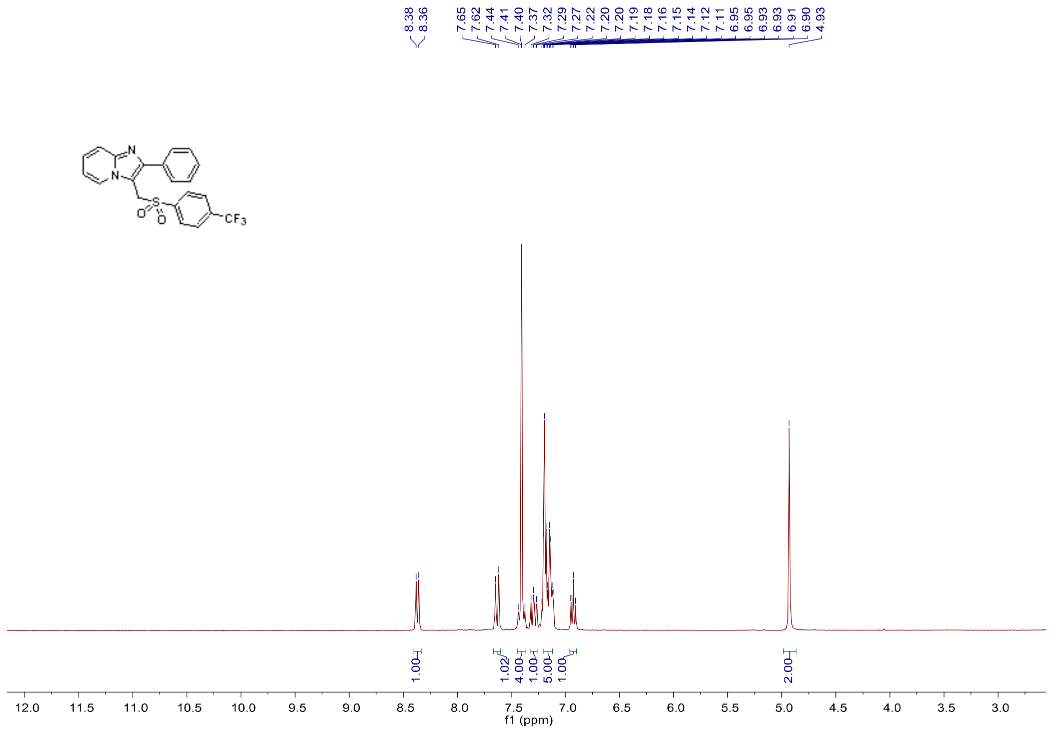

$^{13}C\{^{1}H\}$ (75 MHz, $CDCl_3$) NMR of **3j**

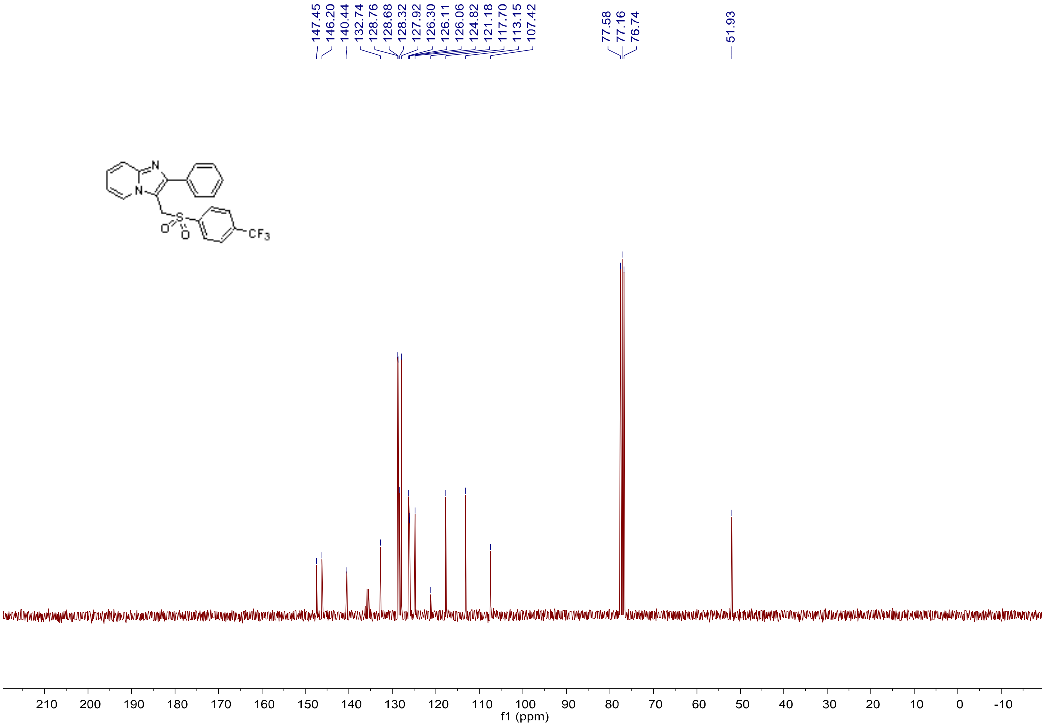

^{1}H NMR (300 MHz, $CDCl_3$) of **3k**

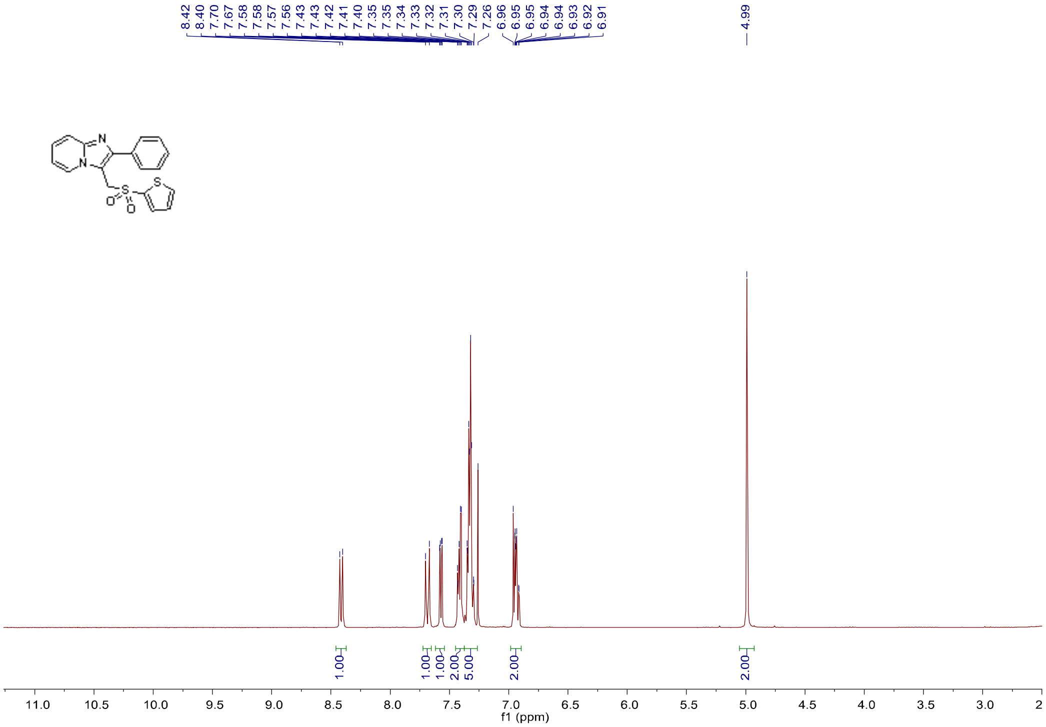

$^{13}C\{^{1}H\}$ (75 MHz, $CDCl_3$) NMR of **3k**

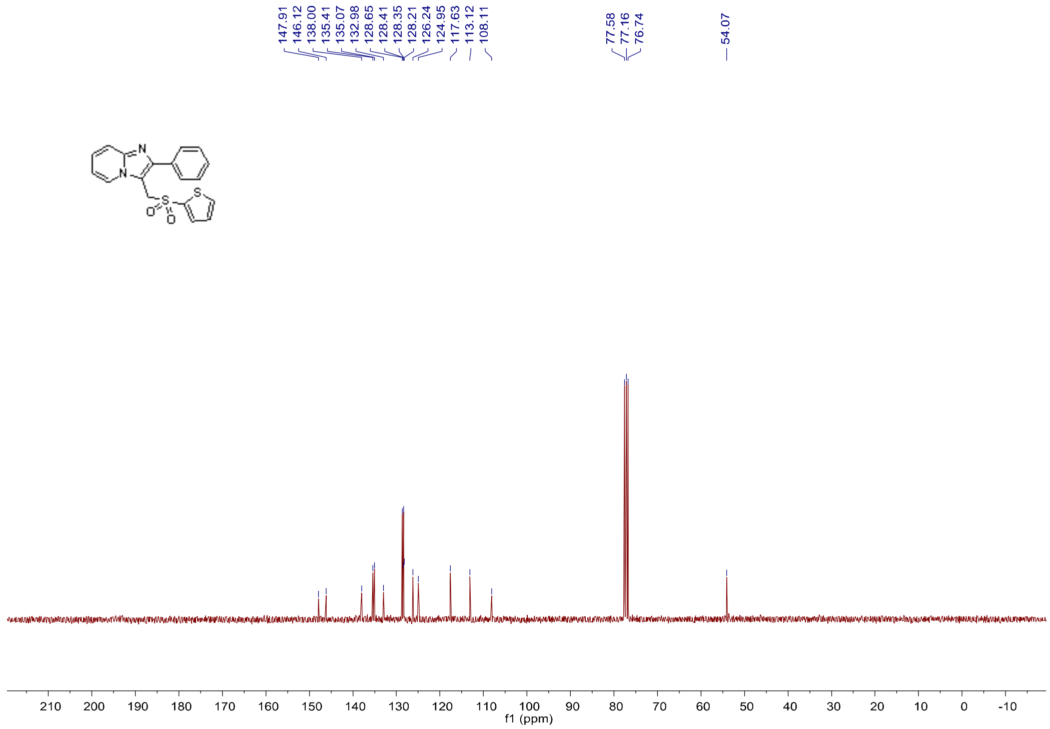

^{1}H NMR (300 MHz, $CDCl_3$) of **3l**

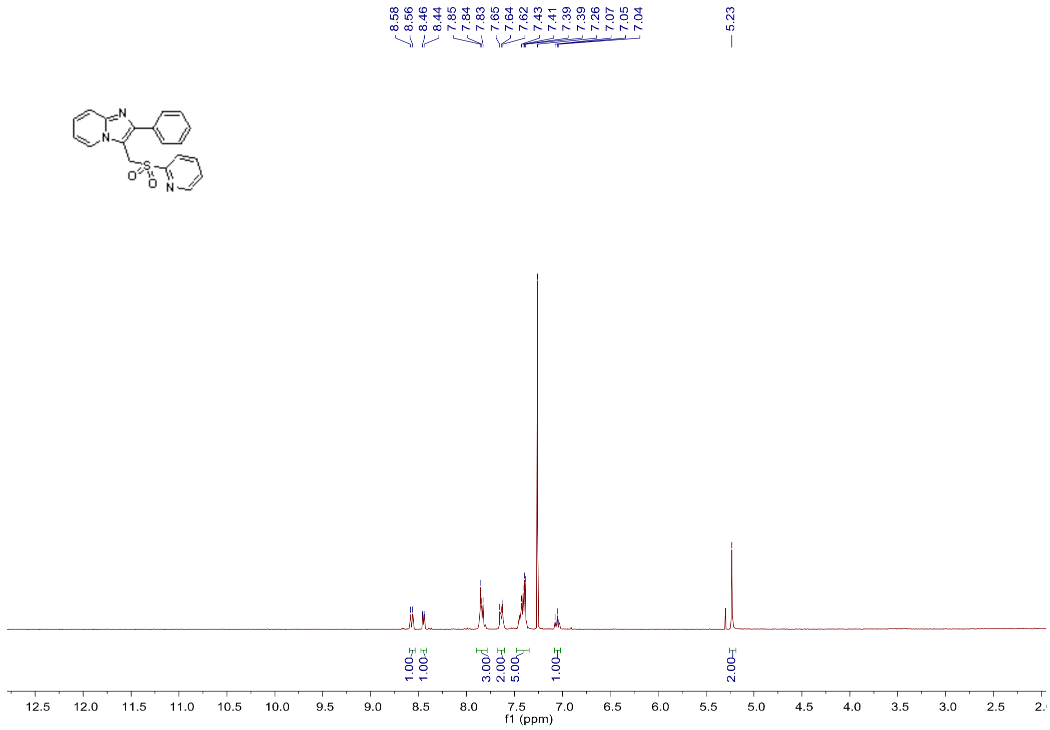

$^{13}C\{^{1}H\}$ (75 MHz, $CDCl_3$) NMR of **3l**

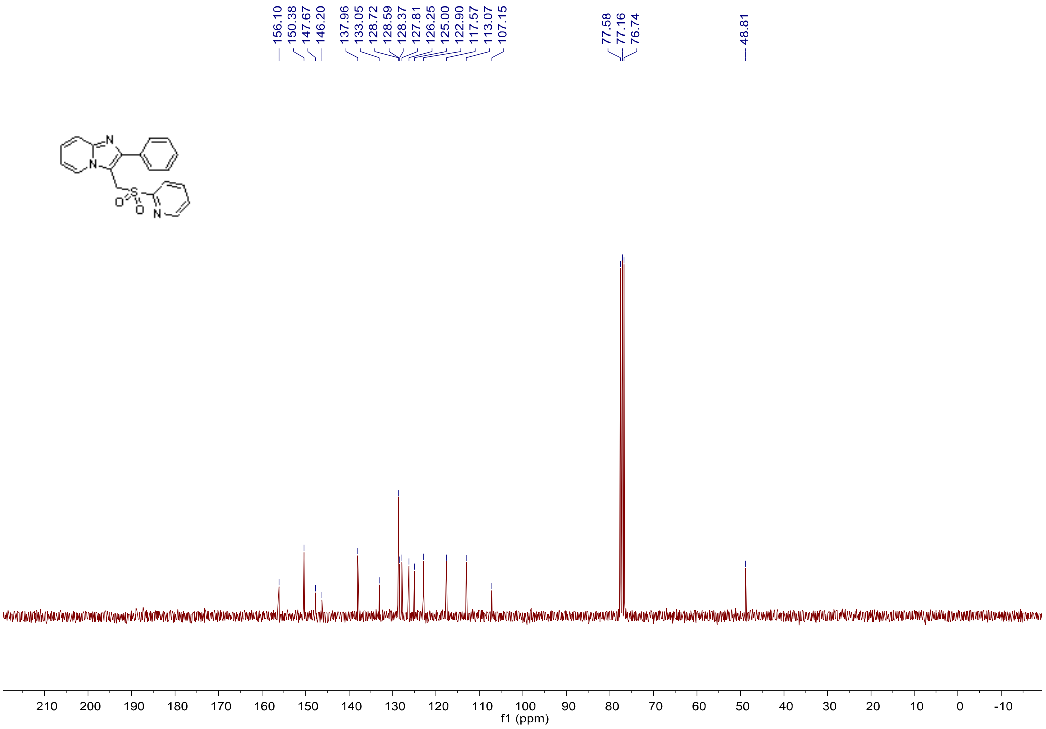

^{1}H NMR (300 MHz, $CDCl_3$) of **3m**

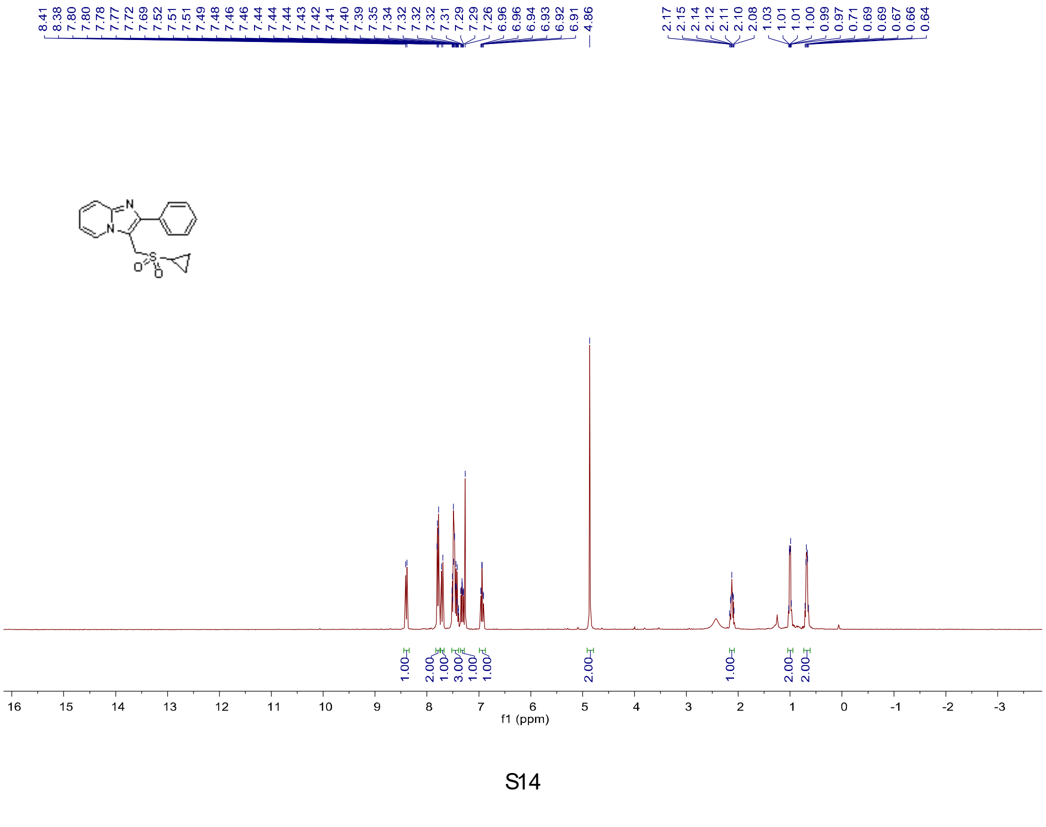

$^{13}C\{^{1}H\}$ (75 MHz, $CDCl_3$) NMR of **3m**

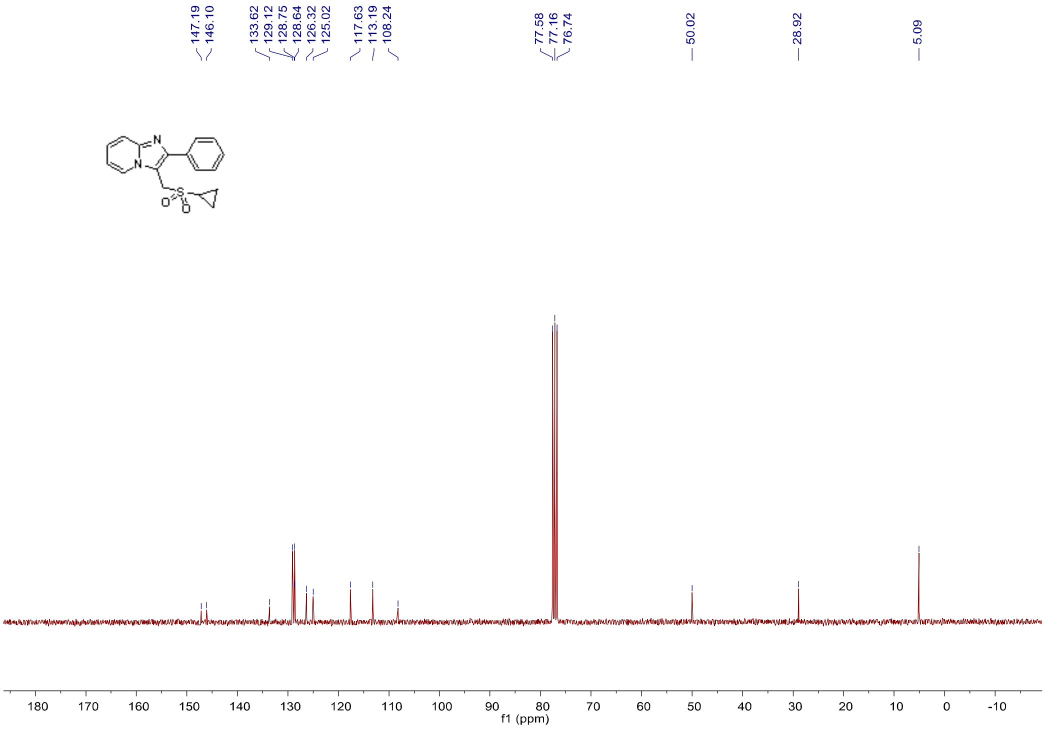

^{1}H NMR (300 MHz, $CDCl_3$) of **3n**

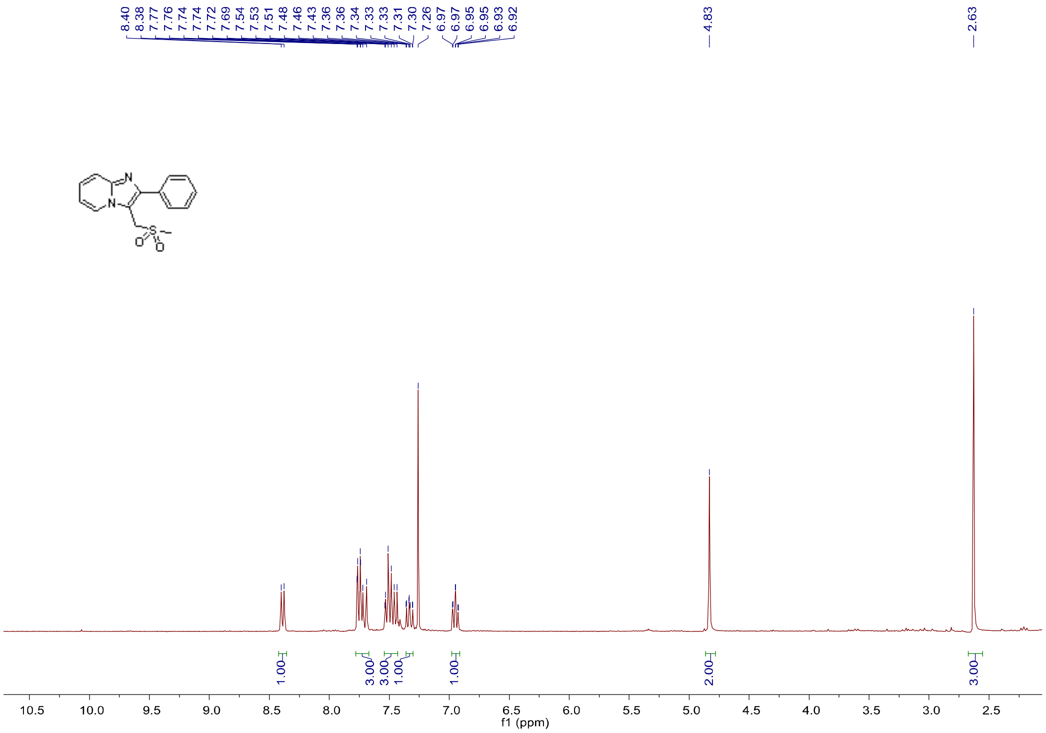

$^{13}C\{^{1}H\}$ (75 MHz, $CDCl_3$) NMR of **3n**

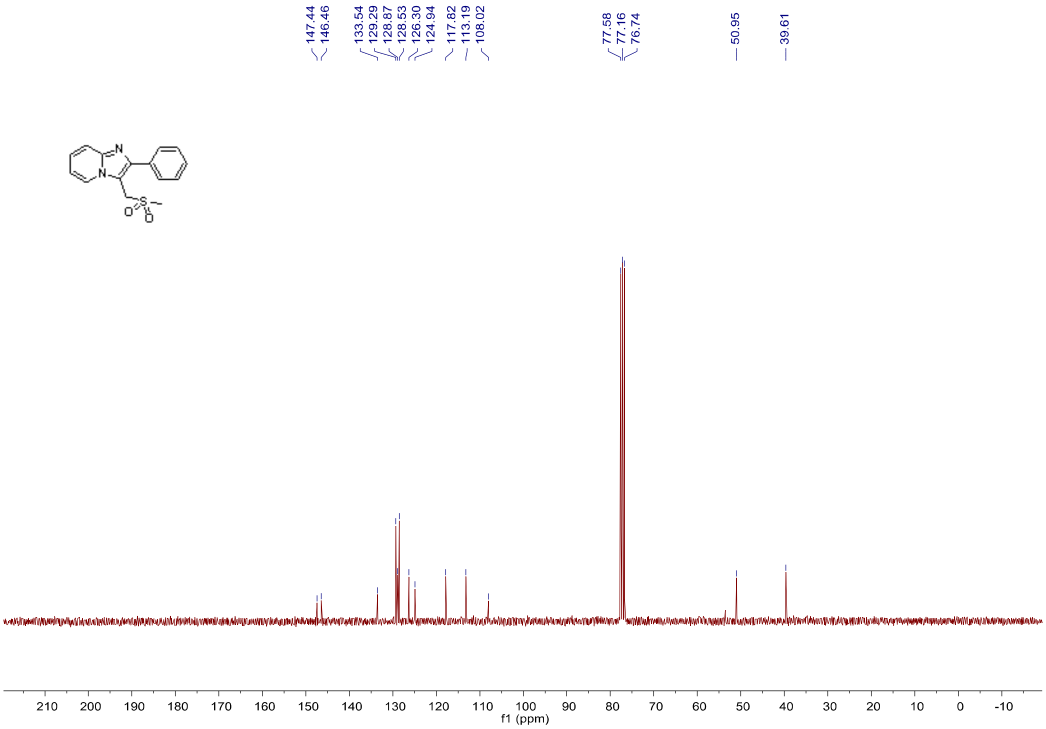

^{1}H NMR (300 MHz, $CDCl_3$) of **3qa**

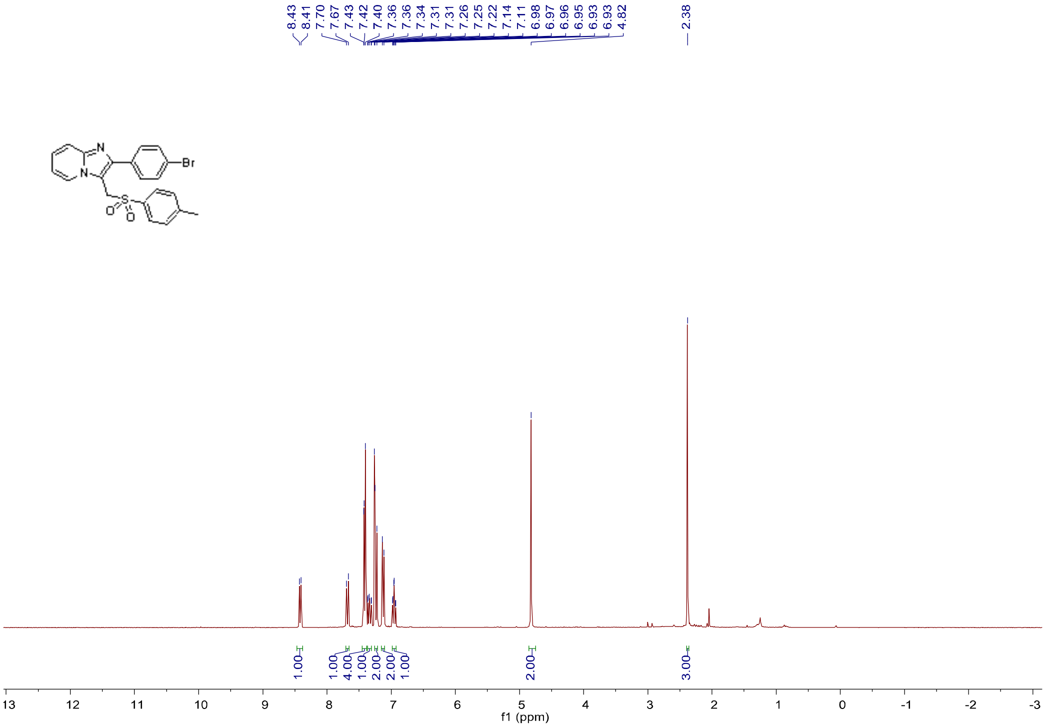

$^{13}C\{^{1}H\}$ (75 MHz, $CDCl_3$) NMR of **3qa**

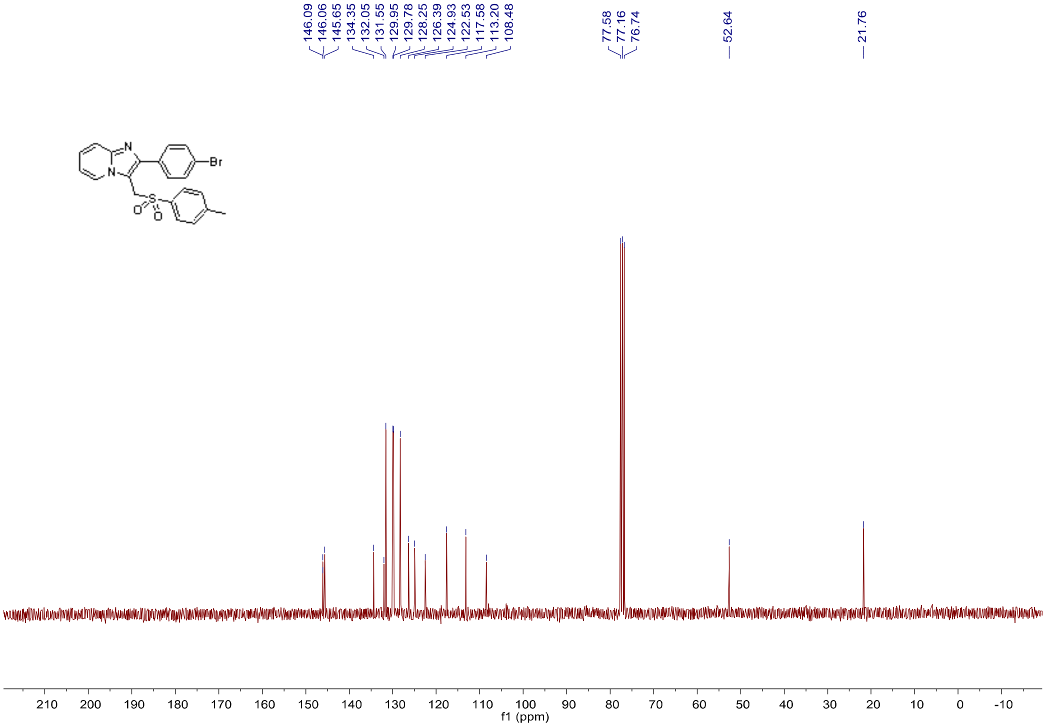

^{1}H NMR (300 MHz, $CDCl_3$) of **3qb**

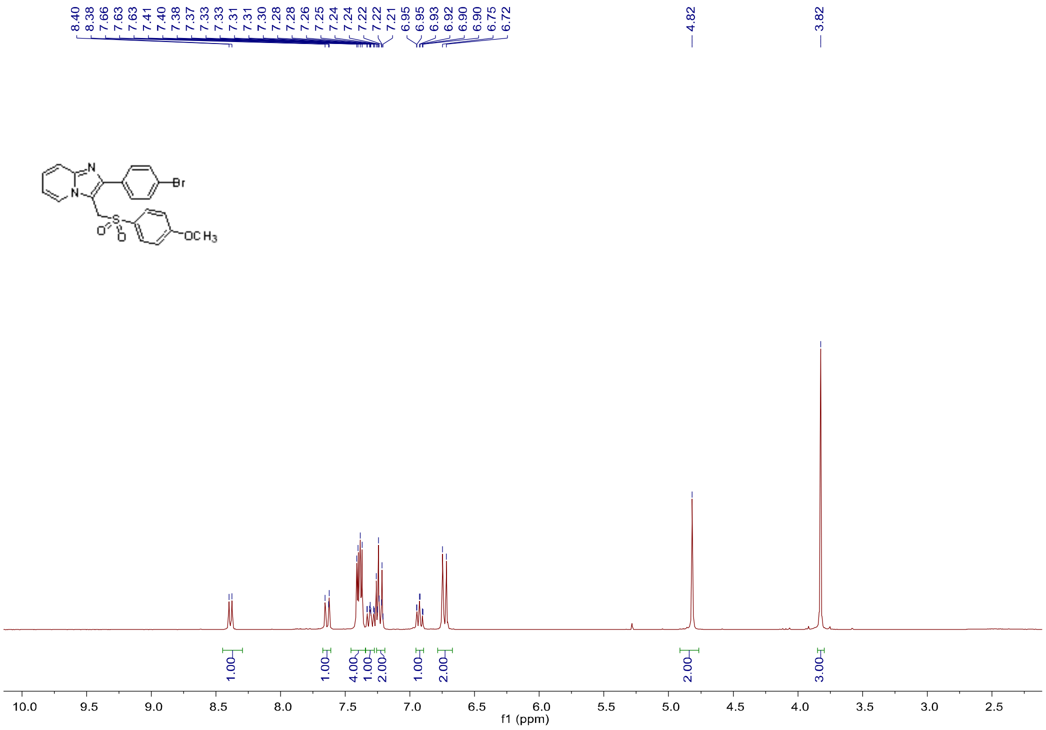

$^{13}C\{^{1}H\}$ (75 MHz, $CDCl_3$) NMR of **3qb**

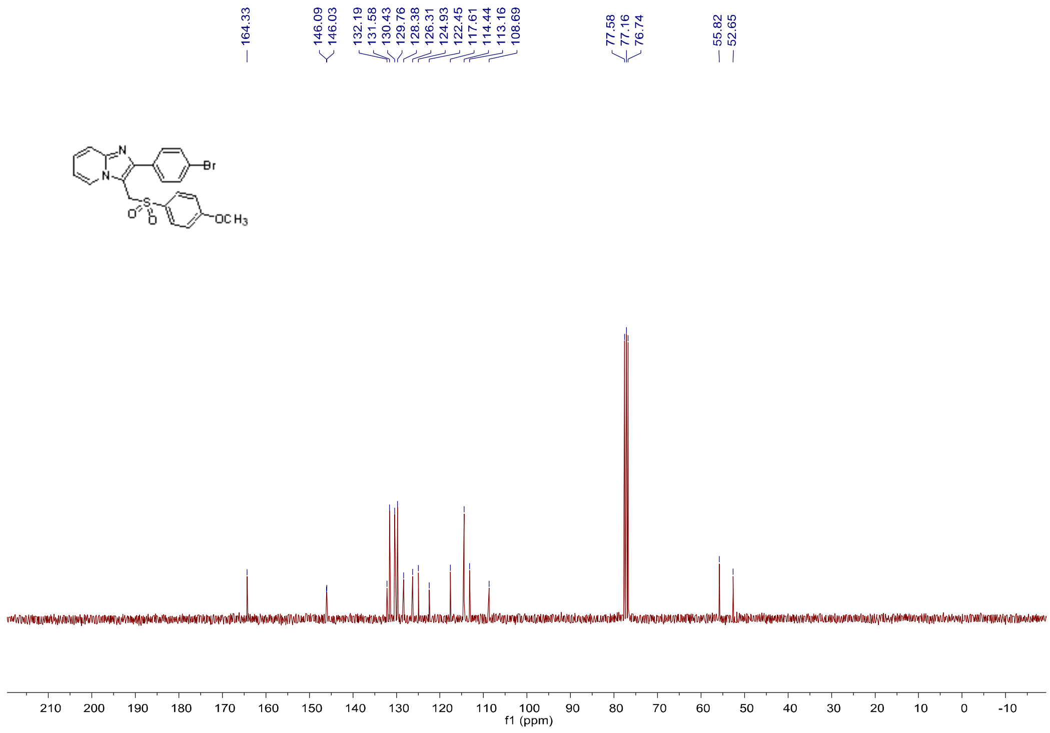

^{1}H NMR (300 MHz, $CDCl_3$) of **3qc**

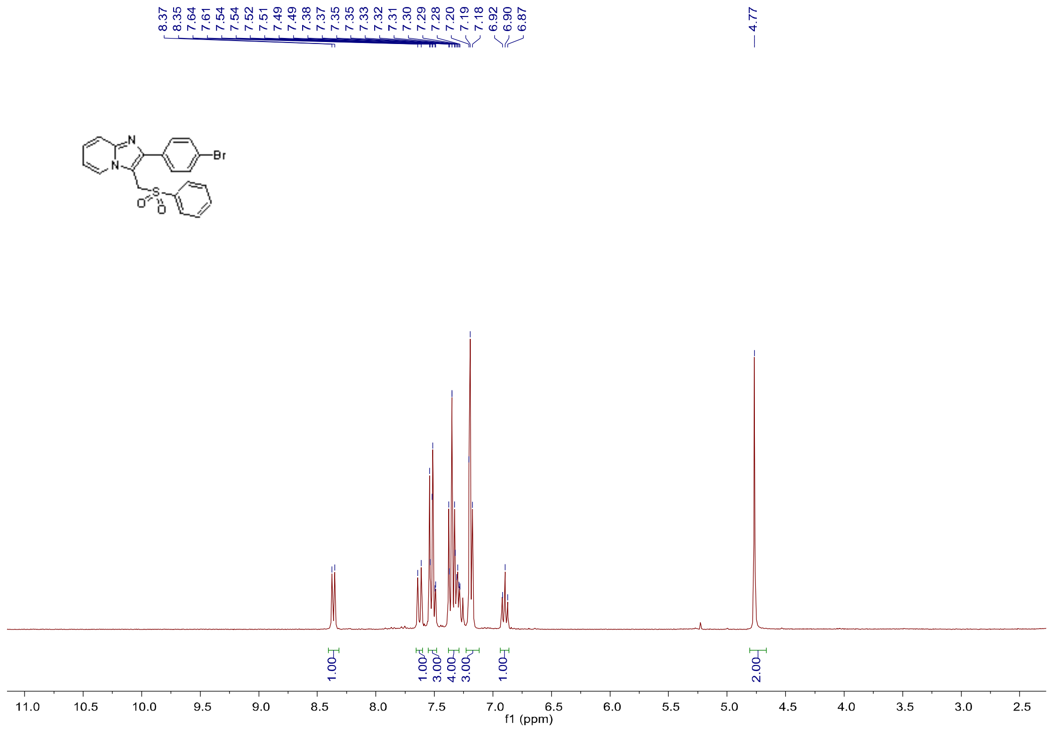

$^{13}C\{^{1}H\}$ (75 MHz, $CDCl_3$) NMR of **3qc**

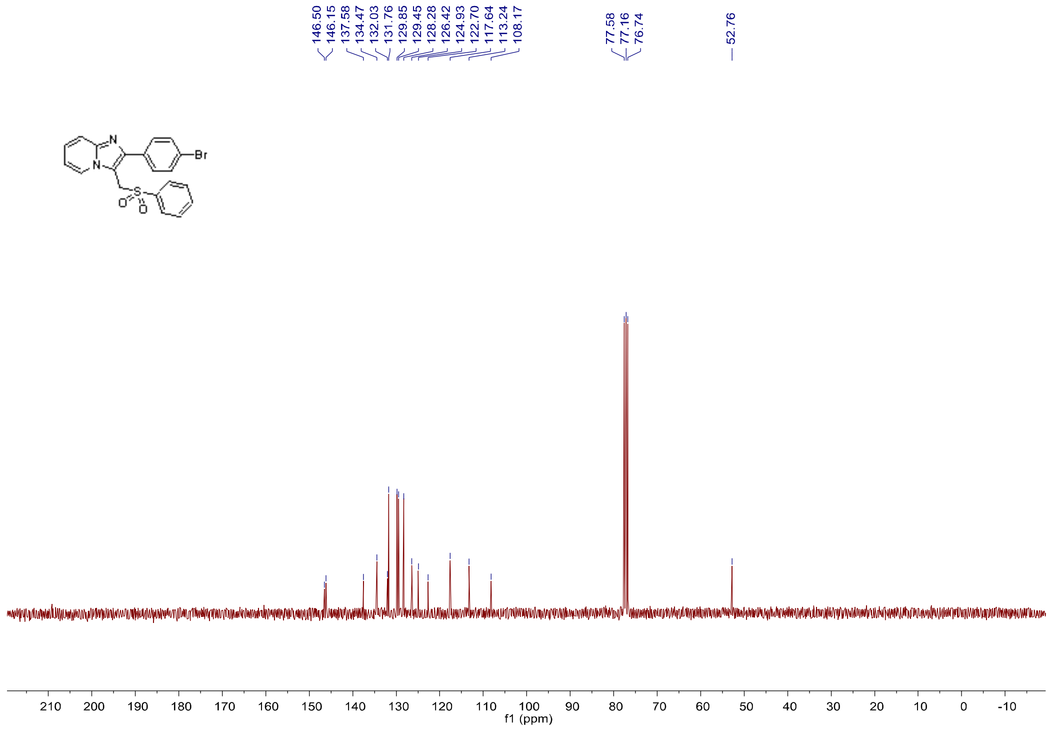

^{1}H NMR (300 MHz, $CDCl_3$) of **3qd**

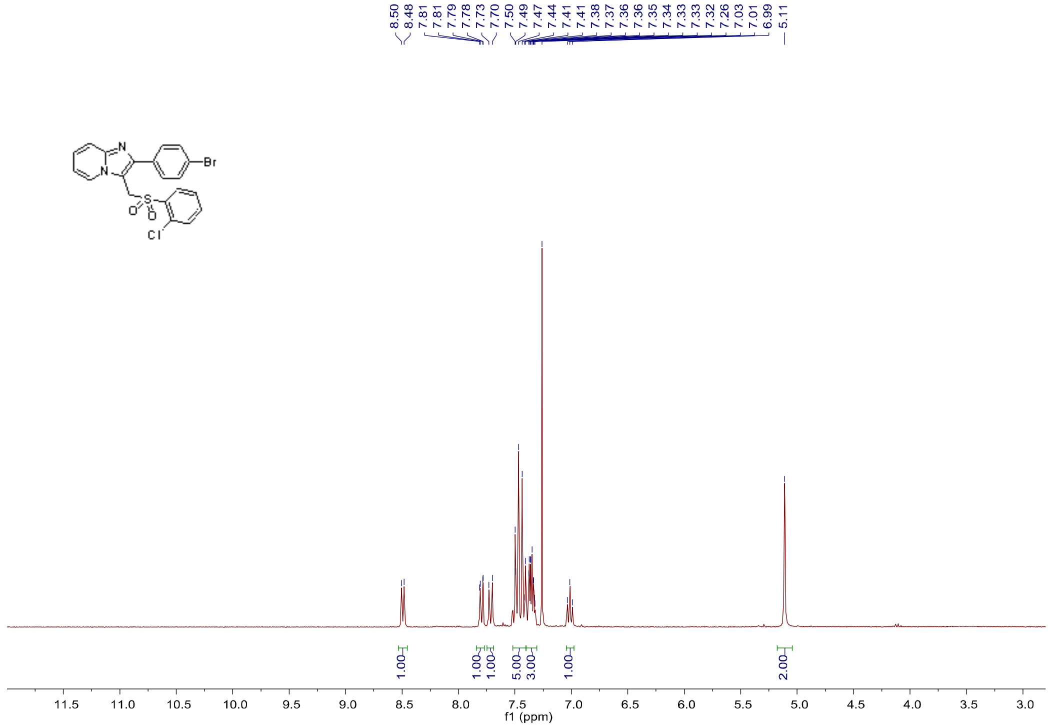

$^{13}C\{^{1}H\}$ (75 MHz, $CDCl_3$) NMR of **3qd**

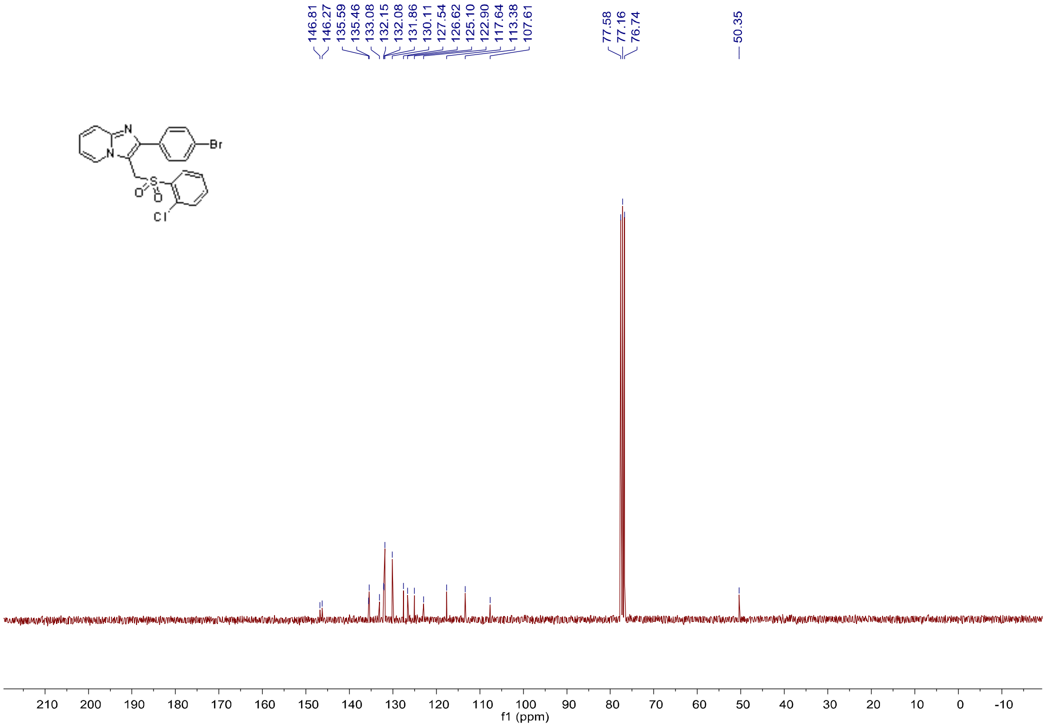

^{1}H NMR (300 MHz, $CDCl_3$) of **3qe**

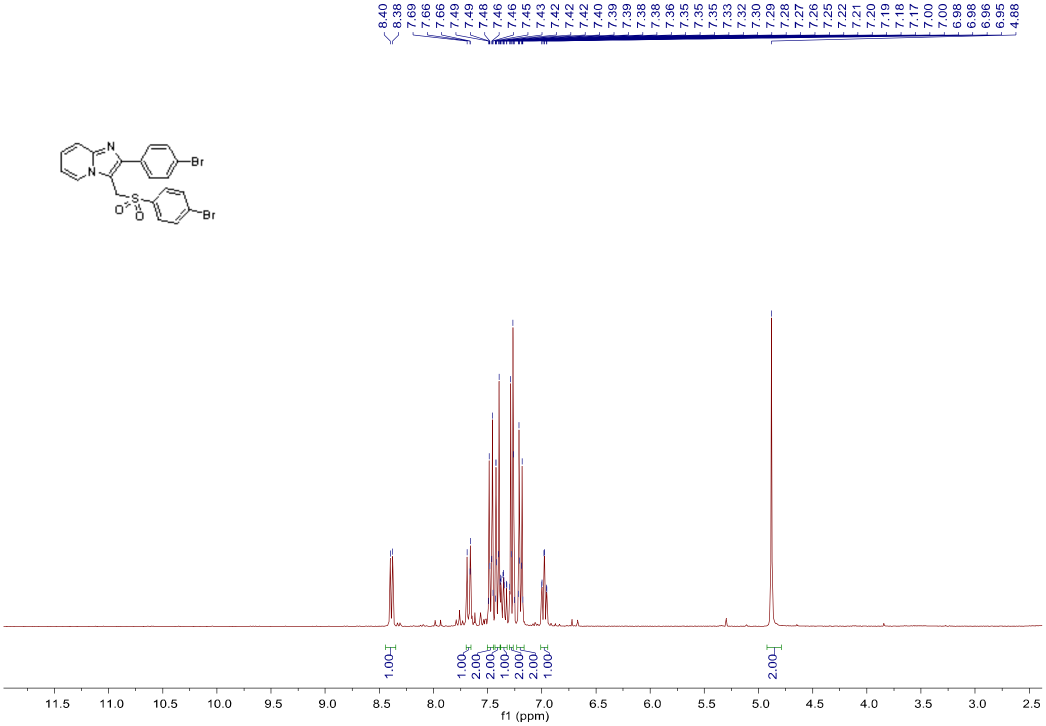

$^{13}C\{^{1}H\}$ (75 MHz, $CDCl_3$) NMR of **3qe**

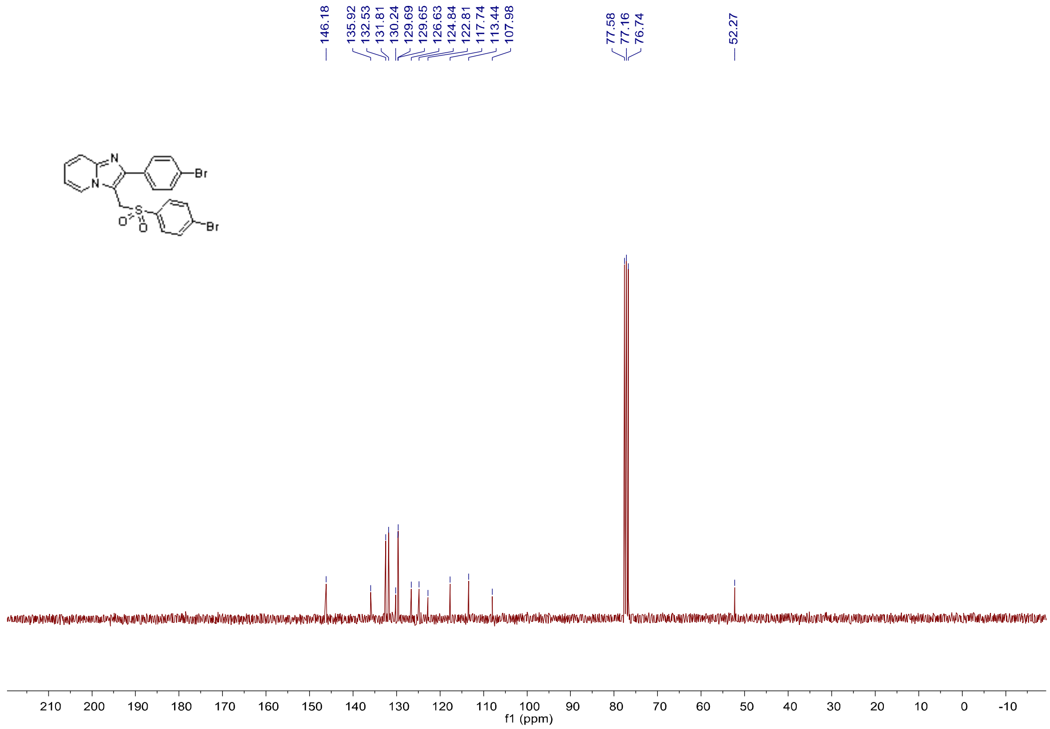

^{1}H NMR (300 MHz, $CDCl_3$) of **3qf**

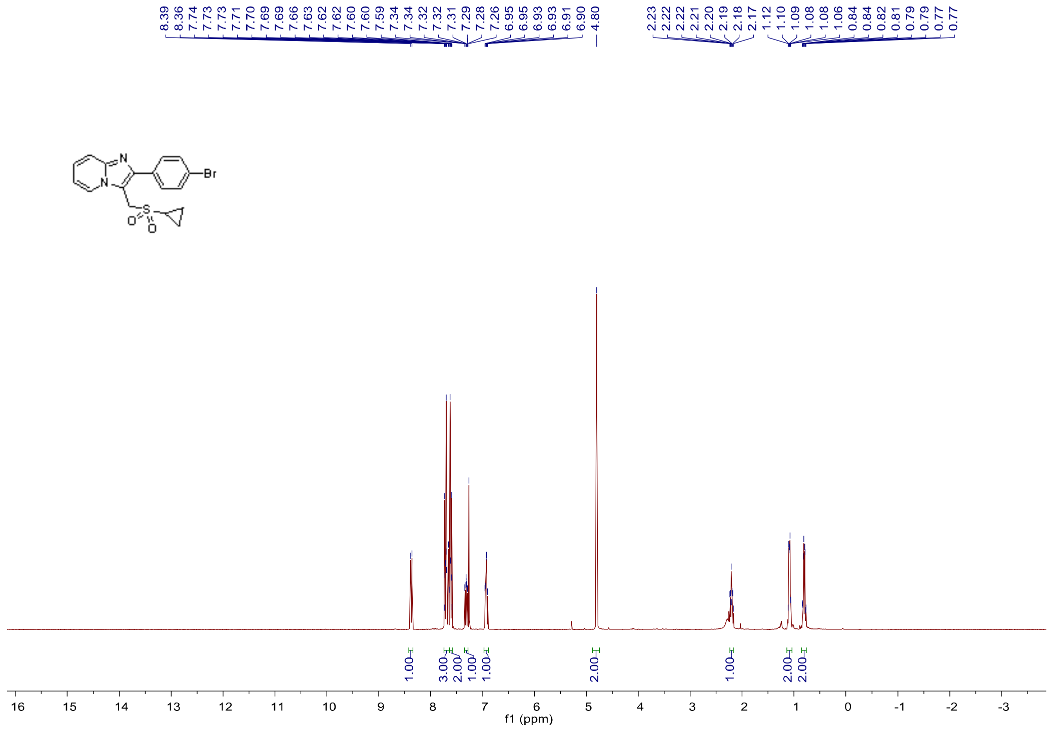

$^{13}C\{^1H\}$ (75 MHz, $CDCl_3$) NMR of **3qf**

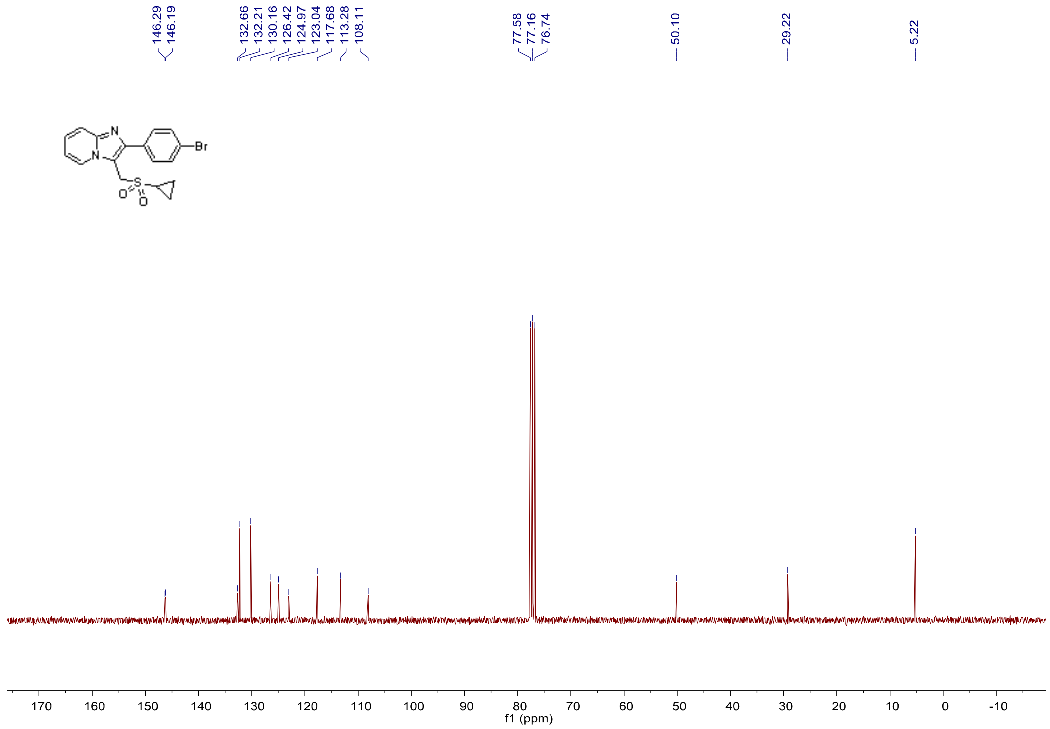

^1H NMR (300 MHz, $CDCl_3$) of **3ra**

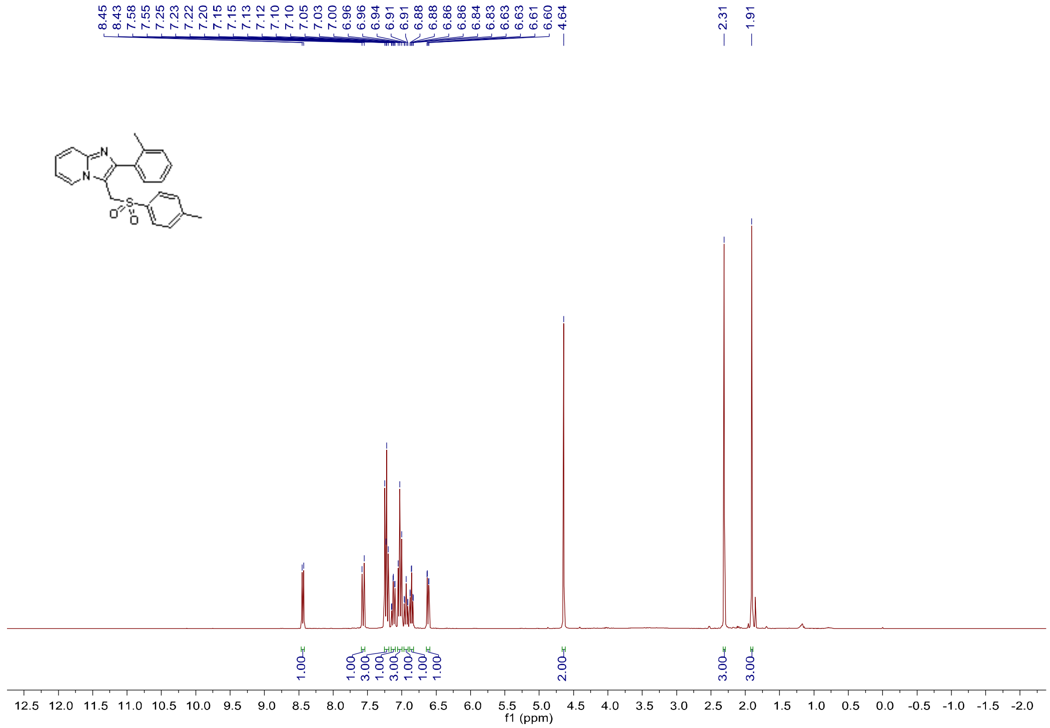

$^{13}C\{^{1}H\}$ (75 MHz, $CDCl_3$) NMR of **3ra**

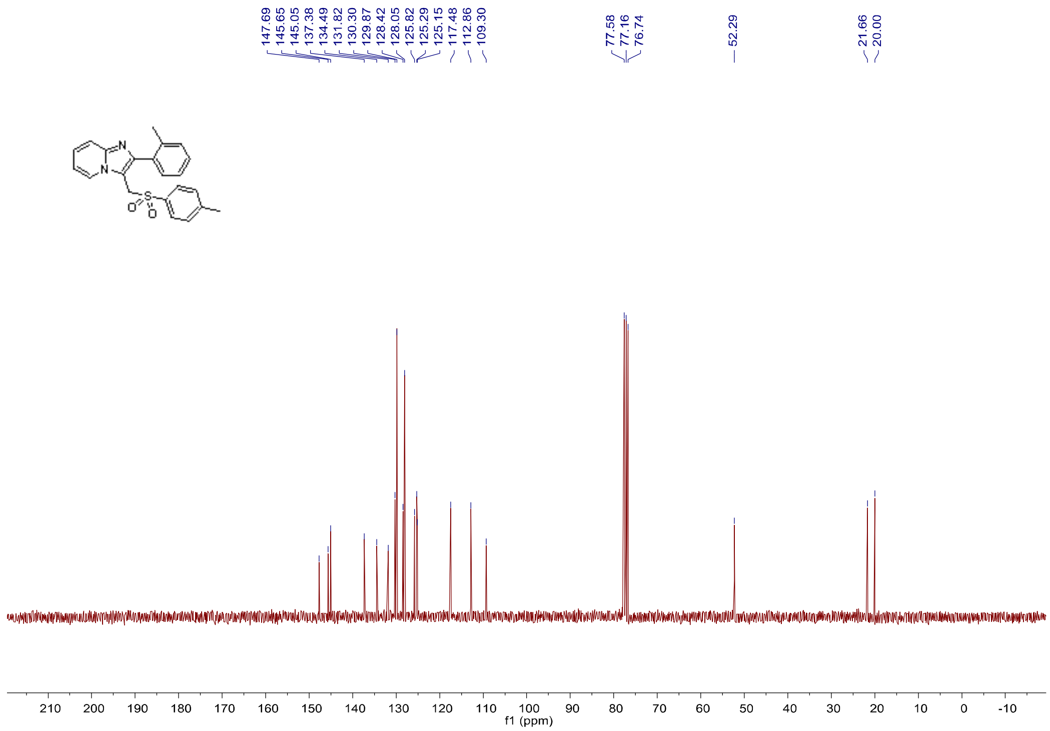

^{1}H NMR (300 MHz, $CDCl_3$) of **3rb**

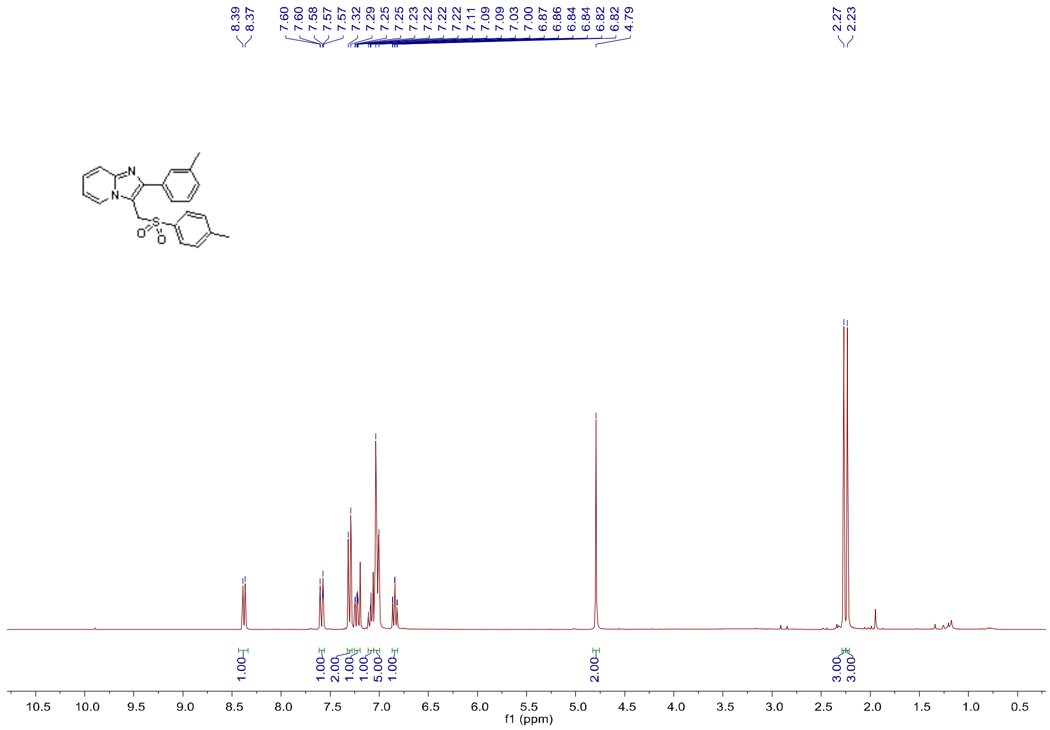

$^{13}C\{^{1}H\}$ (75 MHz, $CDCl_3$) NMR of **3rb**

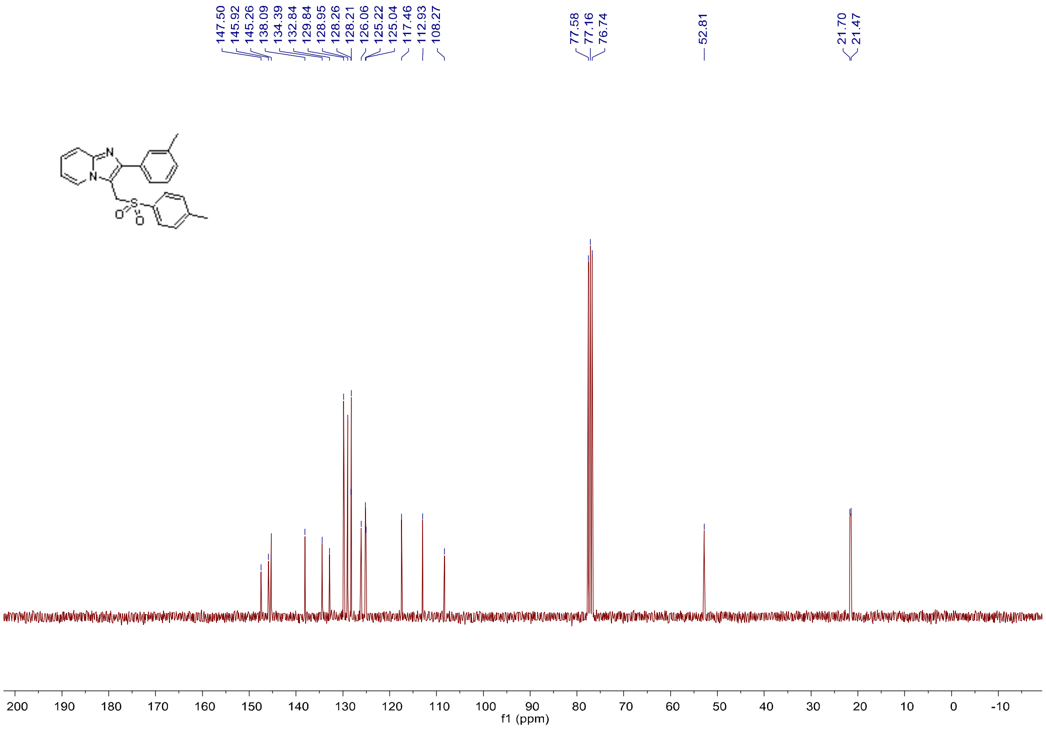

^{1}H NMR (300 MHz, $CDCl_3$) of **3rc**

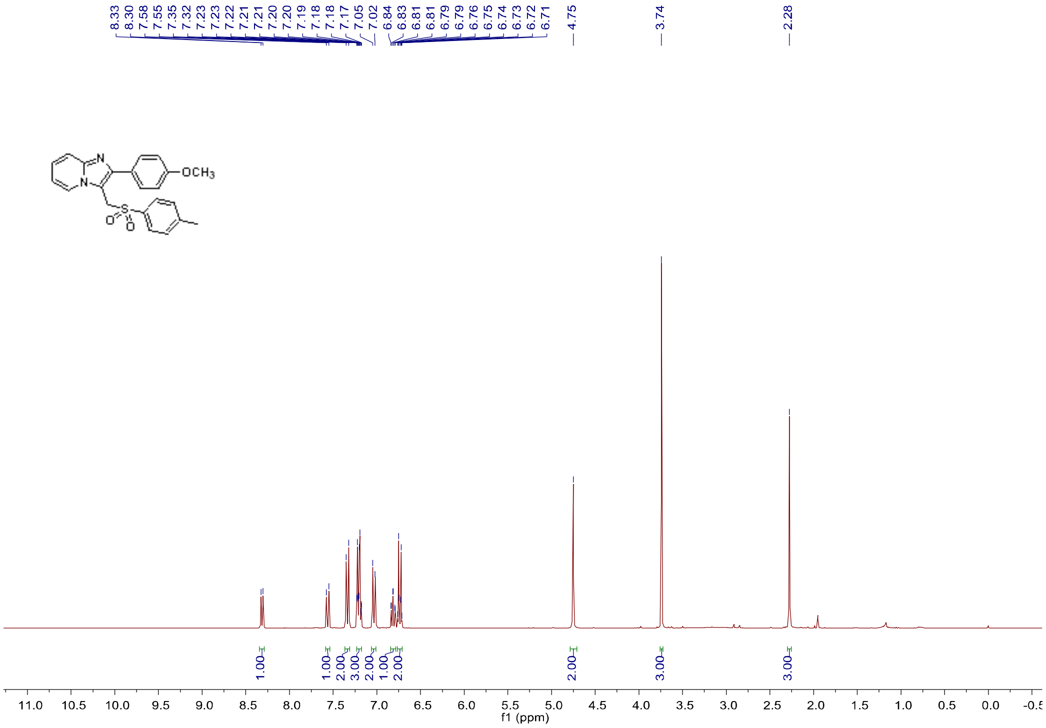

$^{13}C\{^1H\}$ (75 MHz, $CDCl_3$) NMR of **3rc**

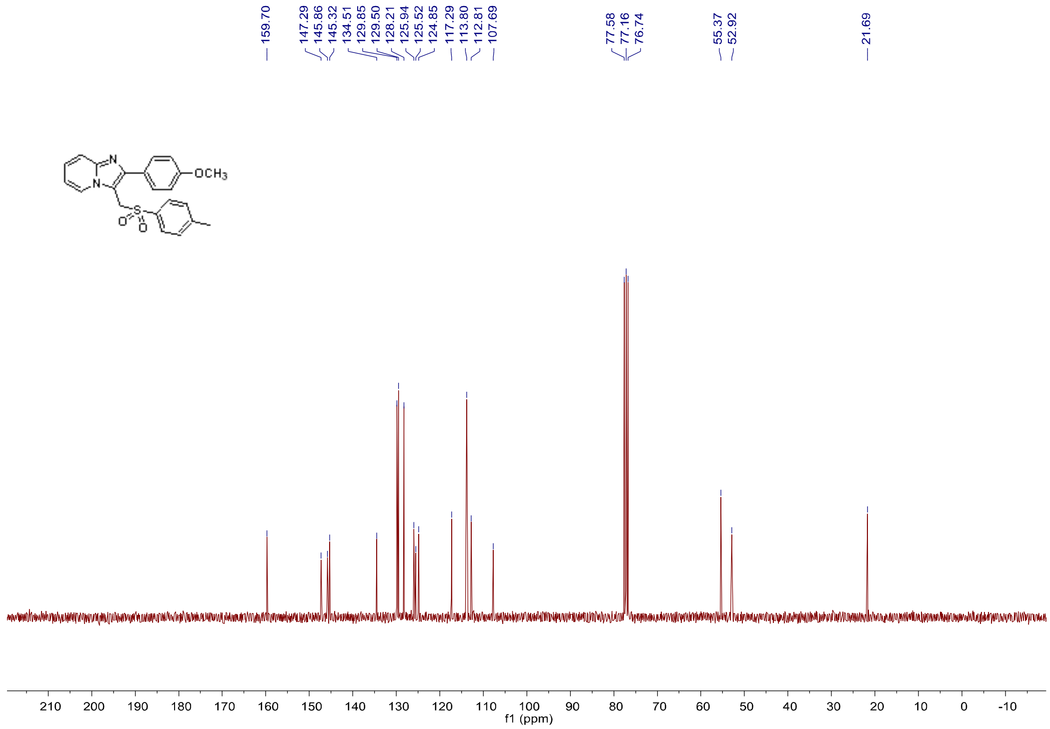

^1H NMR (300 MHz, $CDCl_3$) of **3rd**

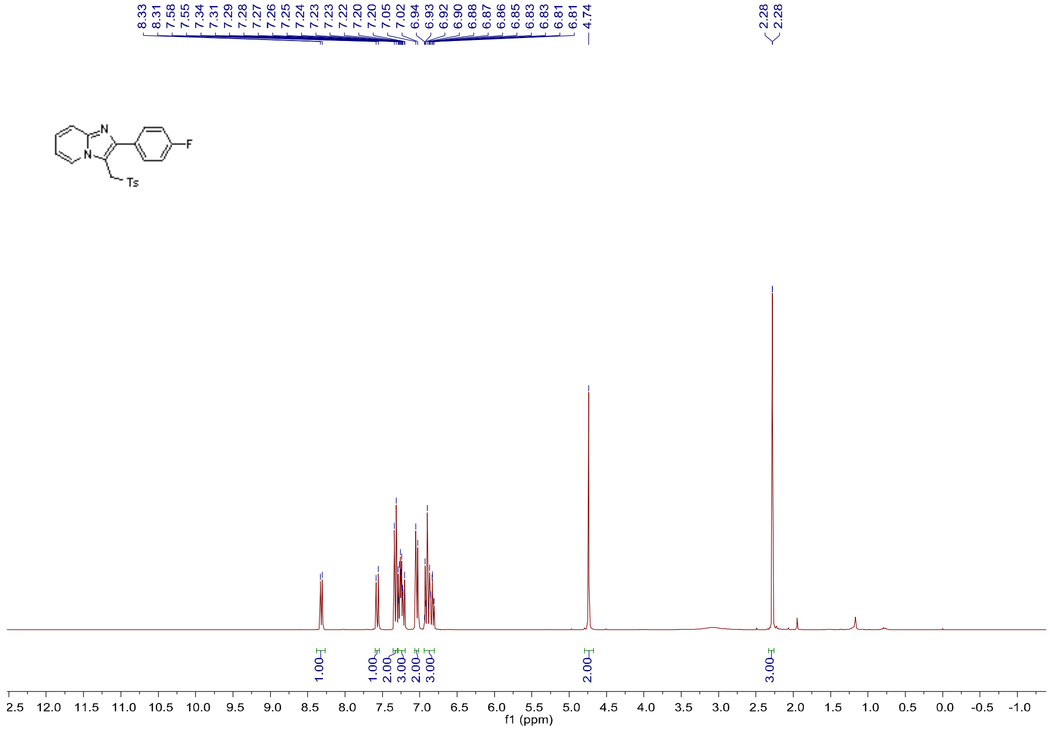

$^{13}C\{^{1}H\}$ (75 MHz, $CDCl_3$) NMR of **3rd**

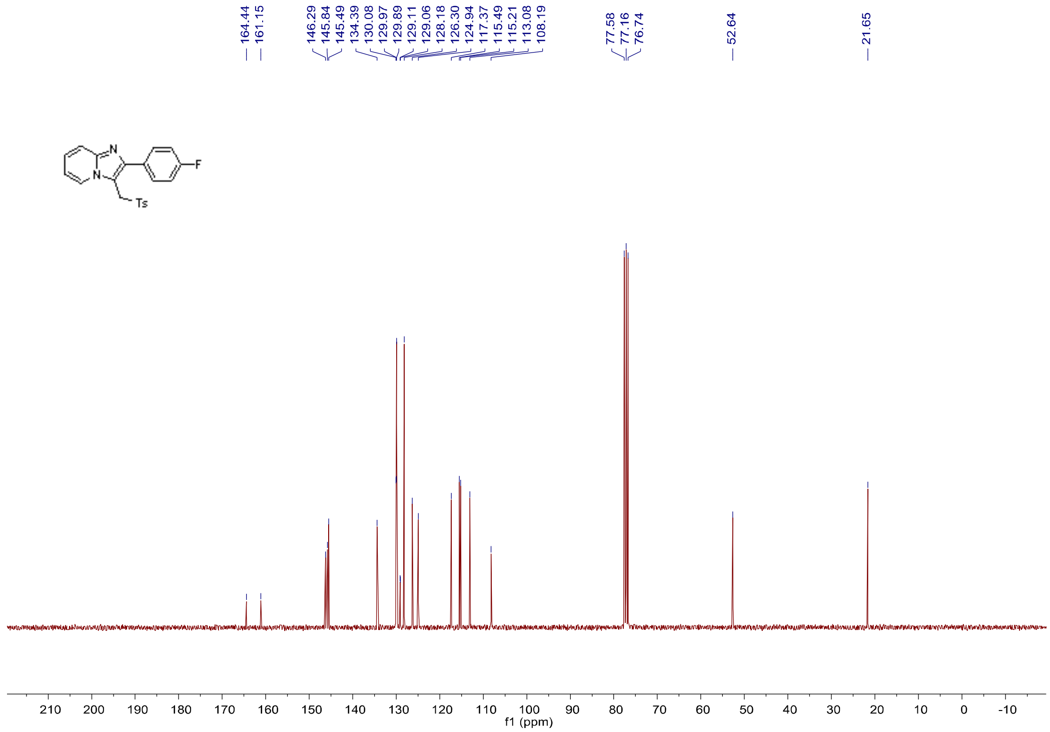

^{1}H NMR (300 MHz, $CDCl_3$) of **3re**

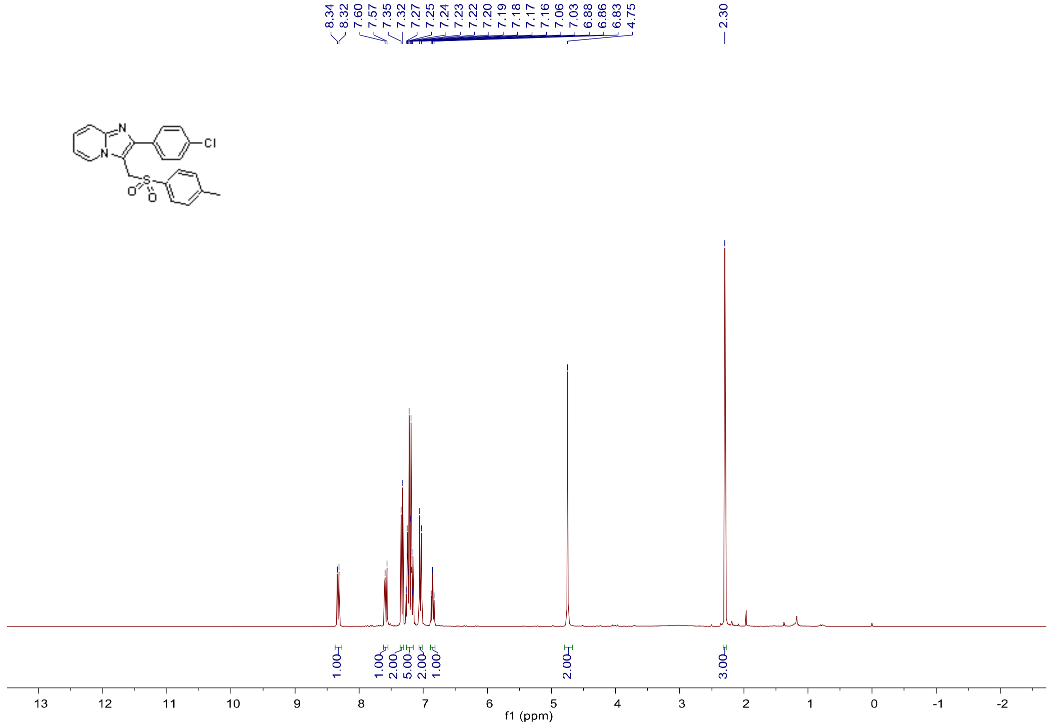

$^{13}C\{^{1}H\}$ (75 MHz, $CDCl_3$) NMR of **3re**

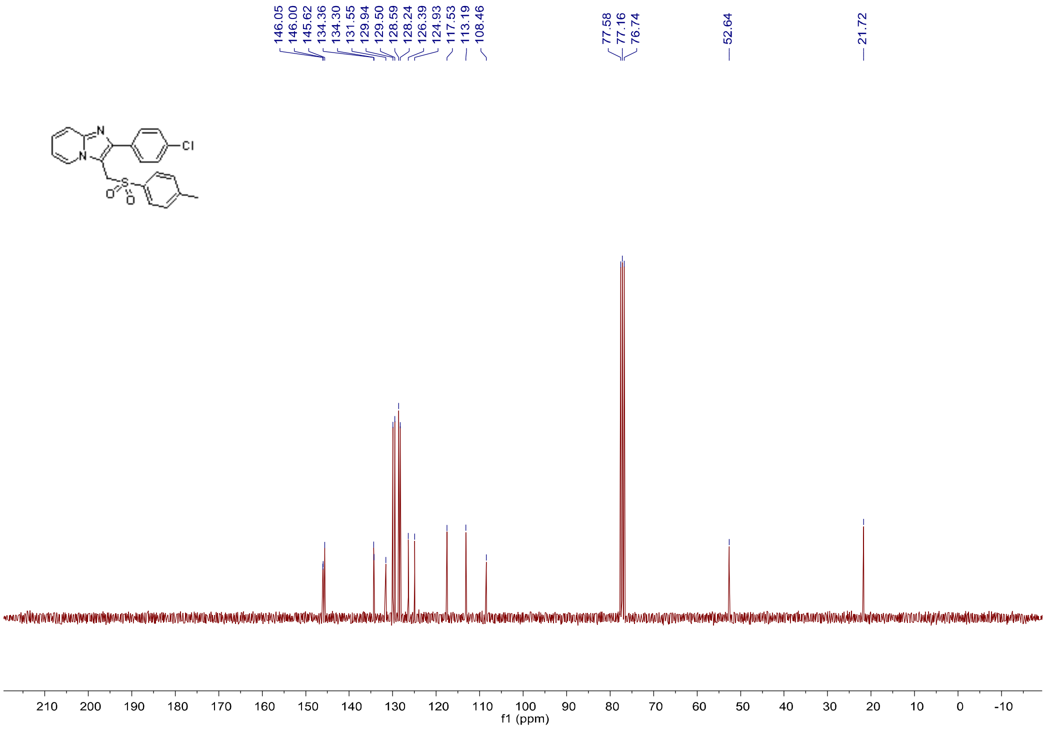

^{1}H NMR (300 MHz, $CDCl_3$) of **3rf**

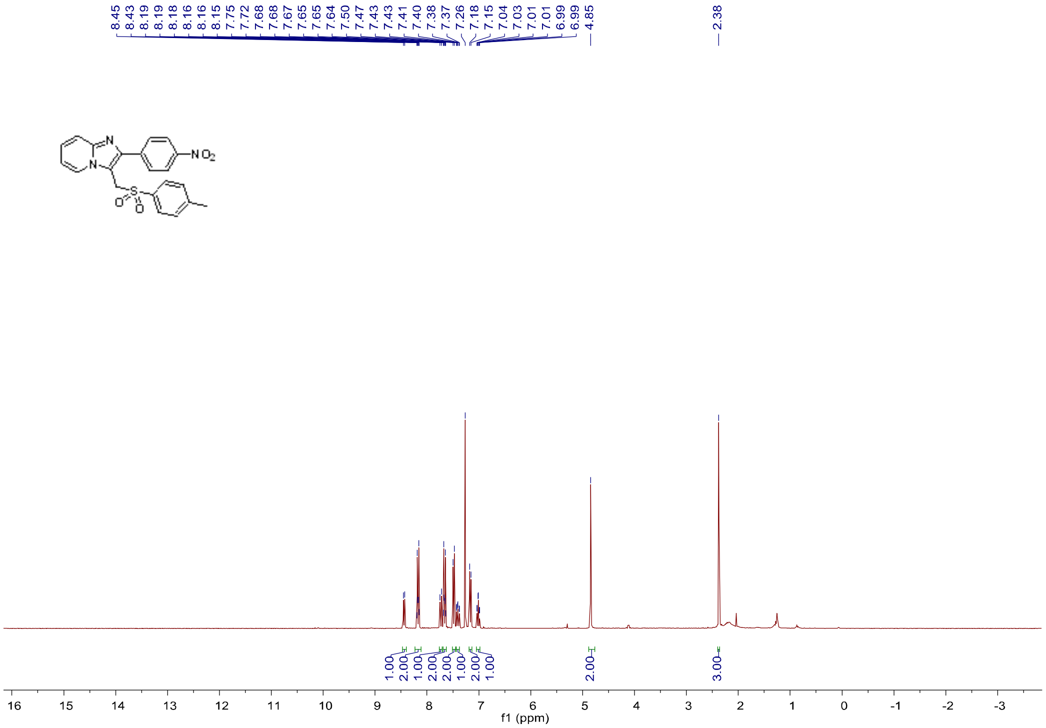

$^{13}C\{^{1}H\}$ (75 MHz, $CDCl_3$) NMR of **3rf**

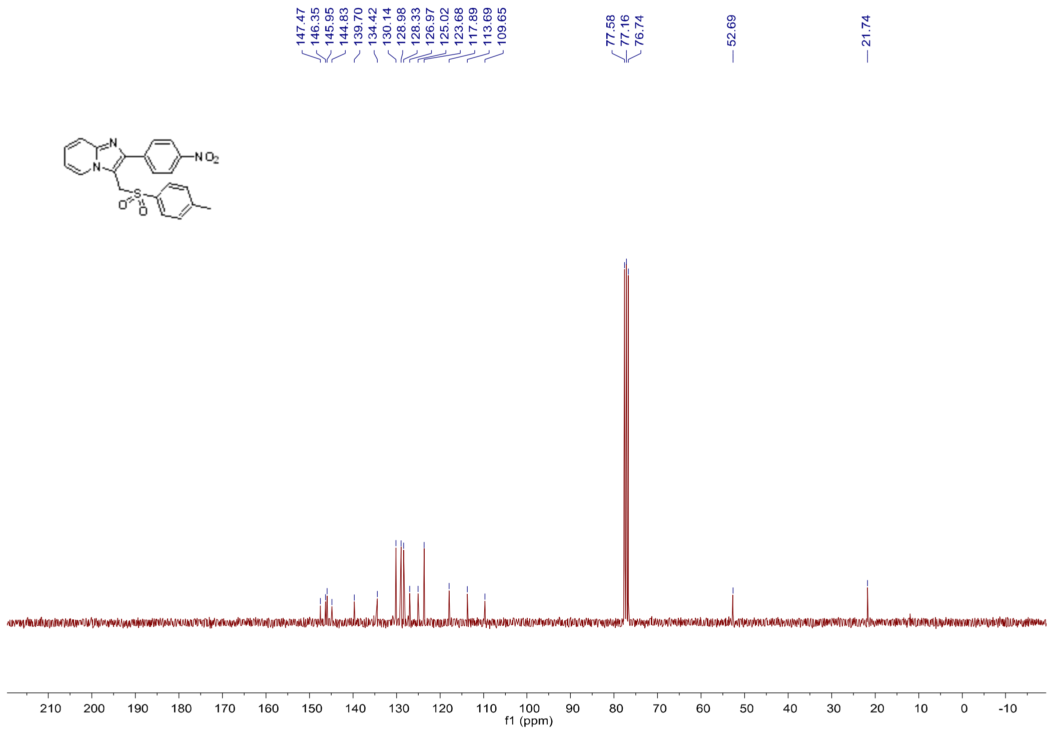

^{1}H NMR (300 MHz, $CDCl_3$) of **3sa**

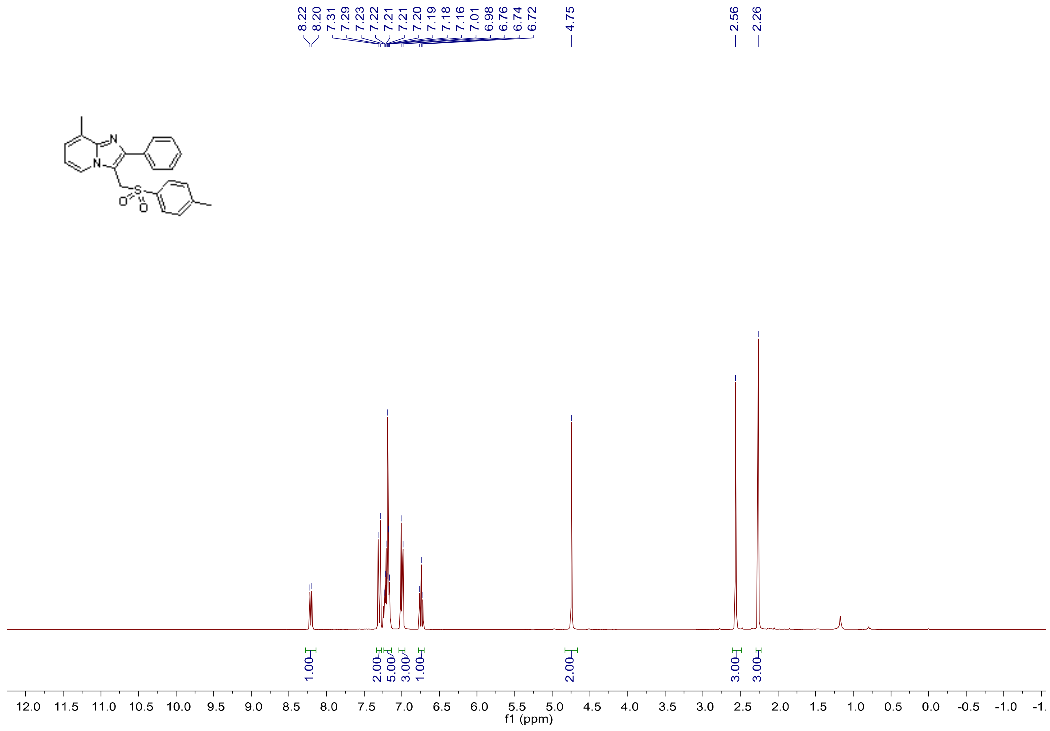

$^{13}C\{^{1}H\}$ (75 MHz, $CDCl_3$) NMR of **3sa**

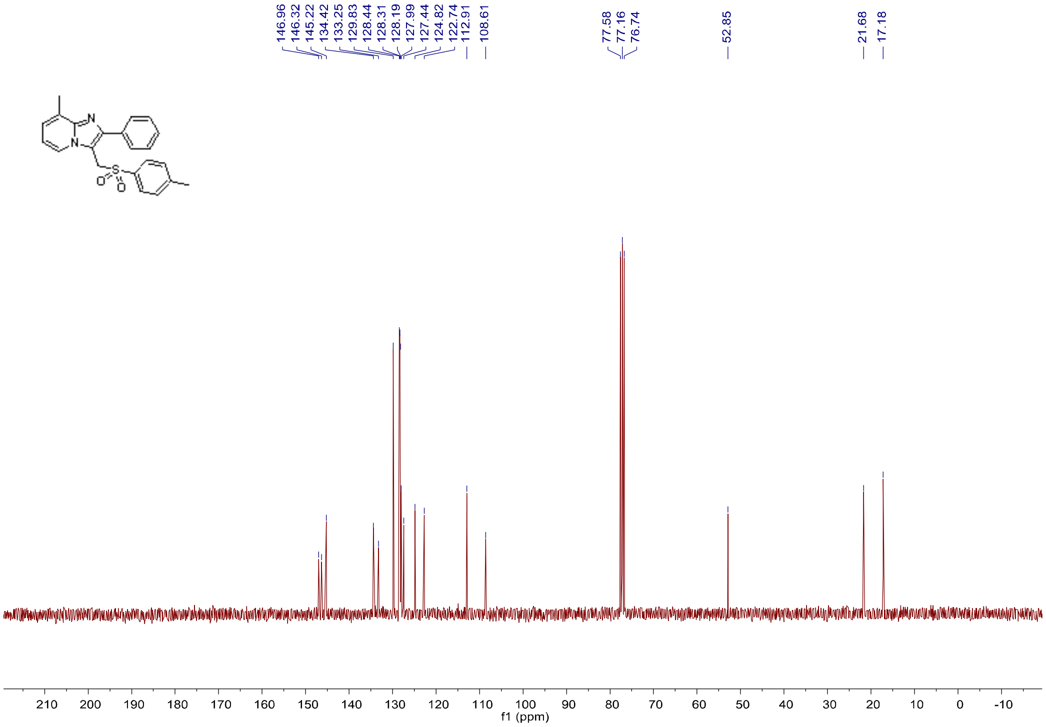

^{1}H NMR (300 MHz, $CDCl_3$) of **3sb**

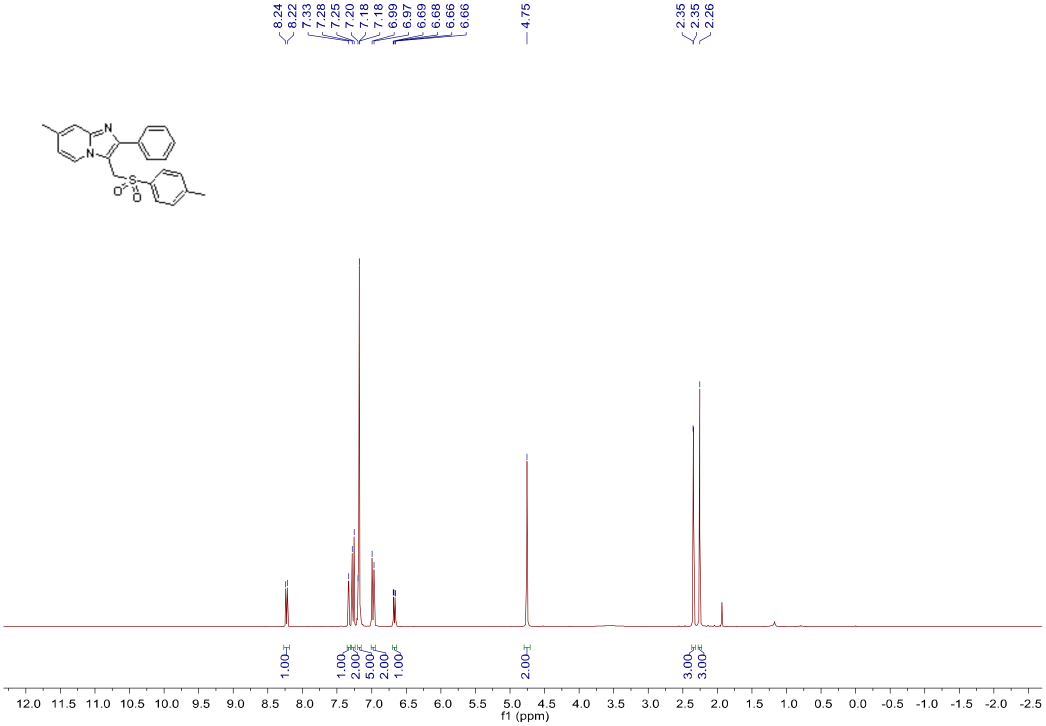

$^{13}C\{^{1}H\}$ (75 MHz, $CDCl_3$) NMR of **3sb**

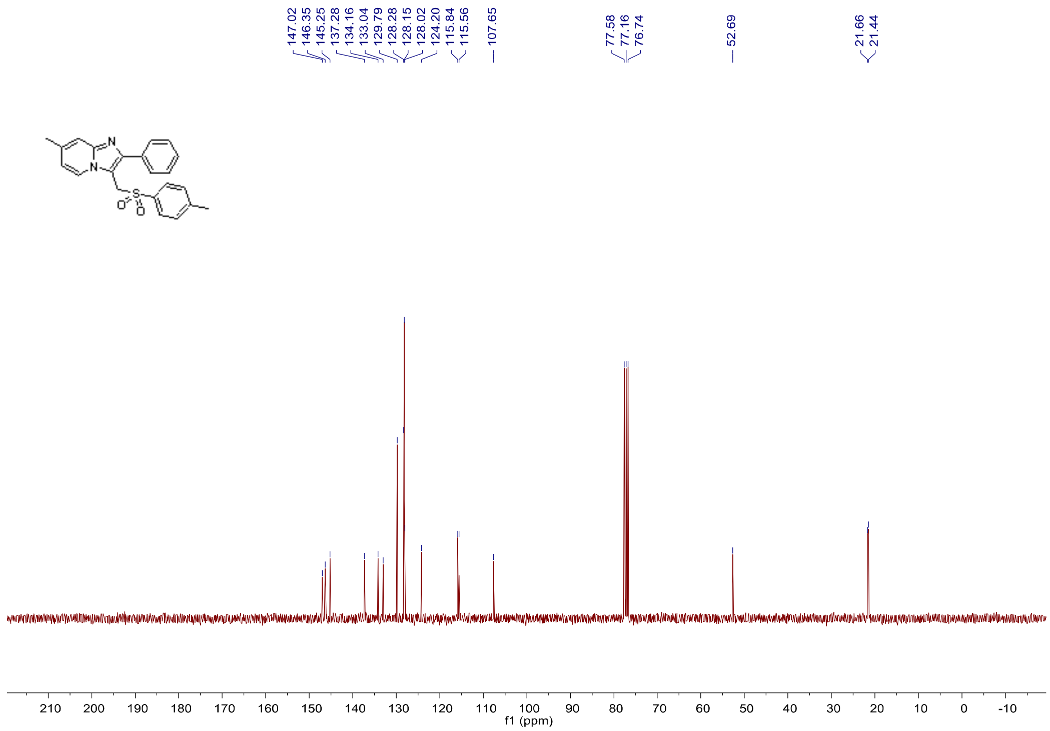

^{1}H NMR (300 MHz, $CDCl_3$) of **3sc**

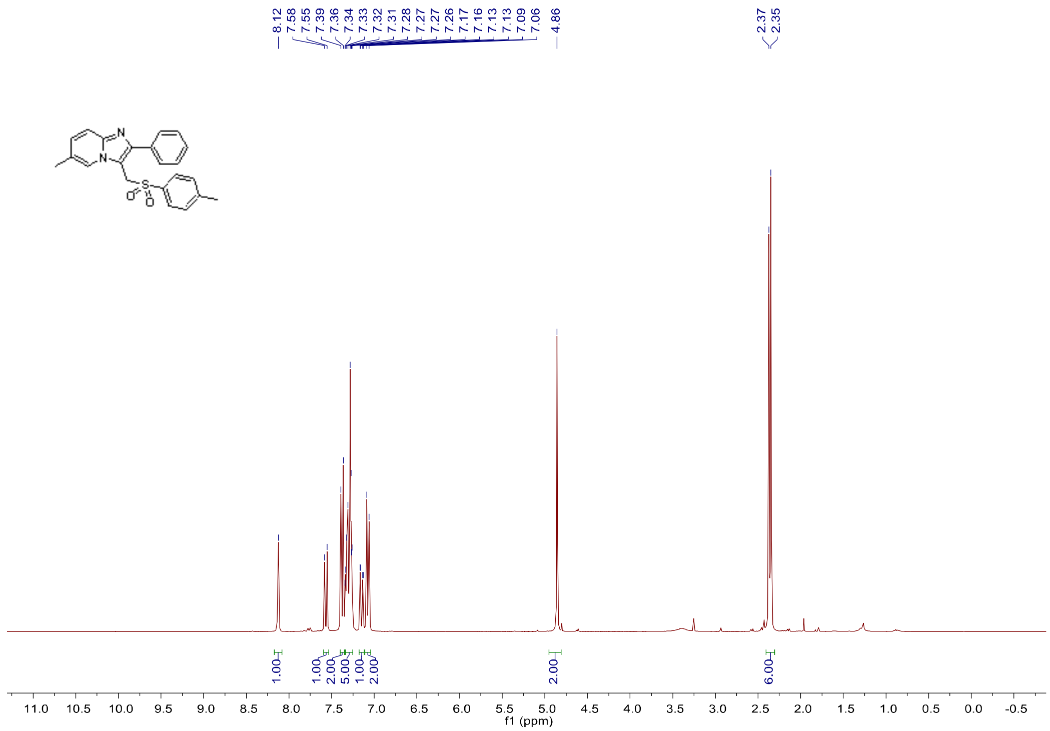

$^{13}C\{^{1}H\}$ (75 MHz, $CDCl_3$) NMR of **3sc**

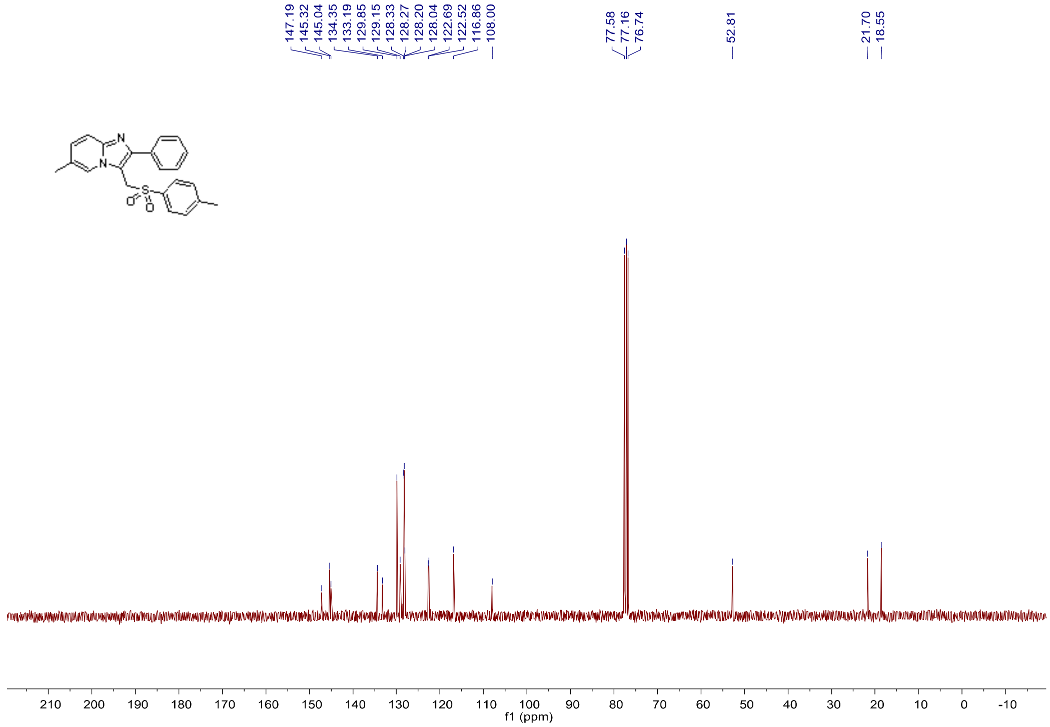

^{1}H NMR (300 MHz, $CDCl_3$) of **3sd**

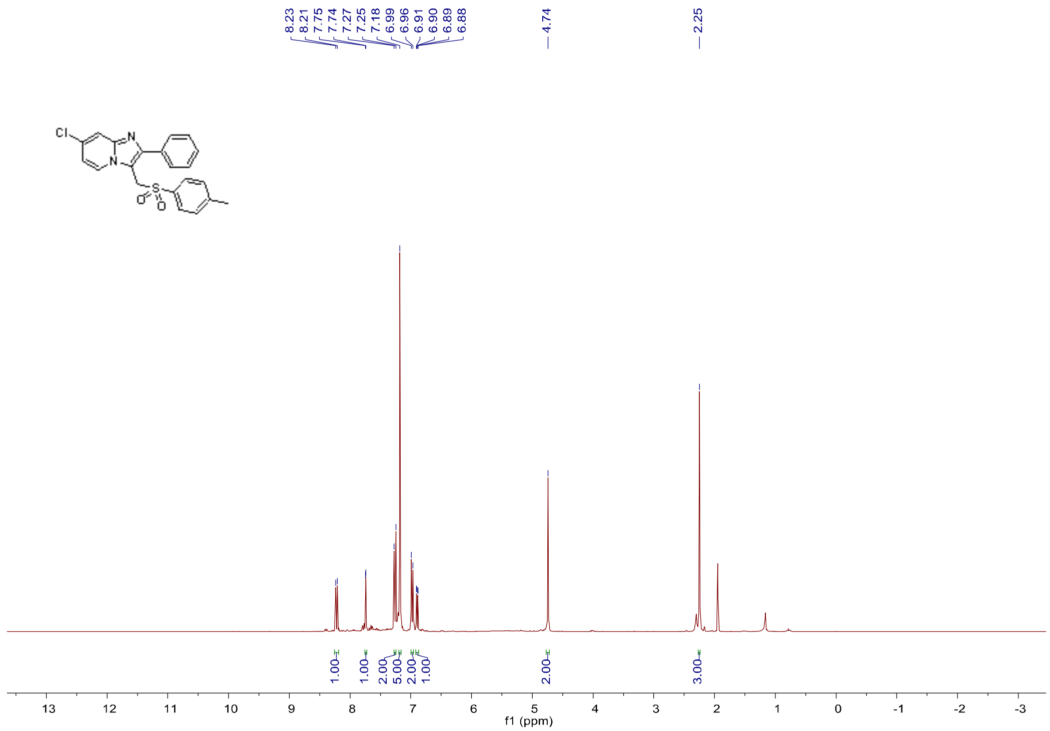

$^{13}C\{^{1}H\}$ (75 MHz, $CDCl_3$) NMR of **3sd**

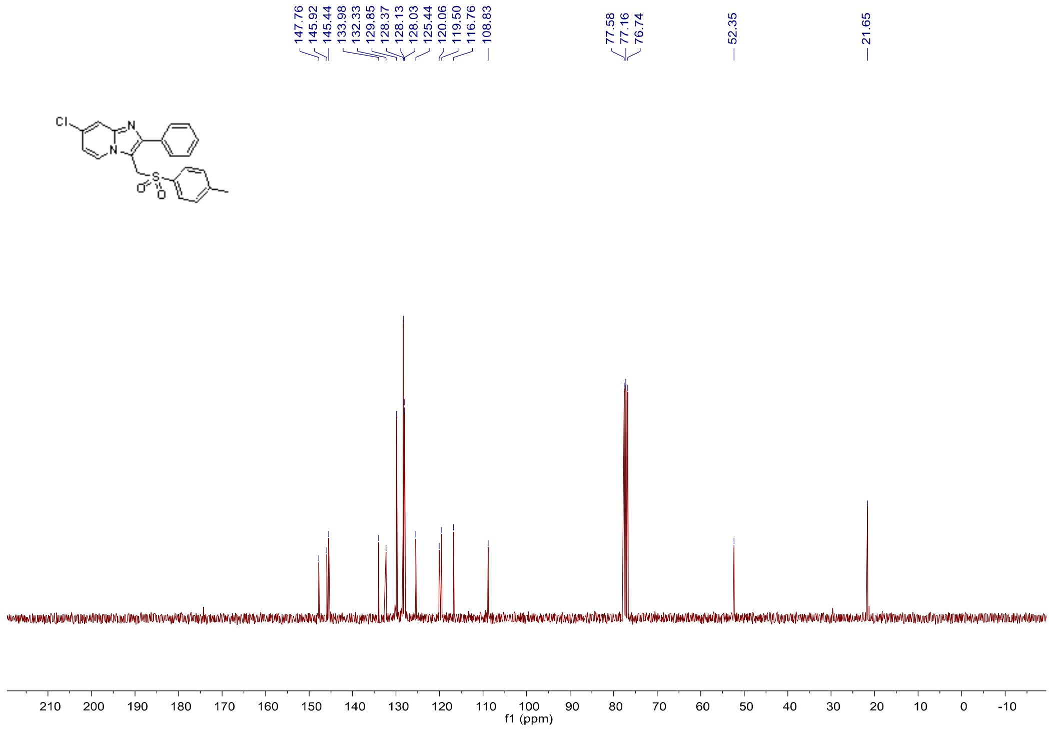

^{1}H NMR (300 MHz, $CDCl_3$) of **3se**

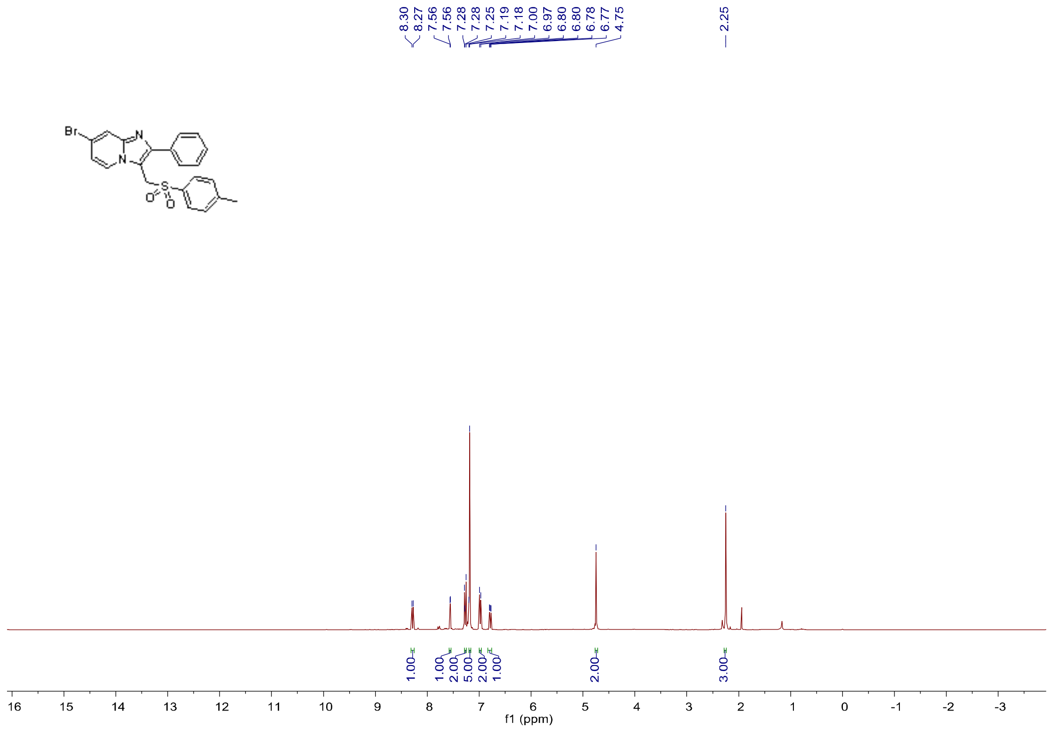

$^{13}C\{^{1}H\}$ (75 MHz, $CDCl_3$) NMR of **3se**

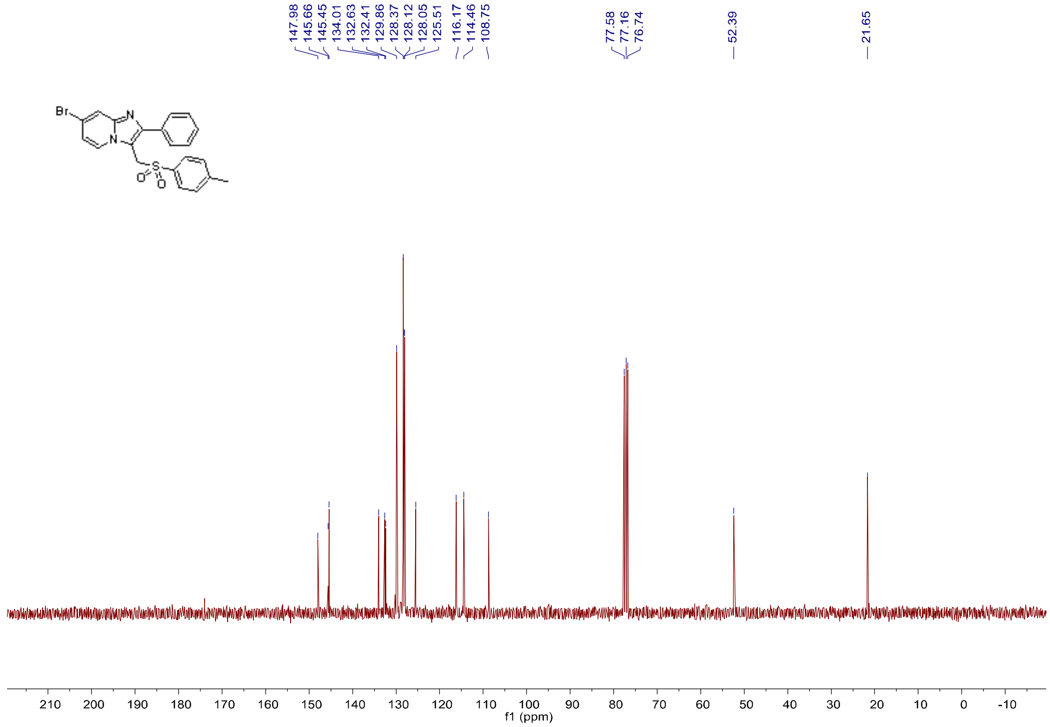

^{1}H NMR (300 MHz, $CDCl_3$) of **3ta**

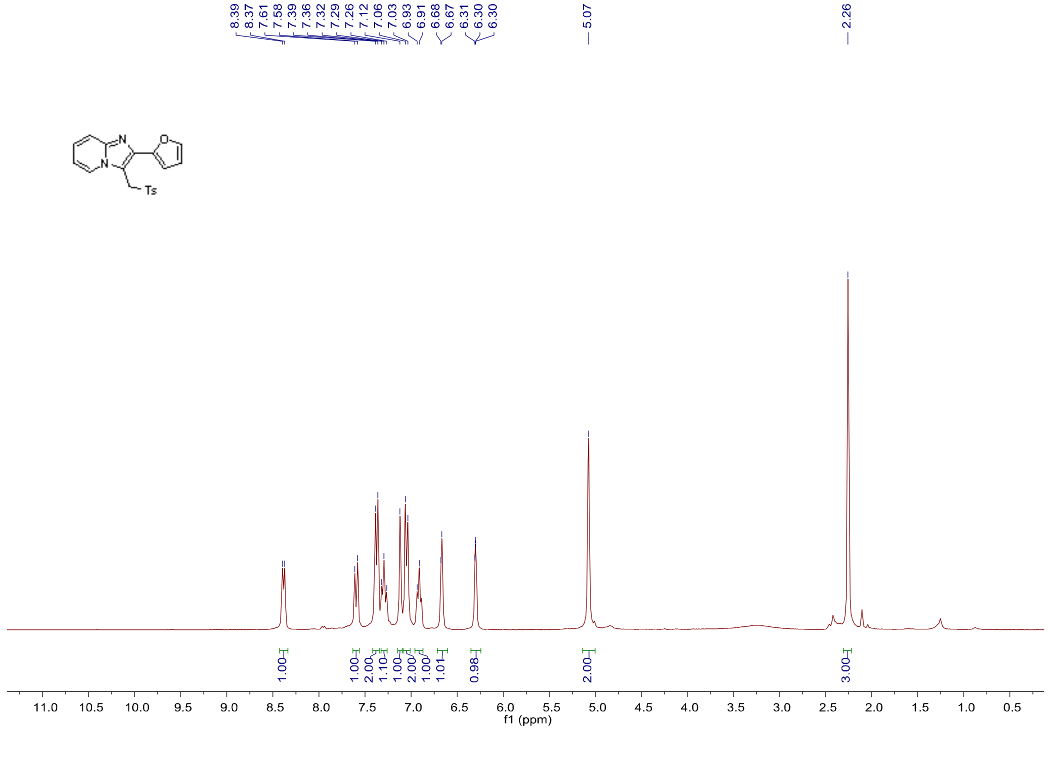

$^{13}C\{^1H\}$ (75 MHz, $CDCl_3$) NMR of **3ta**

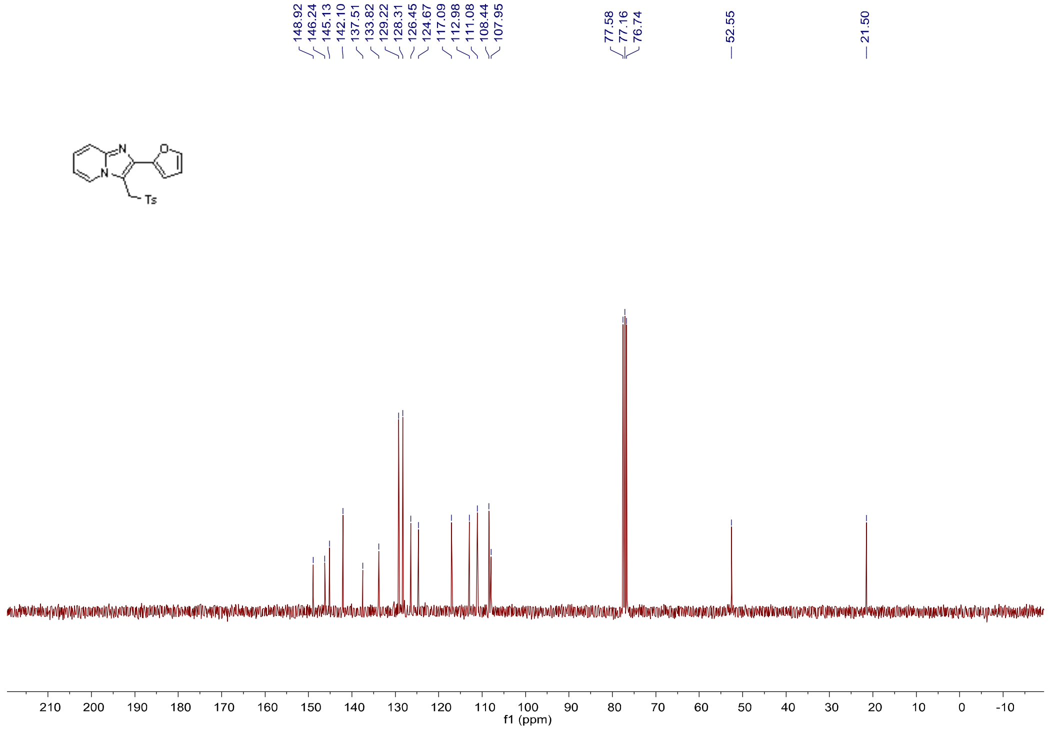

^1H NMR (300 MHz, $CDCl_3$) of **3tb**

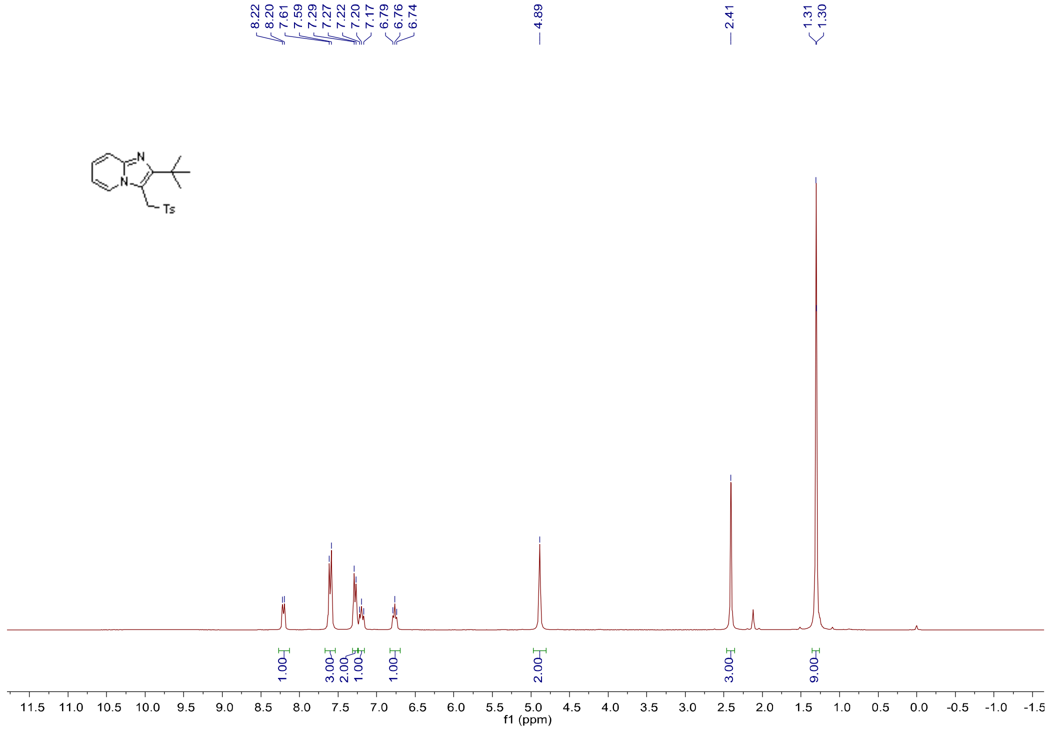

$^{13}C\{^{1}H\}$ (75 MHz, $CDCl_3$) NMR of **3tb**

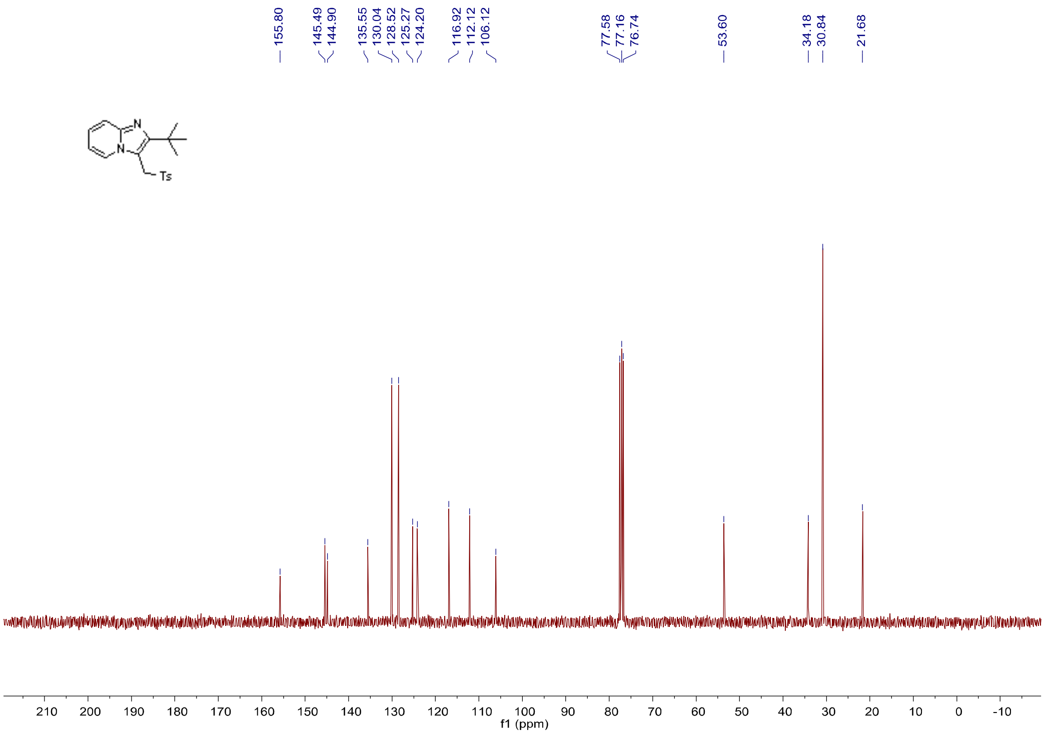

^{1}H NMR (300 MHz, $CDCl_3$) of **3ua**

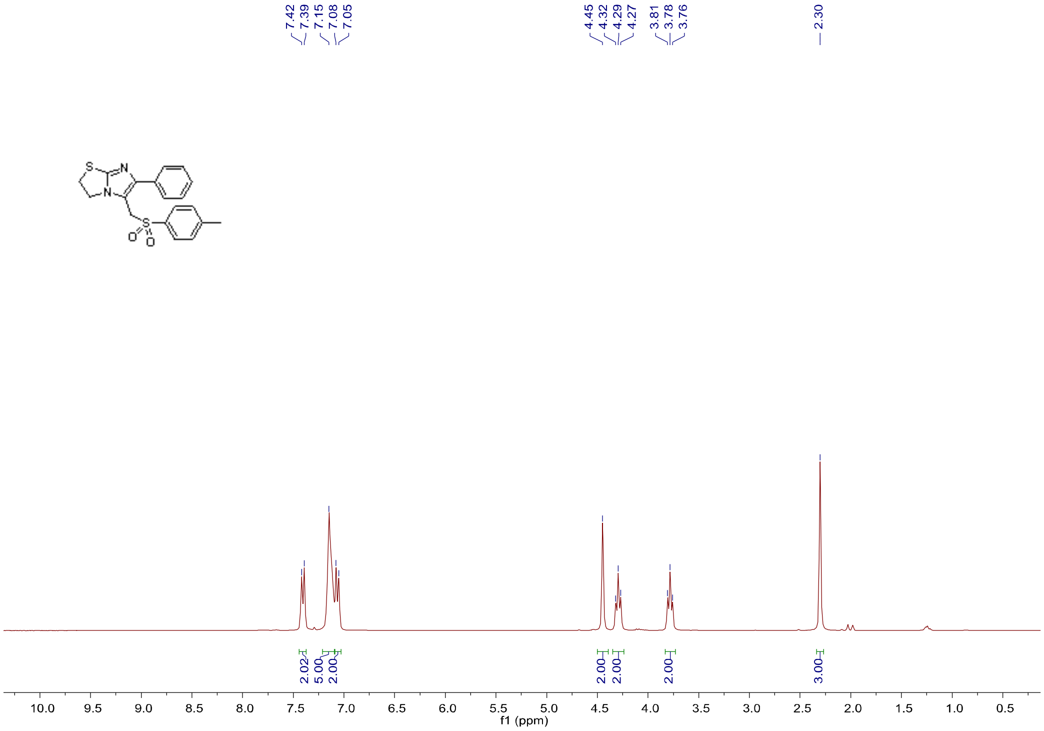

$^{13}C\{^{1}H\}$ (75 MHz, $CDCl_3$) NMR of **3ua**

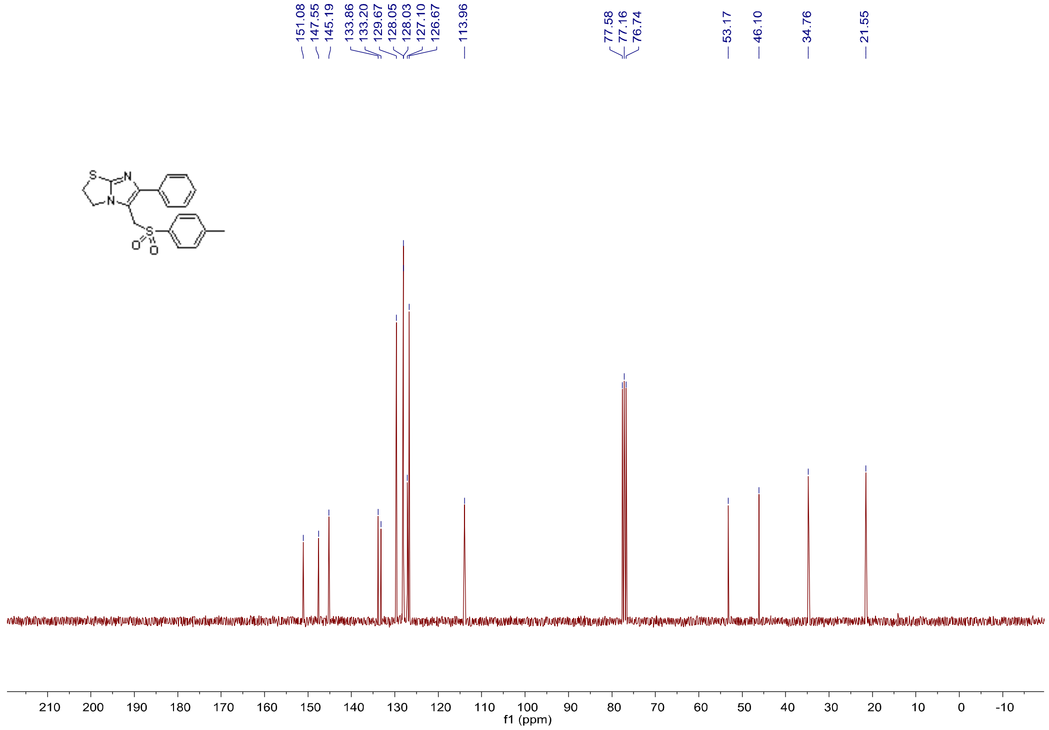

^{1}H NMR (300 MHz, $CDCl_3$) of **3ub**

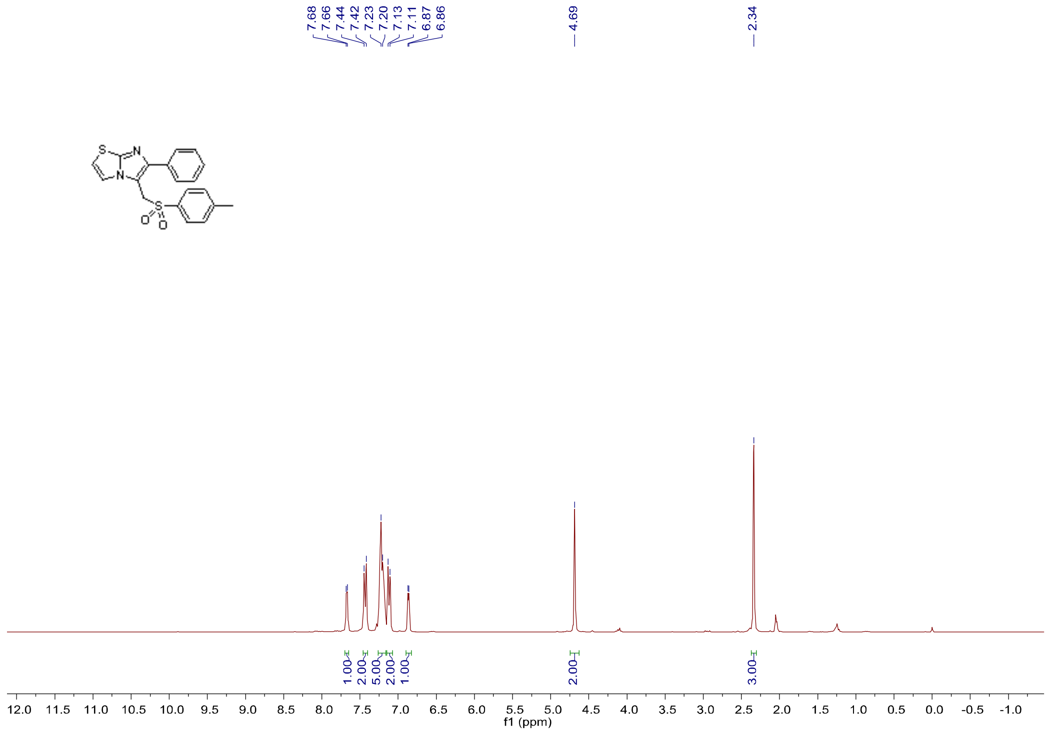

$^{13}C\{^{1}H\}$ (75 MHz, $CDCl_3$) NMR of **3ub**

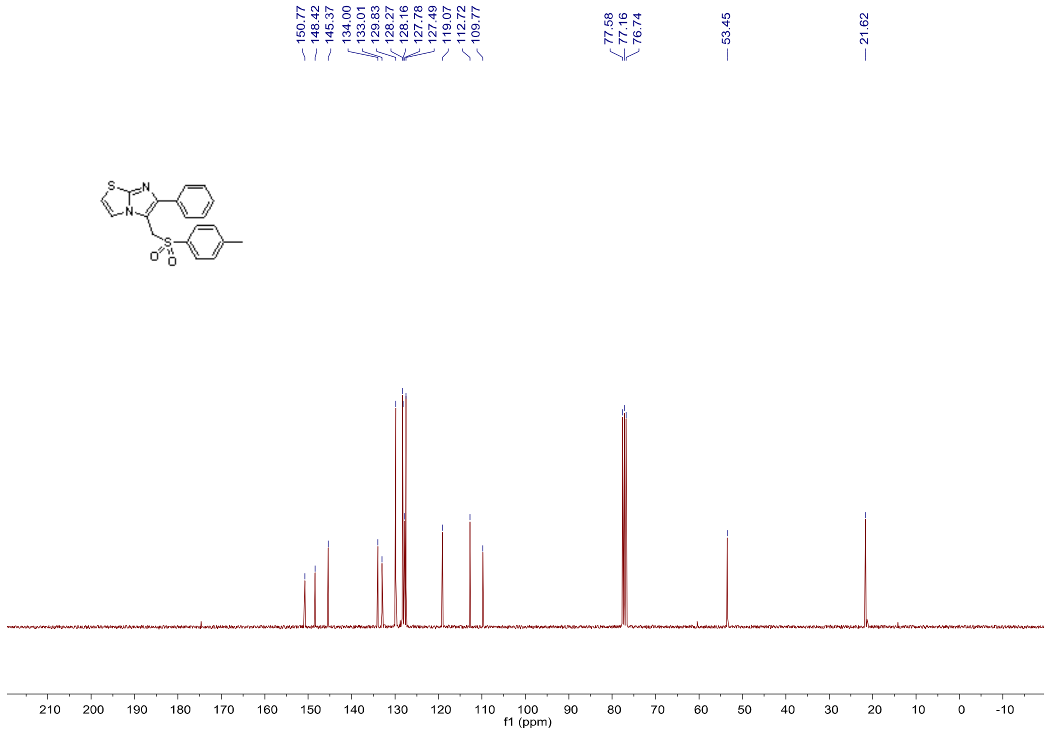

^{1}H NMR (300 MHz, $CDCl_3$) of **3uc**

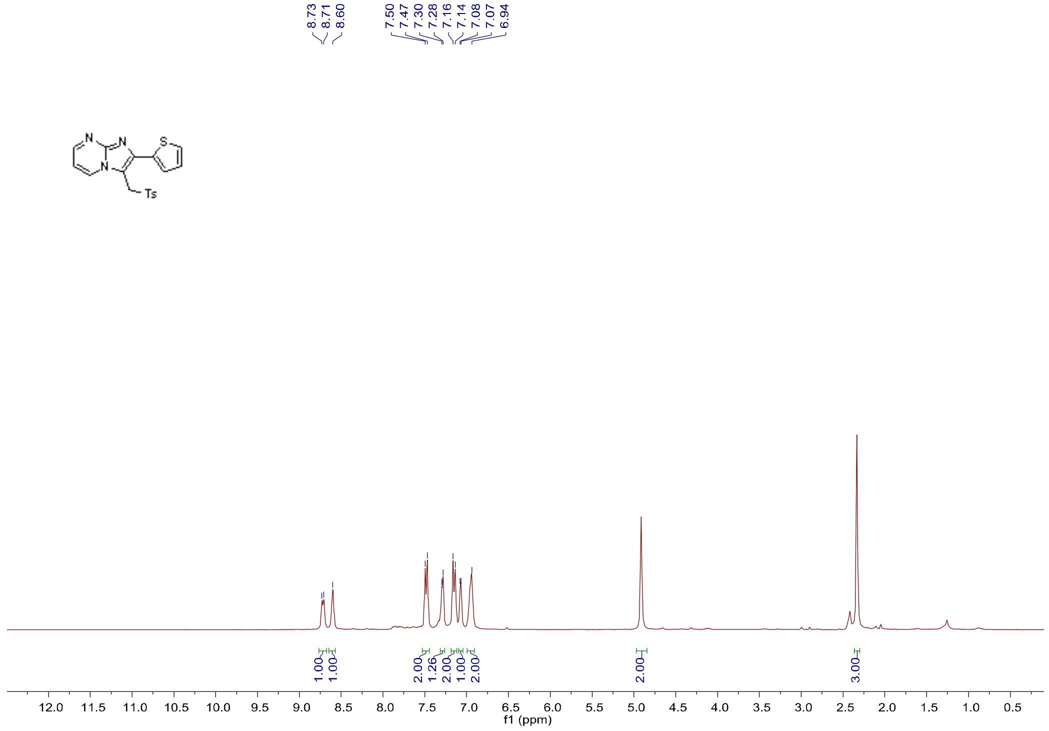

$^{13}C\{^{1}H\}$ (75 MHz, $CDCl_3$) NMR of **3uc**

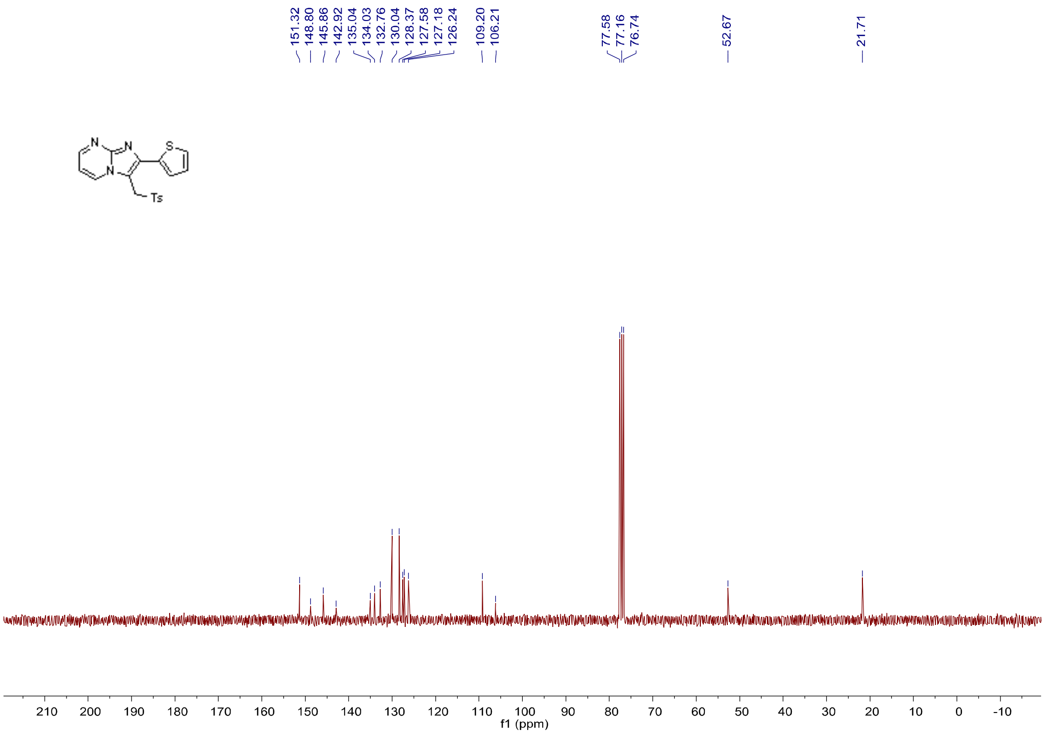

^{1}H NMR (300 MHz, $CDCl_3$) of **3ud**

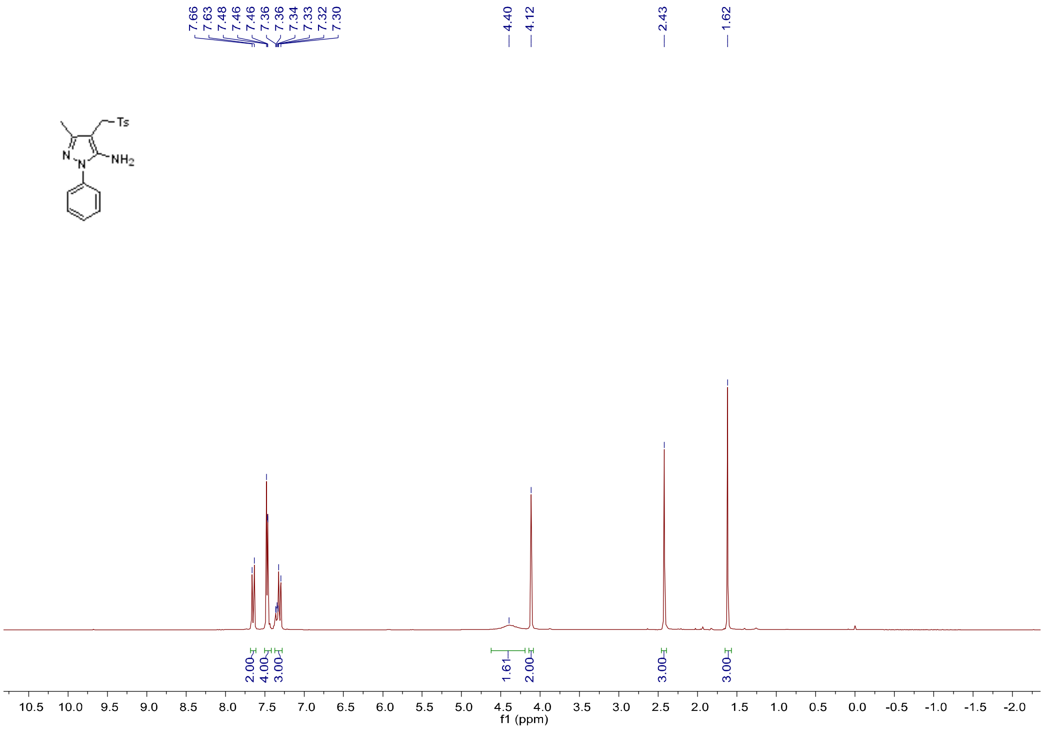

$^{13}C\{^1H\}$ (75 MHz, $CDCl_3$) NMR of **3ud**

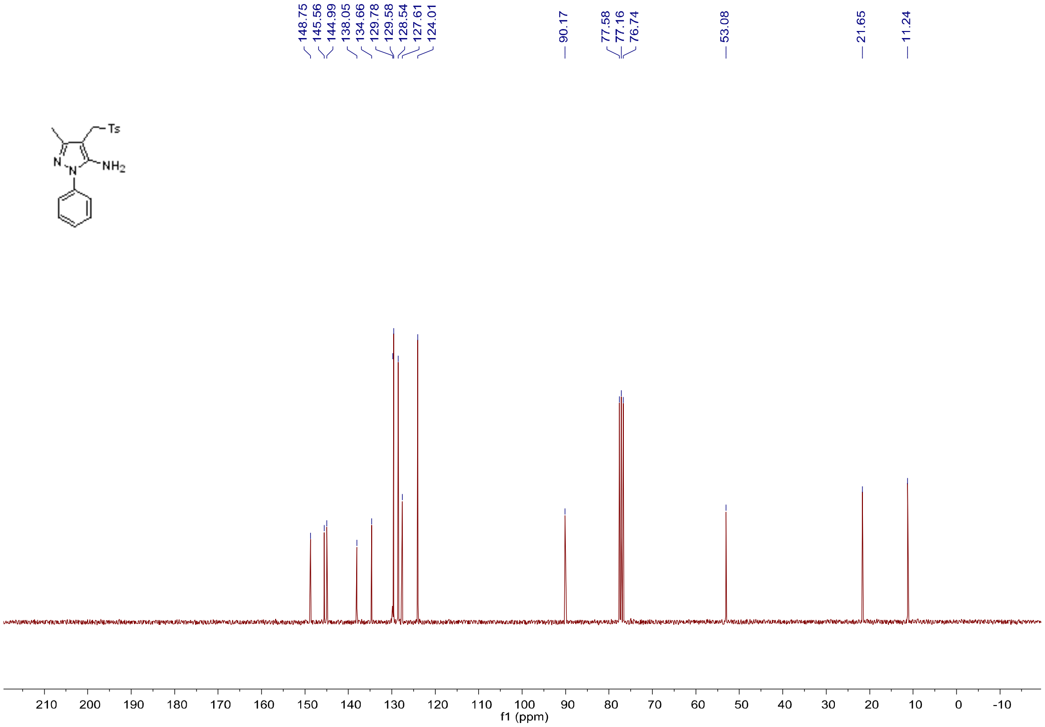

References

[1] J. -W. Chen, K. -M. Wen, Y. -R. Wu, J. Shi, X. -G. Yao, X. -D. Tang, *J. Org. Chem*. **2022**, *87*, 3780-3787.

[2] J. -W. Chen, J. -H. Tian, K. -M. Wen, Q. -W. Gao, J. Shi, X. -G. Yao, T. Wu, X. -D. Tang, *Org. Biomol. Chem*. **2022**, *20*, 1652-1655.
